# Network pharmacology combined with Mendelian randomization analysis to identify the key targets of renin-angiotensin-aldosterone system inhibitors in the treatment of diabetic nephropathy

**DOI:** 10.3389/fendo.2024.1354950

**Published:** 2024-01-25

**Authors:** Dongqi Zhou, Ting Zhou, Shiyun Tang, Qing Li, Wen Li, Gaofeng Gan, Mingqiao Li, Qiu Chen

**Affiliations:** ^1^ Department of Traditional Chinese Medicine, Taikang Hospital of Sichuan Province, Chengdu, Sichuan, China; ^2^ Chengdu University of Traditional Chinese Medicine, Chengdu, Sichuan, China; ^3^ Hospital of Chengdu University of Traditional Chinese Medicine, Chengdu, Sichuan, China; ^4^ Department of Endocrine, Hospital of Chengdu University of Traditional Chinese Medicine, Chengdu, Sichuan, China; ^5^ Department of Traditional Chinese Medicine and Orthopedics, Sichuan Provincial People’s Hospital, Chengdu, Sichuan, China

**Keywords:** diabetic nephropathy, GEO, network pharmacology, mendelian randomization, acute kidney injury

## Abstract

**Background:**

Diabetic Nephropathy (DN) is one of the microvascular complications of diabetes. The potential targets of renin-angiotensin-aldosterone system (RAAS) inhibitors for the treatment of DN need to be explored.

**Methods:**

The GSE96804 and GSE1009 datasets, 729 RAAS inhibitors-related targets and 6,039 DN-related genes were derived from the public database and overlapped with the differentially expressed genes (DN vs. normal) in GSE96804 to obtain the candidate targets. Next, key targets were screened via the Mendelian randomization analysis and expression analysis. The diagnostic nomogram was constructed and assessed in GSE96804. Additionally, enrichment analysis was conducted and a ‘core active ingredient-key target-disease pathway’ network was established. Finally, molecular docking was performed.

**Results:**

In total, 60 candidate targets were derived, in which *CTSC* and *PDE5A* were screened as the key targets and had a causal association with DN as the protective factors (*P* < 0.05, OR < 1). Further, a nomogram exhibited pretty prediction efficiency. It is indicated that Benadryl hydrochloride might play a role in the DN by affecting the pathways of ‘cytokine cytokine receptor interaction’, etc. targeting the *CTSC*. Moreover, *PDE5A* might be involved in ‘ECM receptor interaction’, etc. for the effect of NSAID, captopril, chlordiazepoxide on DN. Molecular docking analysis showed a good binding ability of benadryl hydrochloride and *CTSC*, NSAID and *PDE5A*. *PTGS2*, *ITGA4*, and *ANPEP* are causally associated with acute kidney injury.

**Conclusion:**

*CTSC* and *PDE5A* were identified as key targets for RAAS inhibitors in the treatment of DN, which might provide some clinical significance in helping to diagnose and treat DN. Among the targets of RAAS inhibitors, PTGS2, ITGA4 and ANPEP have a causal relationship with acute kidney injury, which is worthy of further clinical research.

## Introduction

Diabetic nephropathy is a disease characterized by a persistent increase in proteinuria and progressive elevation of blood pressure (1), and is also one of the most serious chronic microvascular complications of diabetes mellitus (2). About 50% of patients with DN eventually develop end-stage renal disease (ESRD) (3), and with the global diabetes epidemic, DN has gradually replaced other kidney diseases as the leading cause of ESRD (2, 4). DN has an insidious onset and early symptoms are not obvious (5), so it cannot be diagnosed by simple clinical signs (6). If DN progresses to ESRD, the only effective treatments for patients are dialysis and kidney transplantation (7), but these two treatments do not improve the survival prognosis of patients (8, 9). Therefore more and more researches are devoted to finding new therapeutic targets and diagnostic sites (10–12). The mechanisms of DN progression are complex and diverse, involving multiple pathways and mediators (13). Traditionally, the mechanism of development of DN is the result of abnormalities in body homeostasis, including hemodynamic abnormalities, metabolic disturbances, and imbalances in hormone synthesis, such as angiotensin II (Ang-II) (14). Although the exact pathogenesis of DN cannot be fully elucidated, studies have suggested that the renin-angiotensin-aldosterone system (RAAS), oxidative stress, and TGF-β are relatively common pathogenic mechanisms in the complex pathogenesis of DN (15, 16), and that a more comprehensive blockade of the RAAS may have more clinical benefits for DN (17, 18).

The RAAS is a complex network of multiple proteases and short peptides that regulate cardiovascular and renal function (19). RAAS inhibitors include angiotensin-converting enzyme inhibitors (ACEi), angiotensin II receptor antagonists (ARBs), aldosterone antagonists, and direct renin inhibitors (20). Recent studies have found that RAAS inhibitors significantly slow the progression of a wide range of diseases, including hypertension, myocardial remodeling after acute myocardial infarction, acute and chronic heart failure, and renal insufficiency, improving the prognosis of patients (21, 22). RAAS inhibitors dilate the glomerular outgoing and incoming small arteries to different degrees, and reduce the glomerular intraglomerular pressure leading to a decrease in urinary protein (23). Although RAAS inhibitors provide the rationale for current renoprotective therapies, there are limited data on whether early targeting of RAAS prevents kidney disease (24). Besides, nonproteinuric DN and DN without retinopathy in type 2 DM patients affects the detection of persistent albuminuria, so that RAAS inhibitors that target blocking albuminuria cannot be applied in time (25). ARBs reduce cardiovascular and renal complications in patients with DN, but the therapeutic effect may vary from patient to patient, and the exact regulatory mechanisms remain unclear (26). Previous studies involving ACEi have demonstrated beneficial effects on proteinuria, but it has not been demonstrated that blockade of the renin-angiotensin system is superior to non-blockade forms of therapy in slowing the progression of end-stage renal disease (27). Theoretically, the combination of ACEi and ARBs reduces proteinuria, but actually increases the risk of Acute kidney injury (AKI) and acute electrolyte disturbances (28). Combining the renin inhibitor aliskiren with an ACEI or ARB significantly increased the risk of hyperkalemia and did not reduce the risk of cardiovascular disease or renal failure (29). Therefore, there is an urgent need to further explore about the target mechanism of RAAS inhibitors acting in DN.

Mendelian randomization (MR) is a combination of the instrumental variables (IVs) method and Mendel’s laws of inheritance (30), which breaks through the limitations of traditional randomized controlled studies and avoids the interference of confounding factors (31). With the current development of basic Mendelian theory and the increase in practical applications, drug-targeted MR analysis is emerging as an effective tool for inferring the effects of drugs, antagonists, agonists, activators, or inhibitors targeting protein-coding genes on disease risk (32). In contrast to conventional MR analysis, drug-targeted MR analysis utilizes genetic variants in DNA sequences located within or near genes to predict the effect of the corresponding drug (33). Network pharmacology is an analysis method to visualize the correlation between drug components, targets and diseases (34). There are currently no studies on the causal relationship between the key targets of RAAS inhibitors and DN, and thus there is an urgent need for rational approaches to further elucidate the potential nature and significance of the above relationship.

**Figure 1:**
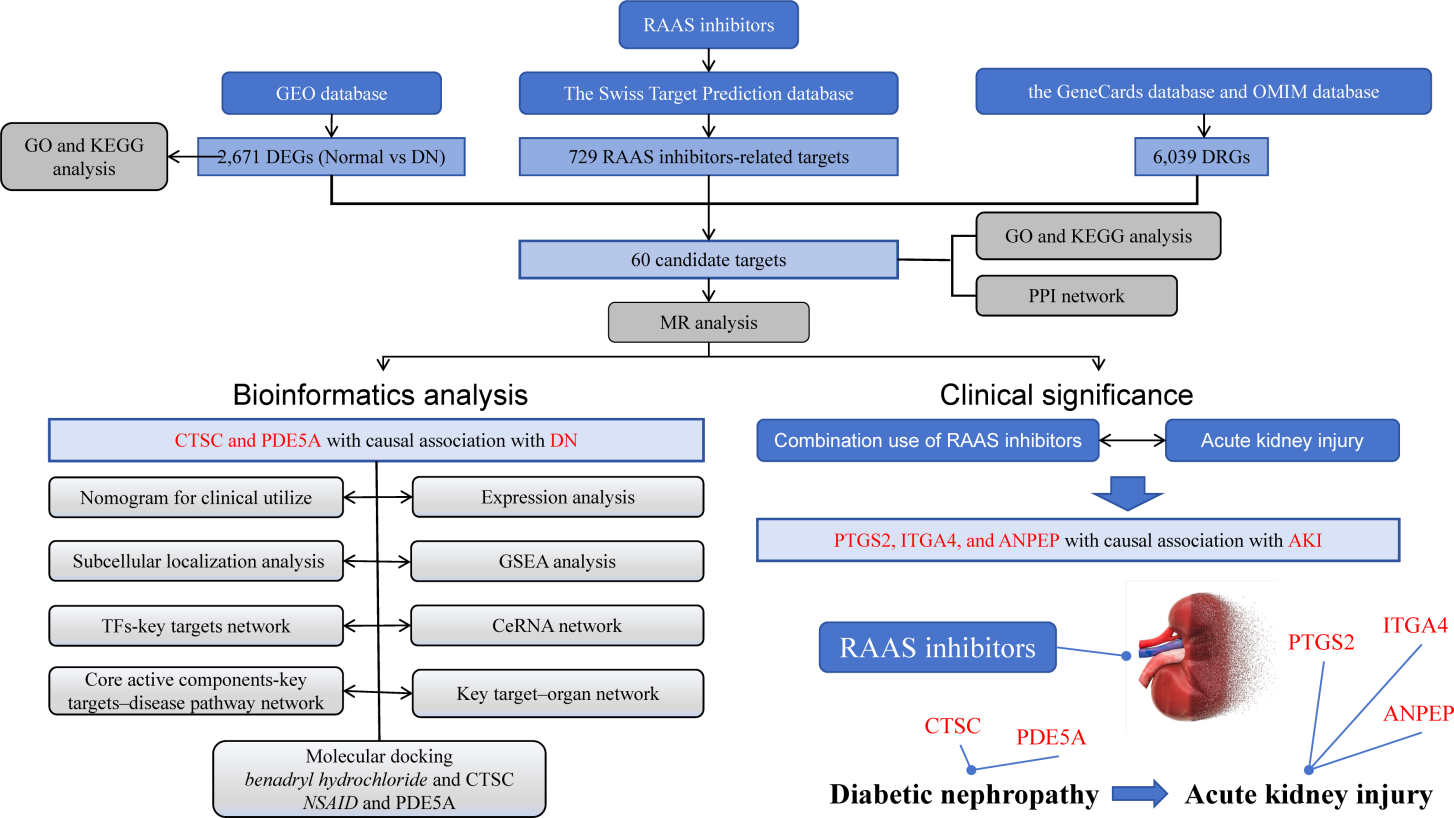
Graphical Abstract. The research process is illustrated in the diagram. The upper part represents the data included in the study, the bottom left corner represents the analysis involved in the research, and the bottom right corner represents the research findings.

In this study, we combined network pharmacology and drug-target MR for the first time to estimate the causal relationship between key targets of RAAS inhibitors and DN. Based on the DN-related data in the Gene Expression Omnibus (GEO) database and other public databases, we screened two key targets in the treatment of DN by RAAS inhibitors. The study further constructed a diagnostic nomogram, enrichment analysis and subcellular localization analysis of the key targets. Networks were constructed through network pharmacology, such as the Transcription Factors (TF) -Key target regulatory network and the competitive endogenous RNA (ceRNA) regulatory network. Finally, molecular docking was performed (**Figure 1** **Graphical Abstract**).

## Materials and methods

### Data source

The GSE96804 dataset, which included 41 type 2 DN samples (female) and 20 samples of unaffected parts of tumor nephrectomy, was applied as the training set (35). The GSE1009 dataset, which involved 3 DN samples and 3 normal samples, was considered as the validation set. All samples were derived from human renal glomerular tissues. The GSE96804 and GSE1009 datasets were downloaded from GEO database (https://www.ncbi.nlm.nih.gov/geo/). The components of RAAS inhibitors included aliskiren, enalapril, captopril, cilazapril, benazepril hydrochloride, enalapril maleate, valsartan, losartan, irbesartan, telmisartan, olmesartan, spironolactone, eplerenone, and fennelenone. RAAS inhibitors-related targets were identified by using the Swiss Target Prediction database. DN-related genes (DRGs) were screened via the GeneCards database and Online Mendelian Inheritance in Man (OMIM) database. The final DRGs were derived from the summary of the gene data that retrieved from the two databases and the removal of duplicate targets.

### Differential expression analysis

Differential expression analysis was carried out between normal and DN groups in the GSE96804 dataset via ‘Limma’ package (version 3.54.1) to obtain differentially expressed genes (DEGs) (36). Screening conditions were |log2FC| > 0.5 and *P* < 0.05. Candidate targets were derived from the intersection of DEGs, RAAS inhibitors-related targets and DRGs.

### Functional enrichment analysis and protein-protein interaction (PPI) network construction

In order to better explain the potential biological role of DEGs and candidate targets, Gene Ontology (GO) and Kyoto Encyclopedia of Genes and Genomes (KEGG) enrichment analysis was carried out via ‘clusterProfiler’ (version 4.0) in the GSE96804 dataset (37), and screening condition was *P* < 0.05. Moreover, in order to determine the interaction between candidate targets, a PPI network was established with confidence score > 0.4 via search tool for the retrieval of interacting genes (STRING) database (https://string-db.org/).

### Identification of key targets

Candidate targets that was causally associated with DN were screened via the MR analysis. The screening conditions were *P* < 0.05 for IVW method and *P* > 0.05 for Horizontal pleiotropy analysis. Next, the above targets were included in the GSE96804 and GSE1009 datasets for expression level analysis, and the targets with significant expression levels and consistent expression trends in two datasets were considered as key targets.

### MR analysis

In order to explore whether there was a causal relationship between key targets and DN, the key targets were considered as exposures, and DN was considered as the outcome for MR analysis. Considering the potential impact of RAAS inhibitors on acute renal injury, it is further proposed to use key targets as exposure factors and AKI as the outcome. The single nucleotide polymorphisms (SNPs), which had a significant link with exposures, were selected as IVs (*P* < 5 × 10^–8^) via ‘TwoSampleMR’ package in R (version 0.5.6) (38). Subsequently, the IVs with strong linkage disequilibrium (LD) were removed (r^2^ < 0.001, kb = 10000). The F-statistic values of SNPs were displayed in **Supplementary Table S1**. The effect alleles and effect quantities were unified via the R package ‘TwoSampleMR’ mv_harmonise_data function, and the mv_lasso_feature_selection function was devoted to eliminate the collinearity screening variables. Various MR approaches were used to confirm the causal associations between the key target genes and DN, containing the inverse variance weighted (IVW) (39), Mendelian randomization-Egger (MR-Egger) (40), weighted median (WM) (41), simple mode (42) and weighted mode methods (43), with IVW method predominating. *P* < 0.05 for IVW method was considered suggestive for the potential causal association. Furthermore, the odds ratios (ORs) were calculated. The value was greater than 1 being the risk factor and less than 1 being the protective factor. The scatter plot, forest plot and funnel plot were devoted to exhibit the results. Thereafter, the sensitivity analysis was devoted to estimate the reliability of the MR results via the Heterogeneity, Horizontal pleiotropy and Leave-One-Out (LOO) analysis. Moreover, the heterogeneity test was carried out and *P* > 0.05 demonstrated that there was no heterogeneity. *P* > 0.05 indicated that there was no horizontal pleiotropy in the horizontal pleiotropy test. LOO analysis was implemented by removing SNPs which were outliers.

### Construction of nomogram

In order to predict the prevalence rate of DN patients, a diagnostic nomogram was constructed in the GSE96804 dataset via the ‘RMS’ package (version 6.6-0) (44) based on key targets. The ability of the nomogram to predict DN was assessed via the calibration curves and decision curve analysis (DCA). Moreover, receiver operator characteristic (ROC) curve was plotted via the R package ‘pROC’ (version 4.0.5) (45) to assessed the prediction effect of the nomogram.

### Construction of a key target–organ network, subcellular localization analysis and enrichment analysis

In order to clarify the expression of key targets in the various organs and tissues, the expression of key targets in different organs was derived from the BioGPS database. The expression of the second abundant tissue was no more than one-third as a screening condition. The key target-organ network was constructed via the Cytoscape software (version 3.8.2) (46). Subcellular localization analysis of key targets was performed via ‘mRNALocater’ database, and protein sequences of key targets were downloaded from NCBI database. When the key targets were set as the objective genes, the correlation coefficients of the expression levels of all genes and the objective genes were calculated as the ranking criteria. Furthermore, Gene Set Enrichment Analysis (GSEA) was performed to explore the fuction of key targets via the ‘ClusterProfiler’ package (version 4.0) in the GSE96804 dataset (37). The screening condition was *P*.adj < 0.05. The most significant TOP5 pathway was selected for display.

### Constrution of ‘core active ingredient-key target-disease pathway’, TF-key targets and ceRNA network

In order to explore the relationship between ‘core active ingredient-key target-disease pathway’, active ingredients targeting key targets were selected as core active ingredients. The disease pathway were derive from the TOP5 KEGG pathway enriched by the key targets. TFs associated with key targets were predicted via the ChEA3 database. Moreover, the miRNAs associated with key targets were forecasted by Starbase database (https://starbase.sysu.edu.cn/index.php), and the screening criterion was clipExpNum > 8. The lncRNAs associated with miRNAs were predicted by Starbase database, and the screening criterion was clipExpNum > 13. The ‘Cytoscape’ software (version 3.8.2) was devoted to establish the ‘core active ingredient-key target-disease pathway’, ‘TF-key targets’ network and ‘lncRNA-miRNA-mRNA’ network (46).

### Molecular docking

In order to determine the binding ability between the core active components and key targets, molecular docking was conducted via the AutoDock Vina (version 4.2) (47). Briefly, the docking was as follows. 1) The 3D structure of the key target protein was retrieved from the RSCB PDB database and downloaded with the PDB file format, and then non-polar hydrogen was added to the three-dimensional structure using AutoDockTools software to calculate the charge and saved it as a PDBQT file as a pair of receptors. 2) The SDF format file of the 2D structure of the core active ingredient was retrieved and downloaded from PubChem, and then converted into the mol3 format file of the 3D structure via the ChemBio2D software. Then the ligand mol2 file was integrated into AutoDockTools, and the file was output to the PDBQT format file as a docking ligand. 3) Molecular docking of key targets and core active components was conducted via the ‘AutoDock Vina’, and the free binding energy was evaluated. 4) PyMOL and Discovery Studio software were used to visualize the molecular docking results.

### Statistical analysis

Statistical tests were carried out via the R software (version 4.2.2). Statistical significance was defined as *P* < 0.05.

## Results

### Identification of DEGs and functional enrichment analysis in the GSE96804 dataset

**Figure 2:**
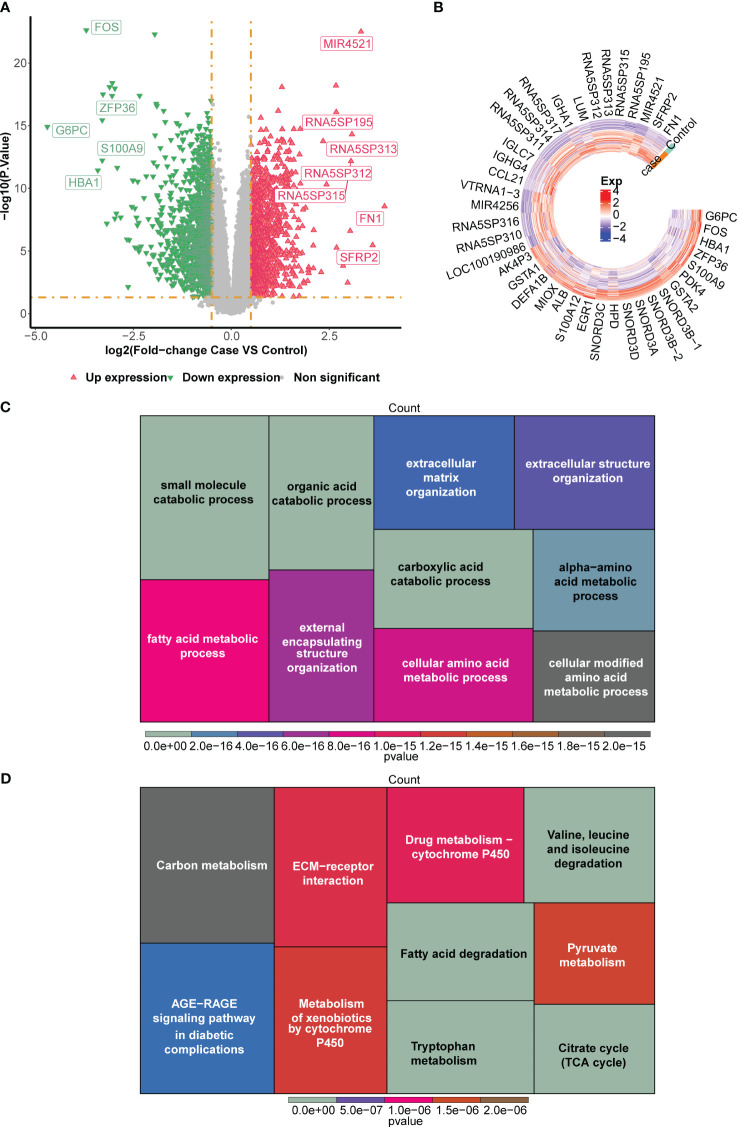
Screening of differentially expressed genes (DEGs) between normal and DN groups in the GSE96804 dataset. **(A)** The volcano plot and **(B)** heatmap for the expression patterns of the DEGs. The heatmap for the enriched **(C)** Gene Ontology (GO) terms and **(D)** Kyoto Encyclopedia of Genes and Genomes (KEGG) terms by the DEGs.

A number of 2,671 DEGs were discovered between normal and DN groups in GSE96804 dataset, which included 1,278 up-regulated genes and 1,393 down-regulated genes. Volcano plot and heatmap were plotted to visualize these DEGs (**Figures 2A, B**). In order to better explain the potential biological role of DEGs, GO and KEGG enrichment analysis were conducted. GO results showed that DEGs were mainly involved in the ‘small molecule catabolic process’ and ‘alpha−amino acid metabolic process’ (**Figure 2C**). KEGG enrichment analysis revealed that DEGs mainly involved in ‘Fatty acid degradation’ and ‘AGE−RAGE signaling pathway in diabetic complications’ (**Figure 2D**).

### Identification of candidate genes and exploration of potential biological functions

**Figure 3:**
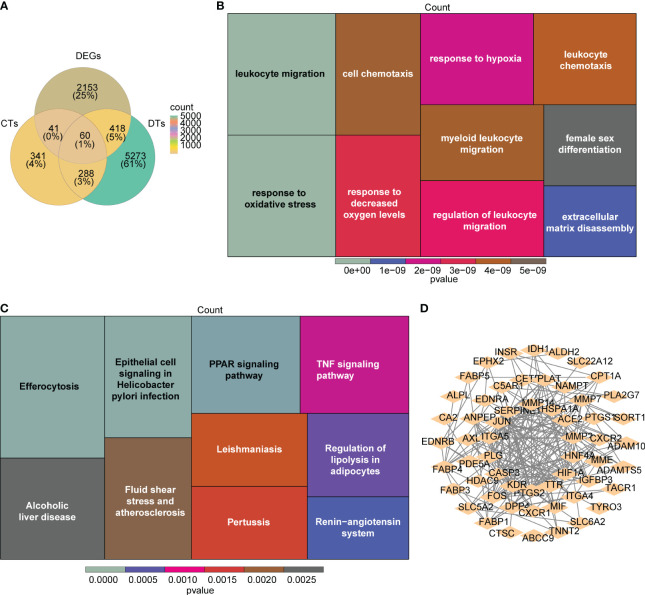
Identification and interaction of potential candidate genes in DN. **(A)** The venn plots of the intersection for DEGs, renin-angiotensin-aldosterone system (RAAS) inhibitors-related targets, and DN-related genes (DRGs). **(B, C)** The heatmap for the functional enrichment analysis (B:GO; C:KEGG) of the 60 candidate genes. **(D)** The protein-protein interaction (PPI) network for interaction of candidate genes with confidence score > 0.4.

A total of 729 RAAS inhibitors-related targets were identified via the Swiss Target Prediction database. A number of 6,039 DRGs were discovered. A number of 60 candidate genes were derived by intersecting DEGs, RAAS inhibitors-related targets and DRGs (**Figure 3A**; **Supplementary Table S2**). In addition, GO results showed that candidate genes were involved in ‘leukocyte migration’ and ‘response to oxidative stress’ (**Figure 3B**). KEGG result revealed that candidate genesmainly involved in ‘Renin−angiotensin system’ and ‘PPAR signaling pathway’ (**Figure 3C**). A PPI network, which contained 57 nodes and 216 edges was constructed, in which *CASP3*, *ITGA5* and *MMP14* were more important (**Figure 3D**).

### *CTSC* and *PDE5A* were viewed as protective factors for DN

**Figure 4:**
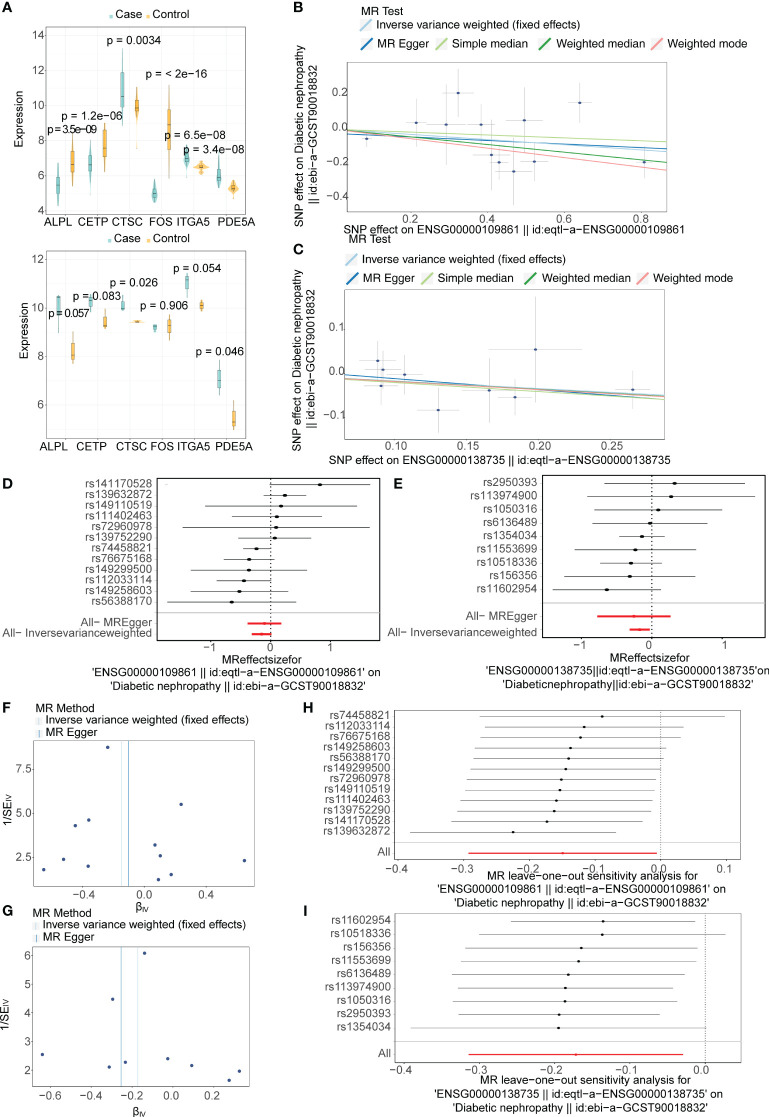
Mendelian randomization (MR) analysis and expression analysis for selecting two key targets in DN. **(A)** Boxplots for the expressions levels of six candidate targets with potential causality on DN in the GSE96804 (top) and GSE1009 (bottom) datasets. **(B, C)** The scatter plot of the Mendelian randomization (MR) analysis for relationship of two key targets (B:*CTSC*; C:*PDE5A*) and DN. **(D, E)** Forest plots of the MR analysis for diagnostic significance of two key targets (D:*CTSC*; E:*PDE5A*) on DN. **(F, G)** Funnel plots of the MR analysis for two key targets (**F**:*CTSC*; **G**:*PDE5A*) on DN. **(H, I)** Leave-one-out analysis of the MR analysis for sensitivity analyses of two key targets (H:*CTSC*; I:*PDE5A*) on DN.

A total of six candidate targets that was causally associated with DN, which were *ALPL*, *CETP*, *CTSC*, *FOS*, *ITGA5* and *PDE5A* were screened with *P* < 0.05 for IVW method and *P* > 0.05 for Horizontal pleiotropy analysis (**Table 1**). Furthermore, *CTSC* and *PDE5A* were selected as the key targets. The expression level of *CTSC* and *PDE5A* were significantly high in the CCA group (**Figure 4A**). *CTSC* and *PDE5A* were causally associated with DN by IVW approach, and they were the protective factors for DN (*P* < 0.05, OR < 1) (**Table 2**). The scatter plot revealed that *CTSC* and *PDE5A* were negatively correlated with DN (slope < 0) (**Figures 4B, C**). In the forest plot, the MR effect size was less than 0, indicating that *CTSC* and *PDE5A* were the protective factors for DN (**Figures 4D, E**). The funnel plot of two genes exhibited that the MR analysis conformed to the random grouping of Mendel’s second law (**Figures 4F, G**). In order to evaluate the reliability of MR results, the sensitivity analysis was carried out. The *P* value of the Cochrane’s Q test was greater than 0.05, indicating that there was no heterogeneity between the two sample datasets of exposures and outcome (**Table 3**). Meanwhile, the *P* value of the horizontal pleiotropy test was greater than 0.05, indicating that there was no interference of confounding factors (**Table 4**). LOO analysis revealed that there was no significant deviation in the effect value of the IVs (**Figures 4H, I**).

**Table 1:** Information for six candidate targets causally associated with diabetic nephropathy (DN).

id.exposure	id.outcome	method	nsnp	pval	pleio_pval
eqtl-a-ENSG00000162551	ebi-a-GCST90018832	IVW	5	0.017	0.762
eqtl-a-ENSG00000087237			5	0.015	0.823
eqtl-a-ENSG00000109861			12	0.041	0.693
eqtl-a-ENSG00000170345			3	0.003	0.661
eqtl-a-ENSG00000161638			5	0.000	0.667
eqtl-a-ENSG00000138735			9	0.018	0.746

IVW, Inverse variance weighted.

**Table 2:** Mendelian randomization (MR) analysis for causal relationship of two key targets (*CTSC* and *PDE5A*) and DN.

outcome	exposure	Method	Pvalue	OR
ebi-a-GCST90018832	eqtl-a-ENSG00000109861(CTSC)	MR Egger	0.492	0.902
		IVW	0.041	0.861
		Weighted median	0.025	0.802
		Simple mode	0.680	1.090
		Weighted mode	0.024	0.761
	eqtl-a-ENSG00000138735 (PDE5A)	MR Egger	0.371	0.776
		IVW	0.018	0.842
		Weighted median	0.171	0.835
		Simple mode	0.331	0.814
		Weighted mode	0.231	0.832

IVW, Inverse variance weighted; MR-Egger, Mendelian randomization-Egger; WM, weighted median.

**Table 3:** Results for heterogeneity test.

outcome	exposure	method	Q	Q_df	Q_pval
ebi-a-GCST90018832	eqtl-a-ENSG00000109861 (CTSC)	MR Egger	14.049	10	0.171
		Inverse variance weighted	14.280	11	0.218
	eqtl-a-ENSG00000138735 (PDE5A)	MR Egger	3.689	7	0.815
		Inverse variance weighted	3.803	8	0.874

**Table 4:** Results for horizontal pleiotropic test.

	exposure	egger_intercept	se	pval
ebi-a-GCST90018832	eqtl-a-ENSG00000109861 (CTSC)	-0.024	0.059	0.693
	eqtl-a-ENSG00000138735 (PDE5A)	0.014	0.041	0.746

### Diagnostic value of *CTSC* and *PDE5A* in DN

**Figure 5:**
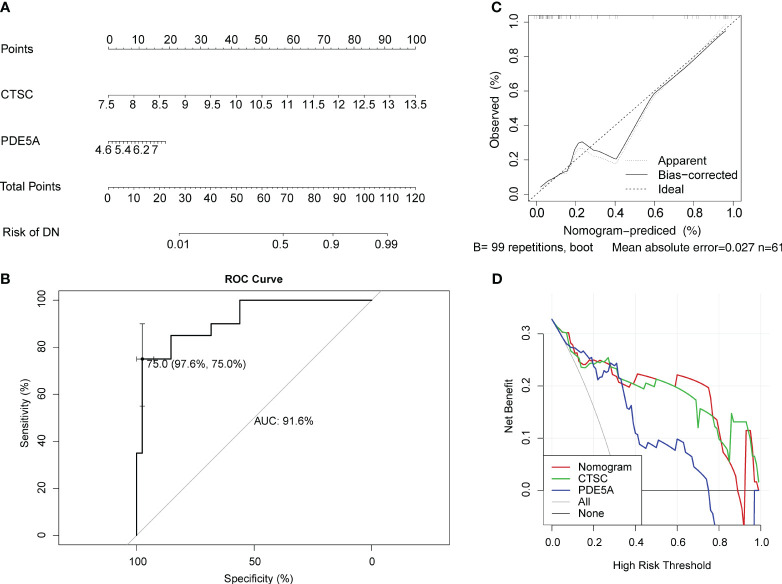
Evaluation for clinical utilize of *CTSC* and *PDE5A* via a nomogram. **(A)** Nomogram for predicting the risk of DN. **(B)** Receiver operator characteristic (ROC) curve evaluating the predictive accuracy of nomogram in GSE96804. **(C)** Calibration curves of nomogram for comparing predicted (the horizontal coordinate) and actual (the vertical coordinate) probability of DN. The 45-degree line represents the ideal prediction. **(D)** Decision curve analysis (DCA) for the GSE96804 dataset.

In order to predict the prevalence rate of DN patients, the diagnostic nomogram was established on the basis of key targets in the GSE96804 dataset (**Figure 5A**). In the GSE96804, the AUC value of ROC curve for normogram was greater than 0.7, indicating that predictive accuracy of nomogram was high (**Figure 5B**). The slope of the calibration curve was close to 1, and it demonstrated that nomogram had pretty prediction efficiency (**Figure 5C**). Moreover, the results of DCA showed that the net income of the nomogram was higher than a single factor. It also reflected the pretty prediction effect of the nomogram (**Figure 5D**).

### Exploring potential binding sites for *CTSC* and *PDE5A*

**Figure 6:**
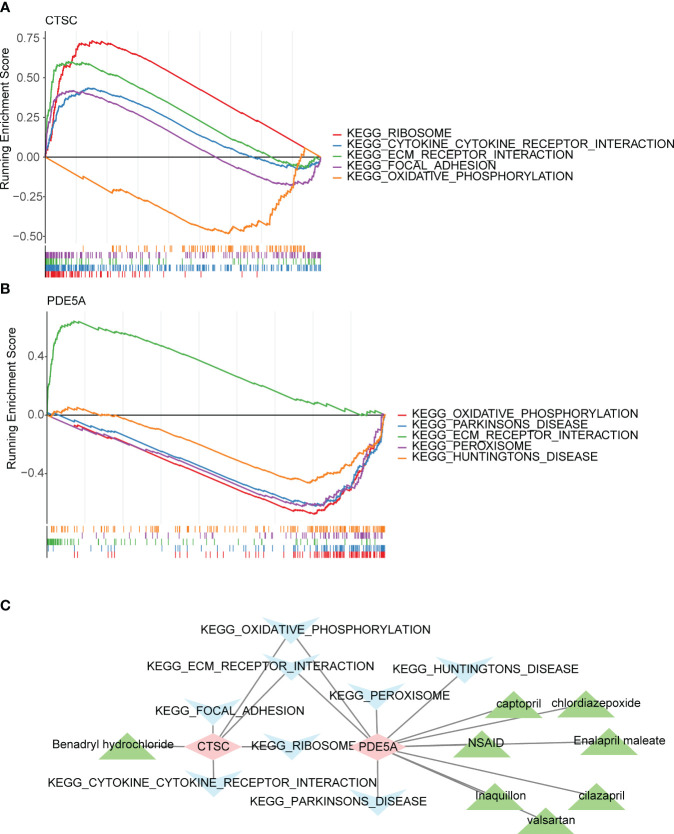
Functionality, active ingredients and disease pathway targeting two key targets. Gene set enrichment analysis (GSEA) of **(A)** *CTSC* and **(B)** *PDE5A*. **(C)** The core active ingredient-key target-disease pathway network. Pink is the key target gene, blue is the disease pathway, and green is the core active ingredient.

The mRNA levels of the *CTSC* and *PDE5A* were evaluated. In total, 12 organs or tissues were associated with *CTSC*, including the lung, smooth muscle and CD56+_NK Cells. A number of 11 organs or tissues were associated with *PDE5A*, containing adrenal gland, heart and liver (**Figure S1**). Subcellular localization analysis showed that *CTSC* had the highest proportion in cytoplasm and *PDE5A* had the highest proportion in nucleus (**Figure S2**). *CTSC* was significantly involved in ‘cytokine cytokine receptor interaction’, ‘ribosome’, ‘ECM_receptor_interaction’, ‘focal_adhesion’ and ‘oxidative phosphorylation’ (**Figure 6A**). Benadryl hydrochloride might play a role in the DN by affecting these pathways through the *CTSC*. Moreover, *PDE5A* was significantly involved in ‘parkinsons disease’, ‘peroxisome’, ‘huntingtons disease’, ‘ECM receptor interaction’ and ‘oxidative phosphorylation’ (**Figure 6B**). NSAID, captopril, chlordiazepoxide, Enalapril maleate, cilazapril, valsartan and Inaquillon might play roles in the DN by affecting these pathways through the *PDE5A* (**Figure 6C**).

### Investigation of regulatory mechanisms between key targets and other types of molecules

**Figure 7:**
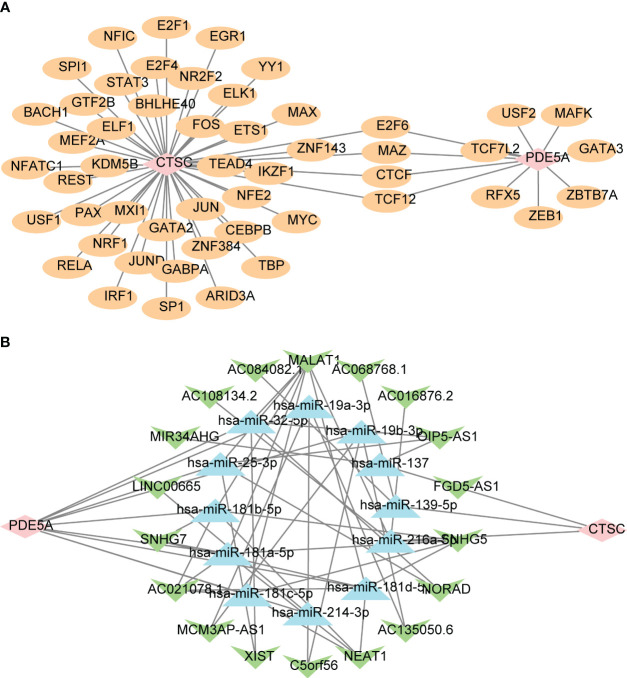
Regulatory networks of two key targets. **(A)** The transcription factors (TF)-key targets network. Pink is the target gene, and yellow is TF. **(B)** The competitive endogenous RNA (ceRNA) network targeting key targets. Pink is the key target gene, green is lncRNA, and blue is miRNA.

To further explore the potential mechanism of *CTSC* and *PDE5A*, we predicted their targeted TFs, miRNAs, and lncRNAs by online databases and constructed regulatory networks. In total, 44 TFs that associated with *CTSC* were prediceted, a number of 11 TFs that associated with *PDE5A*, in which *CTSC* and *PDE5A* were regulated by E2F6, MAZ, CTCF and TCF12 (**Figure 7A**). Moreover, a ceRNA network, that contained 12 miRNAs, 45 lncRNAs, *CTSC* and *PDE5A* was established, in which SNHG5 regulated *CTSC* by hsa-miR-216a-5p, SNHG5 regulated *PDE5A* by hsa-miR-181a-5p (**Figure 7B**).

### Prediction of active ingredient-target binding capacity by molecular docking

**Figure 8:**
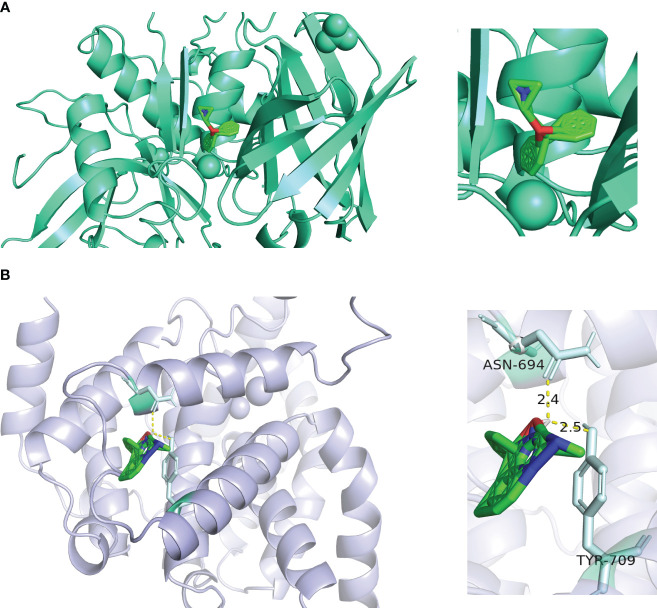
Molecular docking results of two key targets. **(A)** *CTSC*. **(B)** *PDE5A*.

In order to determine the binding ability between the core active components and key targets, molecular docking was carried out. Molecular docking analysis showed that the docking affinity between core active ingredients benadryl hydrochloride and *CTSC* was - 5.3 kcal/moL, indicating that the binding ability was good (**Figure 8A**). The core active ingredients NSAID formed a covalent bond with the *PDE5A*. The docking affinity between NSAID and *PDE5A* was - 5.32 kcal/moL, indicating that the binding ability was good (**Figure 8B**).

### PTGS2, ITGA4, and ANPEP are causally associated with AKI.

To investigate potential factors contributing to renal injury observed in clinical coadministration of RAAS inhibitors, we employed Mendelian Randomization analysis of the core targets of RAAS inhibitors on AKI. Our findings indicate a causal relationship between PTGS2, ITGA4, and ANPEP and AKI (**Table 5**; **Supplementary Information 2**).

**Table 5:** Mendelian randomization (MR) analysis for causal relationship of three key targets (PTGS2, ITGA4, and ANPEP) and AKI.

outcome	exposure	Method	Pvalue	OR
ukb-b-4963	eqtl-a-ENSG00000166825 (ANPEP)	MR Egger	0.000	0.000
		IVW	0.000	3.82E-07
		Weighted median	0.000	-4.78E-06
		Simple mode	0.000	0.001
		Weighted mode	0.000	4.44E-06
	eqtl-a-ENSG00000115232 (IGTA4)	MR Egger	0.637	0.9998
		IVW	0.000	0.9997
		Weighted median	0.128	0.9997
		Simple mode	0.350	0.9995
		Weighted mode	0.320	0.9997
	eqtl-a-ENSG00000073756(PTGS2)	MR Egger	0.320	1.001
		IVW	0.004	1.001
		Weighted median	0.007	1.001
		Simple mode	0.169	1.001
		Weighted mode	0.081	1.001

## Discussion

Clinicians have long been accustomed to treating DN with RAAS inhibitors to reduce urinary protein leakage in patients (48, 49). RAAS inhibitors are known to modulate DN perfusion by dilating small glomerular arterioles, which is why they reduce proteinuria. The combination of renin inhibitors, ARBs and ACEi can inhibit Ang II faster and more comprehensively (50), so blocking the multiple pathways of action of RAAS is expected to increase the efficacy of DN. However, excessive inhibition of Ang II will cause the glomerular outflow arterioles to dilate more than the inlet arterioles, thus increasing the risk of renal damage. This may be one of the explanations for the increased risk of acute renal failure found with RAAS inhibitor combination in several large clinical studies (28, 29). Then, the molecular mechanism of the RAAS inhibitor co-administration process in renal tissues remains unknown, and it is still debatable whether there are unknown targets involved in the regulatory process of DN. On the other hand, although the use of RAAS inhibitors did effectively reduce cardiovascular events and inhibit the progression of DN, there is no evidence that RAAS inhibitors reduce renal endpoint events (51). Drug-targeted Mendelian studies are mostly modeling the therapeutic effect of a single drug on a particular disease, making it difficult to break through to how a combination of drugs affects the disease (52). The method of combining drug-targeted Mendelian analysis through network pharmacology may be an effective means to end this problem.

Through our study, We firstly observed that CTSC and PDE5A were causally associated with DN. According to network analysis, both targets were core targets of RAAS inhibitors acting on DN. We analyzed the expression of CTSC, PDE5A in the GSE96804 and GSE1009 between DN and normal groups. The results showed significant and consistent expression trends of CTSC and PDE5A. Besides, the PTGS2, ITGA4, and ANPEP with causal association with AKI were investigated, providing more theoretical perspectives for the combined use of RAAS inhibitors to promote the risk of AKI.

CTSC encodes for a lysosomal cysteine protease. Rare mutations in the gene cause autosomal-recessive PapillonLefèvre syndrome (53). It is well known that kidney damage does not occur in all diabetics. This suggests that neither the genetic variant itself nor hyperglycemia is sufficient to cause the typical proteinuric kidney damage in DN (54). So what exactly causes DN to occur in diabetics, and the increased risk of renal failure? A previous genome-wide association studies (GWAS) and meta-analysis (comprising 20 studies, 54,450 participants, 2,191,945 SNPs) has confirmed that RAB38/CTSC and HS6ST1 were human DN urinary protein genes (55). Study has indicated that RAB38/CTSC and HS6ST1 are involved in renal regulation of albumin and are associated with proteinuria in diabetic patients. It is worth mentioning that exposure to the environment is also important in diabetic patients, and the above feature (proteinuria in diabetic patients) is more significantly observed when environmental exposure and genetic susceptibility variants occur together (54). But due to the intergenic index SNP mapped upstream of RAB38 and downstream of CTSC and was associated with transcript levels of both genes in whole blood (55). Thus, the exact molecular mechanism and causality of the RAB38/CTSC variants associated with human proteinuria remain unproven. Considering the effectiveness of RAAS inhibitors in clinical control of urinary protein in patients with DN. It is reasonable to speculate that CTSC may indeed be involved in human urinary protein regulation, which provides a theoretical basis for the previous question.

Subcellular localization analysis revealed that CTSC had the highest percentage in Cytoplasm. Enrichment analysis indicated that CTSC was significantly involved in ‘cytokine cytokine receptor interaction’, ‘ribosome’, ‘ECM_receptor_interaction’, ‘focal_adhesion’ and ‘oxidative phosphorylation’. We used molecular docking to explore the interaction of RAAS inhibitor actives with CTSC at the molecular level, and found that Benadryl hydrochloride interacts with CTSC (molecular binding energy for both is -5.3 kcal/mol). This suggests that Benadryl hydrochloride may play a role in DN by influencing the above pathways through CTSC, whereas the research of Benadryl hydrochloride in the treatment of DN is still relatively limited. Likewise, there is few studies on the functional mechanism between CTSC and DN, various challenges need to be overcome in combination with the predicted functionality-related clues, and the sufficient clinical samples and the suitable animal models need to be further collected and analyzed.

Phosphodiesterases (PDE) are a superfamily of enzymes (PDE1-PDE11) that hydrolyze cyclic adenosine monophosphate (cAMP) and cyclic guanosine monophosphate (cGMP). Therefore, PDE play key roles in intracellular signaling (56). PDE5A belongs to the PDE family, and targeted regulation of PDE5A is considered an effective target for the treatment of cardiovascular disease (57). Indeed, the PDE5 inhibitor sildenafil, which has been approved for clinical use, has been shown to ameliorate diabetic renal podocyte injury, proteinuria, and renal fibrosis (58). Targeted regulation of PDE5 further modulates miR-22 and BMP7 to improve renal hemodynamics and function in DN mice (59). Recent study has suggested that the combination of PDE5 inhibitors with irbesartan is recommended for the treatment of DN because targeted modulation of PDE5 shows anti-renal fibrillary capacity and nephroprotection (58). The current study confirmed some DN therapeutic effects through PDE5 inhibitors, but the mechanism was reported to be related to the reduction of cGMP catabolism or maintenance of cGMP concentration (60, 61). However, in our study, we observed that RAAS inhibitors targeting modulation of PDE5A was a protective factor for DN, which seems to be different from the results of previous studies. As mentioned before, PDE5A is also involved in renal hemodynamic alterations, and excessive reduction of renal entry arterial blood by the RAAS rather increases the risk of renal damage (59). Combined with the fact thatRAAS inhibitors can act on PDE5A in the present study. Therefore, the effect of RAAS inhibitors on PDE5A modulation on DN deserves to be investigated in depth.

Enrichment analyses showed that PDE5A was clearly involved in ‘ECM receptor interaction’, ‘Parkinson’s disease’, ‘peroxisome’, ‘Huntington’s disease’ and ‘oxidative phosphorylation’(**Figure 6B**). NSAID, captopril, chlordiazepoxide, enalapril maleate, cilazapril, valsartan, and Inaquillon might play roles in DN by affecting these pathways through PDE5A. Both CTSC and PDE5A are associated with oxidative stress and phosphorylation. Whereas oxidative stress is a central factor in the development of DN (62). Phosphorylation is involved in the metabolism of glycosylation end products and thus affects DN (63). Therefore, it is worthwhile to pay attention about the target regulation of CTSC and PDE5A in DN.

As we have discussed in the background section, the current diagnosis of DN is also a major clinical challenge. Earlier clinical symptoms of DN were so mild that leakage of urinary microalbumin did not alert patients to early intervention, especially in patients with type 2 DN. Renal puncture is the gold standard for clarifying the presence of DN pathologic changes, but it is not practical to perform renal puncture in every diabetic patient (64). In addition, for patients with DN who already have urinary protein, intensive glycemic control is not effective in reversing proteinuria but rather increases the risk of severe hypoglycemia (65). The results of the expression of CTSC and PDE5A in DN patients significantly differed from that in the control group showed that the expression level of CTSC and PDE5A was closely related to disease progression. Monitoring the expression of CTSC and PDE5A may be helpful in the diagnosis of type 2 DN. The nomogram prediction model is a common and effective tool by inverting the expression into a total score to clarify the association between predictors and the risk of disease, and can enhance the practicality of gene expression monitoring for clinical decision-making. The diagnostic value of key targets for DN was evaluated in our study by constructing diagnostic nomogram. Combined with the results, CTSC and PDE5A can be used as potential diagnostic targets for DN. This provides new ideas and evidence for clinical DN diagnosis.

Currently, it is believed that ACEi/ARB medications have a greater dilation effect on the arterioles that carry blood away from the glomerulus compared to the arterioles that carry blood towards the glomerulus. When used concomitantly, this may cause a decrease in glomerular filtration pressure, resulting in insufficient pressure within the glomerulus and reduced blood flow, ultimately leading to impaired kidney function. However, further evidence is required to fully explain the heightened risk of developing acute kidney injuries when renin inhibitors are combined with ACEi/ARB medications. Theoretically, combining RAAS inhibitors offers better control of urinary protein in patients with DN, but empirical data suggests that there are more intricate changes at play that need to be investigated. In our study, we discovered that out of the 60 targets affected by RAAS inhibitors in DN, three (PTGS2, ITGA4, ANPEP) had a significant causal relationship with acute renal failure. These findings may provide additional insights into the current therapeutic challenges associated with RAAS inhibitors in DN.

PTGS2, also referred to as cyclooxygenase-2 (COX-2), is an enzyme implicated in the inflammatory response. Its primary role is to facilitate the conversion of arachidonic acid to prostaglandin H2, which serves as a catalyst for an inflammatory response (66). PTGS2 expression is influenced by a range of factors, with stress being a significant regulatory element (67). Enzymes associated with PTGS2 are present in various sites within the mammalian kidney, including dense plaques, medullary interstitial cells, arteriolar endothelium, and glomerular podocytes (68). Consequently, targeted adjustment of PTGS2 could be a viable approach for treating renal diseases. A PTGS2 inhibitor (celecoxib) was previously linked to acute kidney damage and substantial urinary protein loss in a study (69). Another study reported celecoxib as a cause of acute renal failure and hyperkalemia, with recovery observed upon discontinuation of the drug (70). In mammalian kidneys, the expression of PTGS2 enzyme increases when extracellular fluid volume is reduced, and tachyzoites prompt the expression of PTGS2 in dense spots (71). PTGS2 induces prostaglandins to mitigate the constriction of small glomerular arteries related to filtration in a paracrine manner (72). COX-2 inhibitors may diminish this protective mechanism that sustains glomerular perfusion, leading to prolonged constriction of small glomerular arteries and inadequate renal perfusion. This helps elucidate the connection between PTGS2 and renal function. On one hand, excessive PTGS2 expression exacerbates the inflammatory response, causing harm to the kidneys, while on the other hand, excessive suppression of PTGS2 may elevate the risk of hyperkalemia and renal failure. Therefore, based on the findings of the current study, it is suggested that RAAS inhibitors modifying the level of PTGS2 expression in various tissues of DN kidneys may be associated with acute kidney damage, although the precise mechanism of action remains unclear. The only definite conclusion drawn is that PTGS2 may heighten the risk of acute renal failure when RAAS inhibitors are active in DN conditions.

The impact of diabetes on the gene expression of integrin subunits is widely acknowledged, affecting various cell types and tissues including monocytes, arterial endothelial cells, glomerular cells, and the retina (73). The ITGA4 gene, which codes for α4 integrin, has been demonstrated in recent research to play a role in the PI3K-AKT signaling pathway associated with nephroprotective effects (74). This aligns with our discovery that ITGA4 exhibits a protective effect against acute renal failure in the presence of RAAS inhibitors in DN. ANPEP, also known as aminopeptidase N or CD13, is a multifunctional membrane-bound zinc-dependent metalloprotease that is widely present in renal tissue (75). Current studies did not identify any link between ANPEP and kidney failure. According to our findings, ANPEP serves as a protective factor against acute renal failure in DN patients treated with RAAS inhibitors. This sets the stage for further comprehensive exploration of RAAS inhibitors. In addition, we predicted the TFs, miRNAs and lncRNAs targeting CTSC and PDE5A, and constructed their interaction networks. The construction of ceRNA networks can help us to deeply study the mechanism of gene regulation, reveal the laws of DN occurrence and development, and provide new ideas and strategies for the treatment of DN. However, the mechanism of CTSC and PDE5A in DN needs to be further studied and verified.

## Conclusion

The MR approach utilizes IVs as exposures to examine correlations with outcomes, enhancing the persuasiveness of the results by minimizing confounding factors. In this study, we employed network pharmacology combined with MR to screen the key targets of RAAS inhibitors currently employed in clinical practice for DN. Our findings establish a causal relationship between CTSC, PDE5A, and DN. These findings warrant further investigation into the following three primary aspects. Firstly, previous studies have lacked sufficient evidence establishing a causal relationship between RAB38/CTSC and human urinary protein. However, our study observed a causative link between CTSC and DN, considering the notable protein-lowering ability of RAAS inhibitors in the clinical setting. It is reasonable to hypothesize that CTSC, as the core target of RAAS inhibitors, plays a role in human urinary protein. Deeper studies are necessary to explore the specific mechanisms and potential involvement of RAB38. Secondly, the impact of RAAS inhibitors on PDE5A has been found to be causally associated with DN. Several studies have concluded that current PDE5A inhibitors possess renoprotective effects and are linked to altered renal hemodynamics, with excessive dilation or inhibition having detrimental renal effects. The role of current medications in regulating the balance of PDE5A in different kidney tissues remains unknown. Further studies focused on this target may uncover additional clinical benefits. Thirdly, CTSC and PDE5A contribute to the diagnosis of DN. Thus, there is an opportunity to combine CTSC and PDE5A screening with urine protein, urine protein/creatinine ratio, 24-hour urinary protein quantitation, and urinary protein excretion rate to predict the risk of DN in diabetic patients. The finding that PTGS2, ITGA4, and ANPEP may control acute kidney failure in the presence of RAAS inhibitors in DN is supported by the fact that all three are impacted by RAAS inhibitors and causal analysis using drug-target Mendelian randomization has demonstrated their link to acute kidney failure. Specifically, PTGS2 is identified as a risk factor, while the other two are regarded as protective factors. This discovery provides new evidence for the potential reduction of kidney damage with concurrent clinical use of RAAS inhibitors in treating DN.

## Limitation

In this study we used strict analytical criteria (e.g., P < 5 × 10^-8^, r2 < 0.001, kb = 10000) in the drug-target Mendelian analysis considering the accuracy and rigor of the results. Thus there may be other RAAS inhibitor-related targets that have a causal relationship with DN yet to be discovered. Due to sample limitations, stratification by disease type and patient gender was not performed, which resulted in certain biases. Vasopressin is associated with the activation of the RAAS system. In our study, we have not yet included vasopressin in the analysis, but we will further explore this direction in the future. Clinical applications of key targets need to be supported by data from more samples. However, we will continue to focus on the role of these key targets.

## Data availability statement

The datasets presented in this study can be found in online repositories. The names of the repository/repositories and accession number(s) can be found in the article/**Supplementary Material**.

## Author contributions

DZ: Writing – original draft, Writing – review & editing. TZ: Methodology, Software, Writing – review & editing. ST: Writing – review & editing. QL: Writing – review & editing. WL: Writing – review & editing. GG: Software, Writing – review & editing. ML: Writing – review & editing. QC: Writing – original draft, Writing – review & editing.
