# Supplementary material for: Network pharmacology combined with Mendelian randomization analysis to identify the key targets of renin-angiotensin-aldosterone system inhibitors in the treatment of diabetic nephropathy
Source: Front Endocrinol (Lausanne). 2024 Jan 25;15:1354950. doi: 10.3389/fendo.2024.1354950 (PMC10850565; doi:10.3389/fendo.2024.1354950)
Supplement: Supplementary file 1 [file DataSheet_1.zip › ╝▒╨╘╔÷╦Ñ╜▀┼·┴┐╖╓╬÷/01PTGS2/0801.Scatter.pdf]

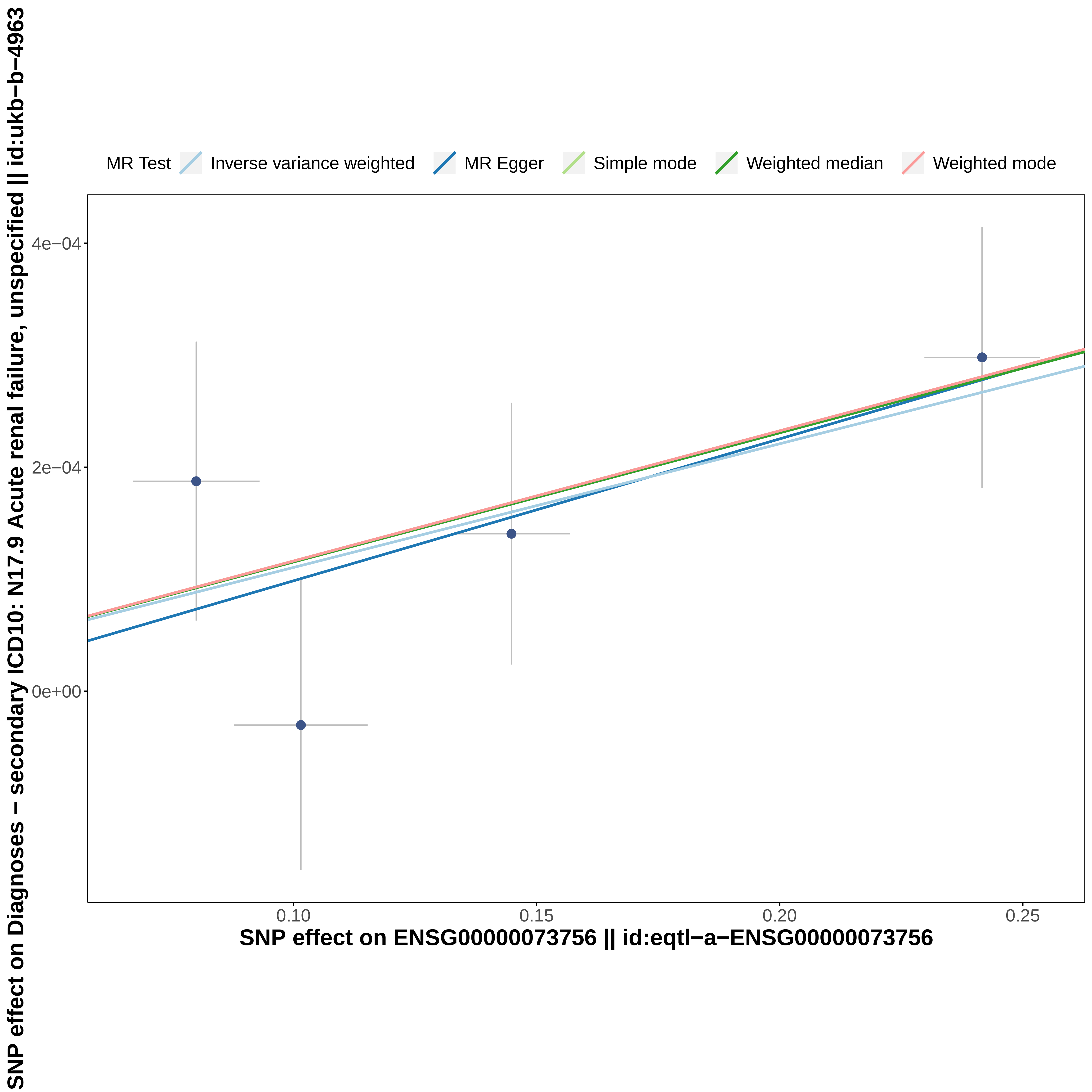

MR Test
Inverse variance weighted
MR Egger
Simple mode
Weighted median
Weighted mode
4e−04
2e−04
0e+00
0.10
0.15
0.20
0.25
SNP effect on ENSG00000073756 || id:eqtl−a−ENSG00000073756
SNP effect on Diagnoses − secondary ICD10: N17.9 Acute renal failure, unspecified || id:ukb−b−4963
