# Supplementary material for: Network pharmacology combined with Mendelian randomization analysis to identify the key targets of renin-angiotensin-aldosterone system inhibitors in the treatment of diabetic nephropathy
Source: Front Endocrinol (Lausanne). 2024 Jan 25;15:1354950. doi: 10.3389/fendo.2024.1354950 (PMC10850565; doi:10.3389/fendo.2024.1354950)
Supplement: Supplementary file 1 [file DataSheet_1.zip › ╝▒╨╘╔÷╦Ñ╜▀┼·┴┐╖╓╬÷/02ITGA4/07.leave_one_out.pdf]

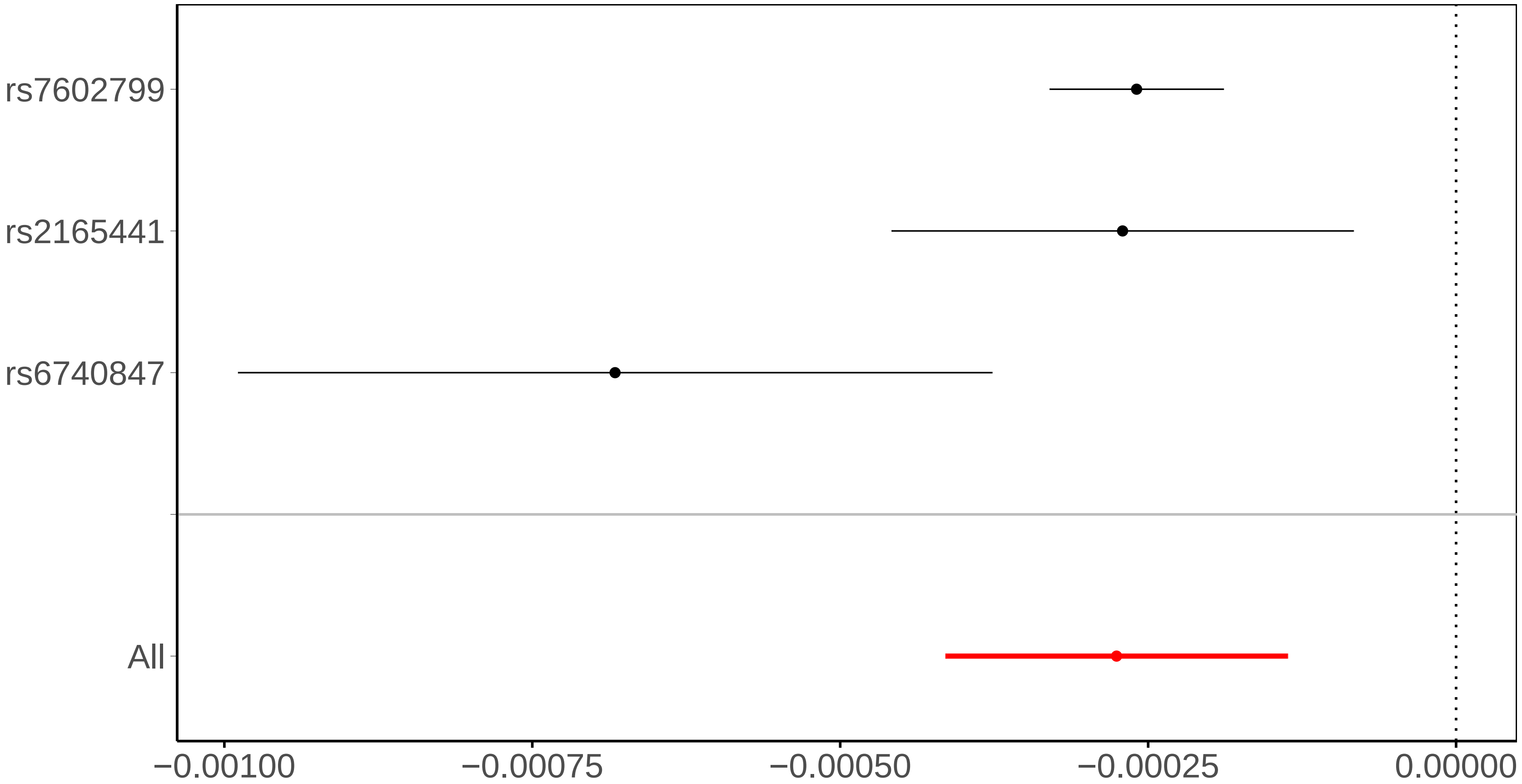

MR leave−one−out sensitivity analysis for
'ENSG00000115232 || id:eqtl−a−ENSG00000115232' on 'Diagnoses − secondary ICD10: N17.9 Acute renal failure, unspecified || id:ukb−b−4963'
