# Supplementary figures and images for: Network pharmacology combined with Mendelian randomization analysis to identify the key targets of renin-angiotensin-aldosterone system inhibitors in the treatment of diabetic nephropathy
Source: Front Endocrinol (Lausanne). 2024 Jan 25;15:1354950. doi: 10.3389/fendo.2024.1354950 (PMC10850565; doi:10.3389/fendo.2024.1354950)

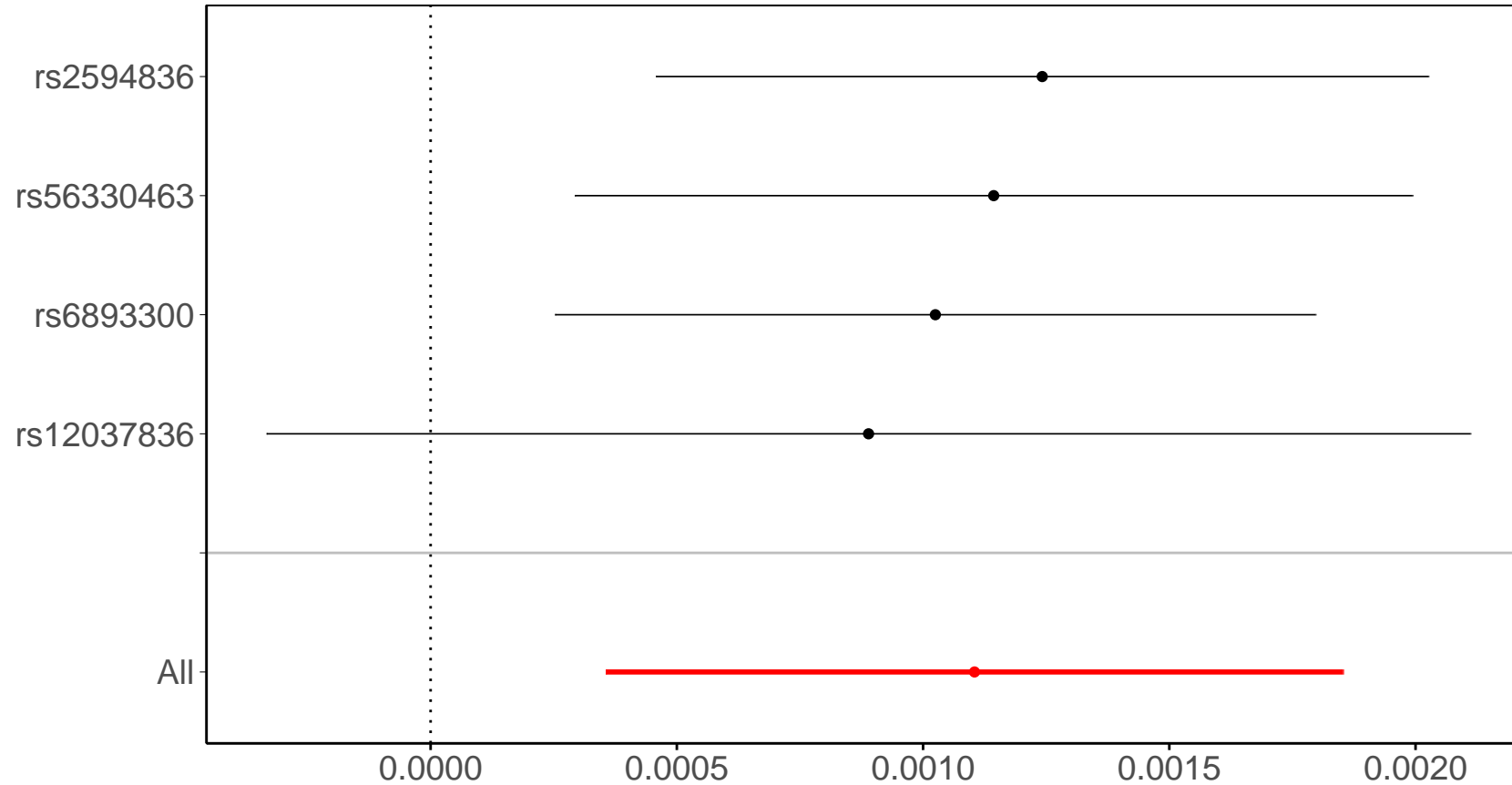

Supplement: Supplementary file 1 [file DataSheet_1.zip › ╝▒╨╘╔÷╦Ñ╜▀┼·┴┐╖╓╬÷/01PTGS2/07.leave_one_out.pdf]

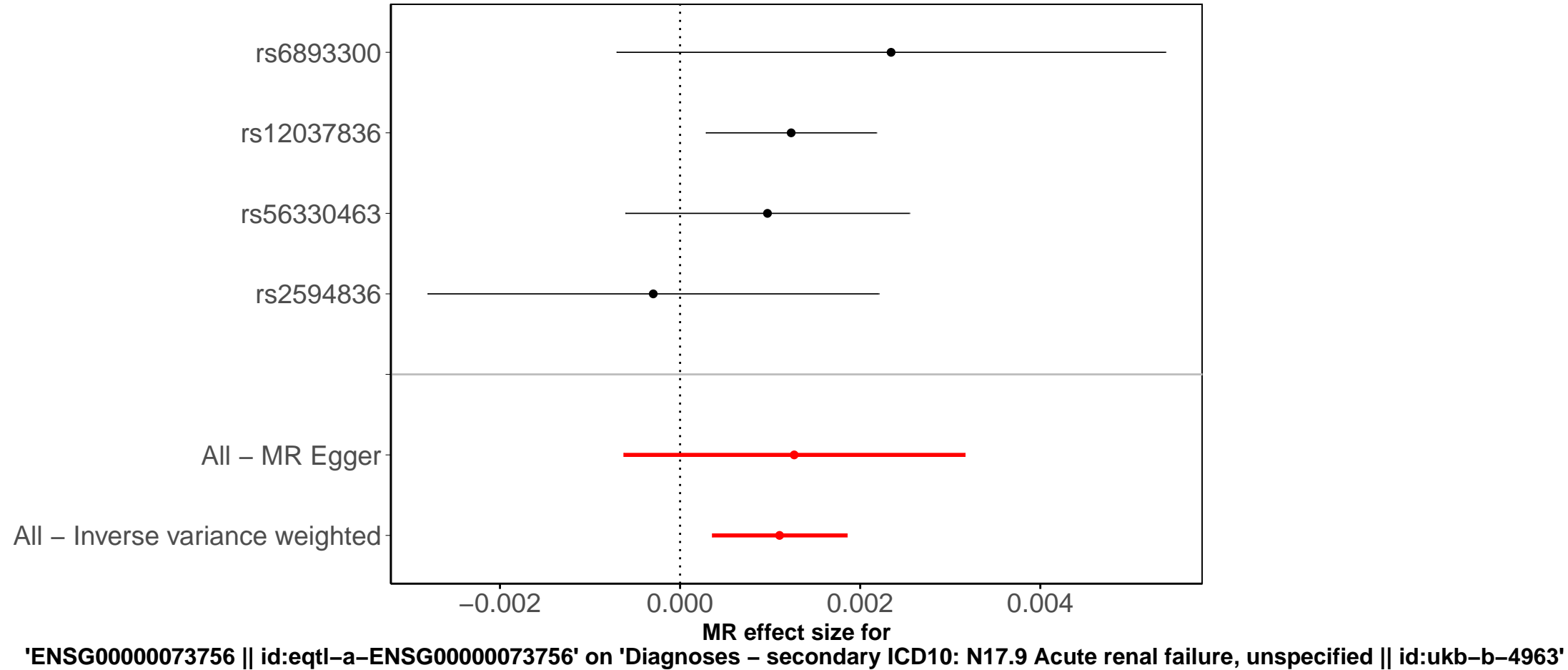

Supplement: Supplementary file 1 [file DataSheet_1.zip › ╝▒╨╘╔÷╦Ñ╜▀┼·┴┐╖╓╬÷/01PTGS2/10.Forest_Plot.pdf]

MR Method

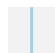

Inverse variance weighted

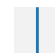

MR Egger

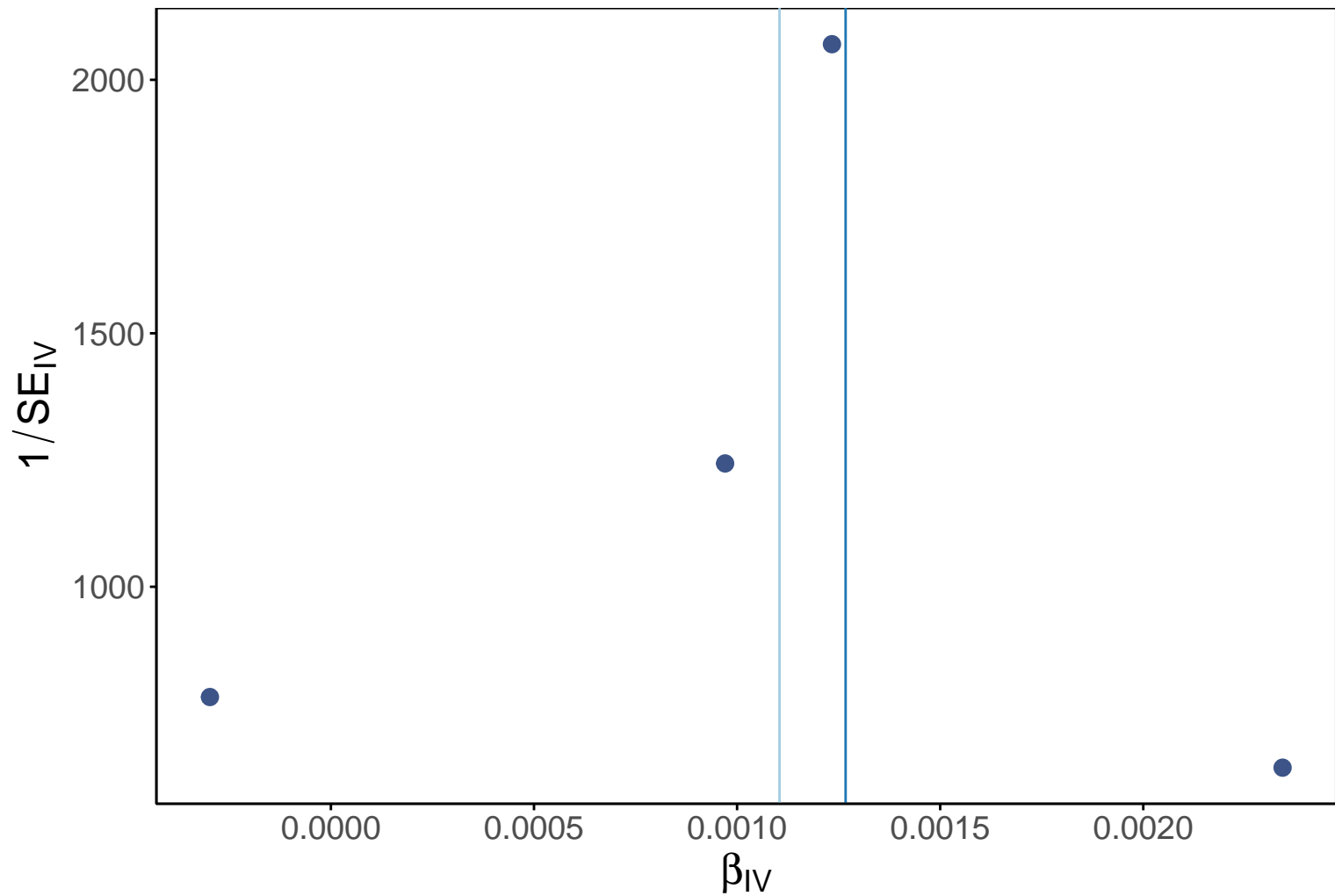

Supplement: Supplementary file 1 [file DataSheet_1.zip › ╝▒╨╘╔÷╦Ñ╜▀┼·┴┐╖╓╬÷/01PTGS2/11.Funnel.pdf]

SNP effect on Diagnoses – secondary ICD10: N17.9 Acute renal failure, unspecified | :ukb-b-4963

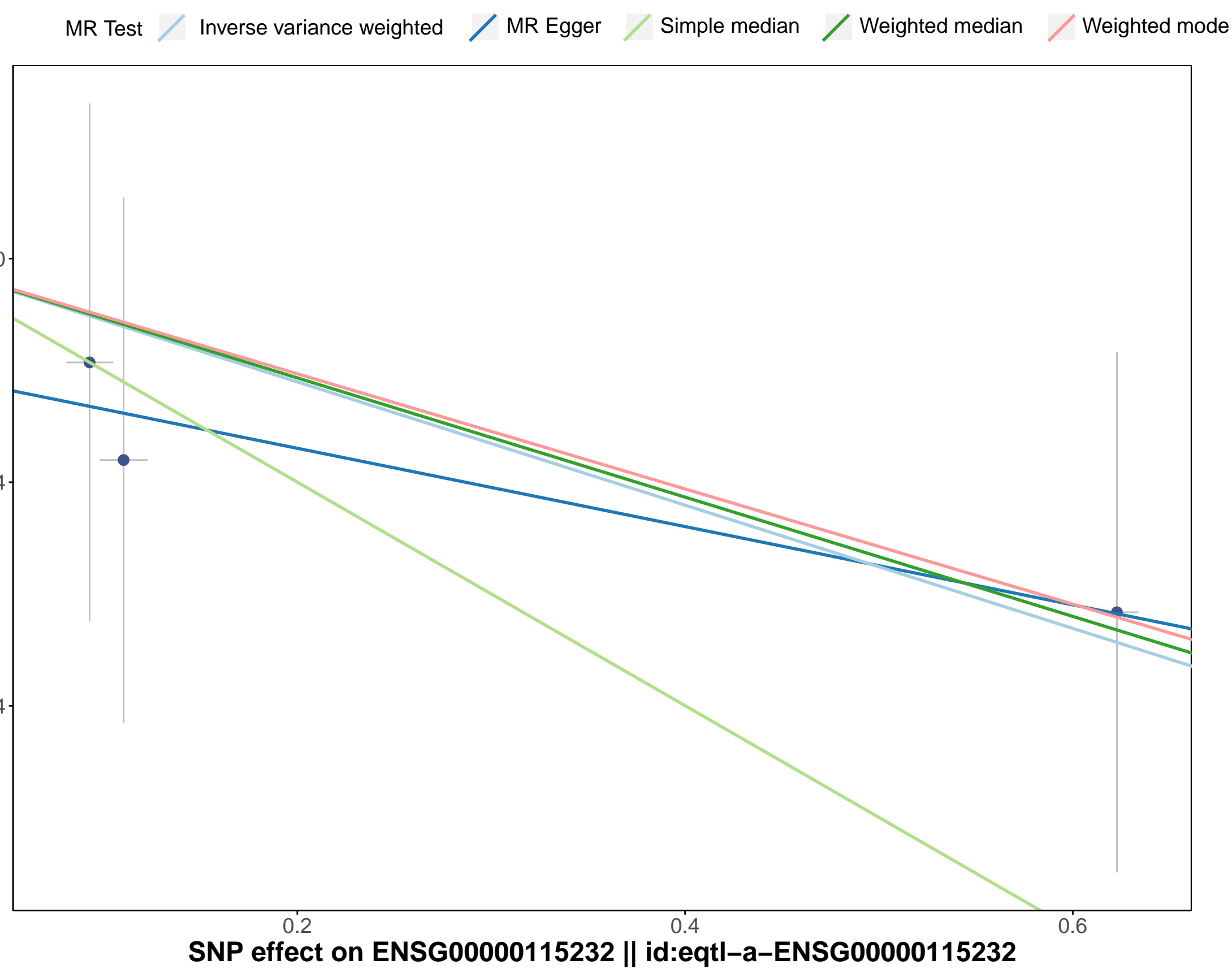

Supplement: Supplementary file 1 [file DataSheet_1.zip › ╝▒╨╘╔÷╦Ñ╜▀┼·┴┐╖╓╬÷/02ITGA4/0801.Scatter.pdf]

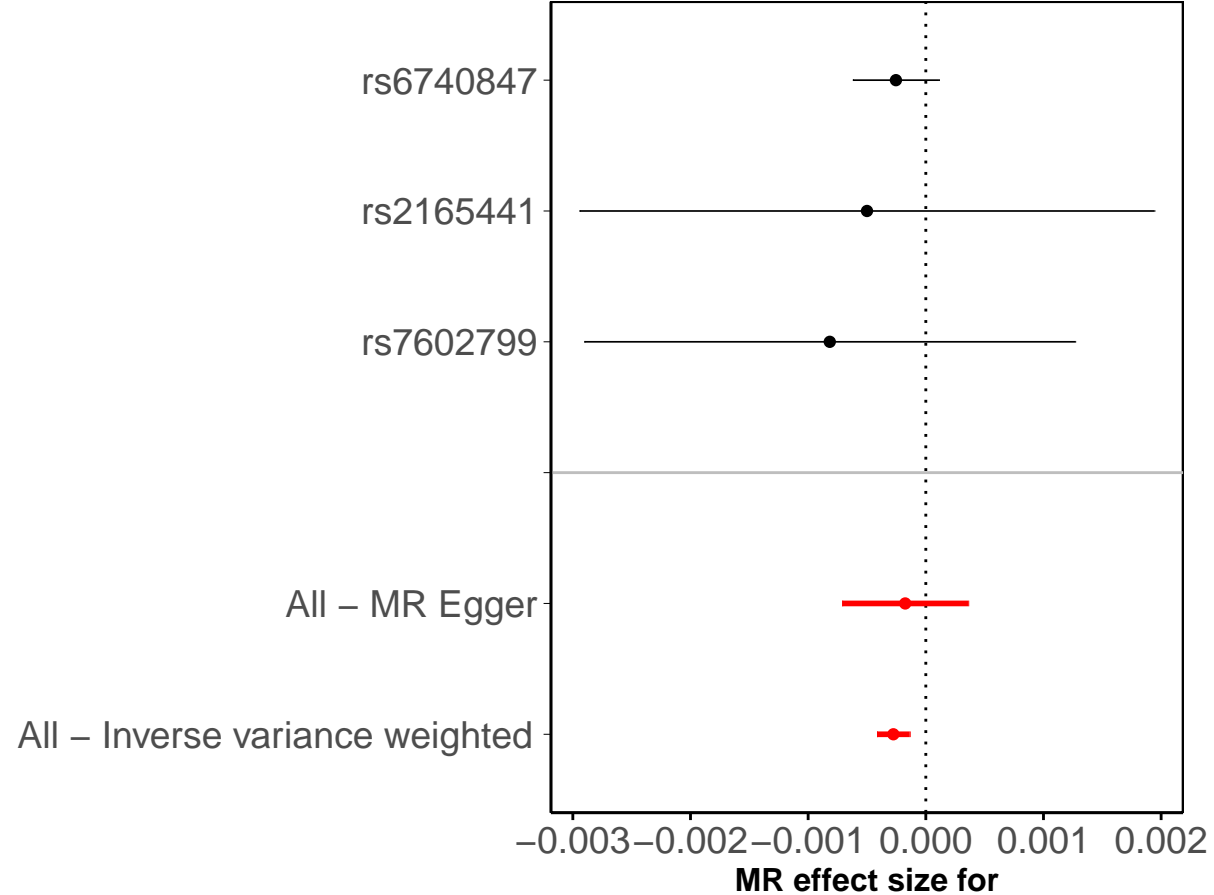

'ENSG00000115232 || id:eqtl-a-ENSG00000115232' on 'Diagnoses - secondary ICD10: N17.9 Acute renal failure, unspecified || id:ukb-b-4963'

Supplement: Supplementary file 1 [file DataSheet_1.zip › ╝▒╨╘╔÷╦Ñ╜▀┼·┴┐╖╓╬÷/02ITGA4/10.Forest_Plot.pdf]

MR Method

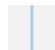

Inverse variance weighted (multiplicative random effects)

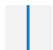

MR Egger

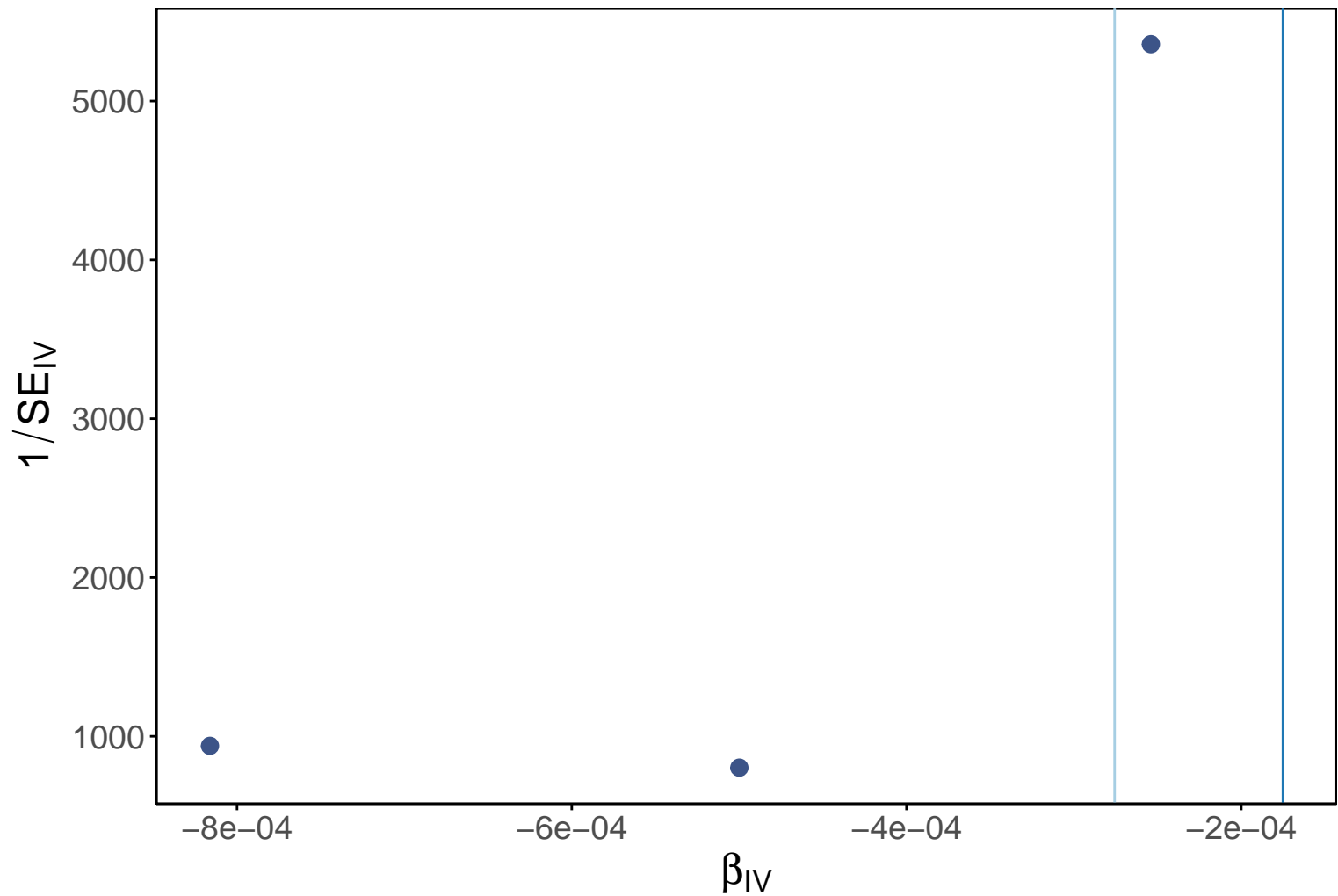

Supplement: Supplementary file 1 [file DataSheet_1.zip › ╝▒╨╘╔÷╦Ñ╜▀┼·┴┐╖╓╬÷/02ITGA4/11.Funnel.pdf]

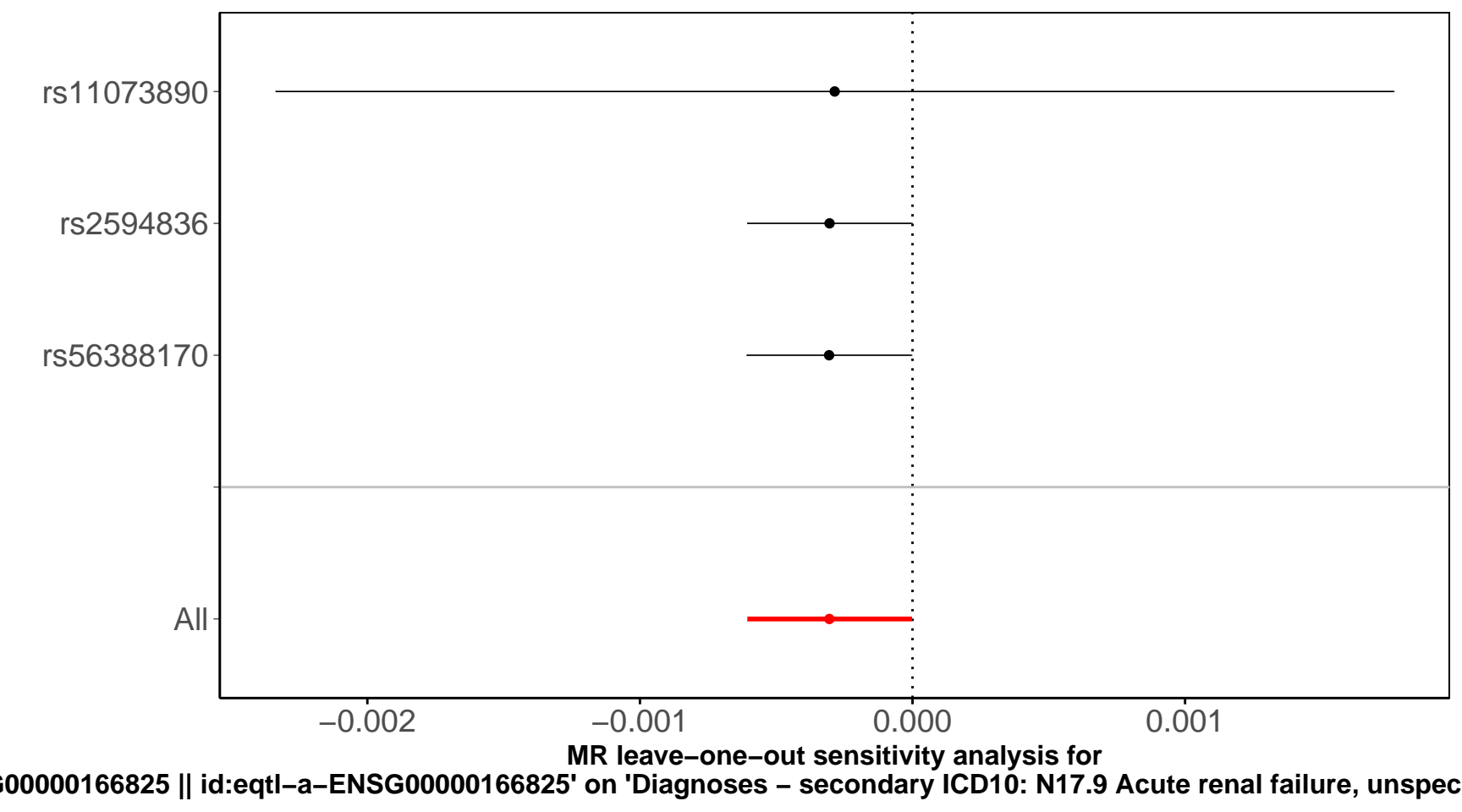

Supplement: Supplementary file 1 [file DataSheet_1.zip › ╝▒╨╘╔÷╦Ñ╜▀┼·┴┐╖╓╬÷/03ANPEP/07.leave_one_out.pdf]

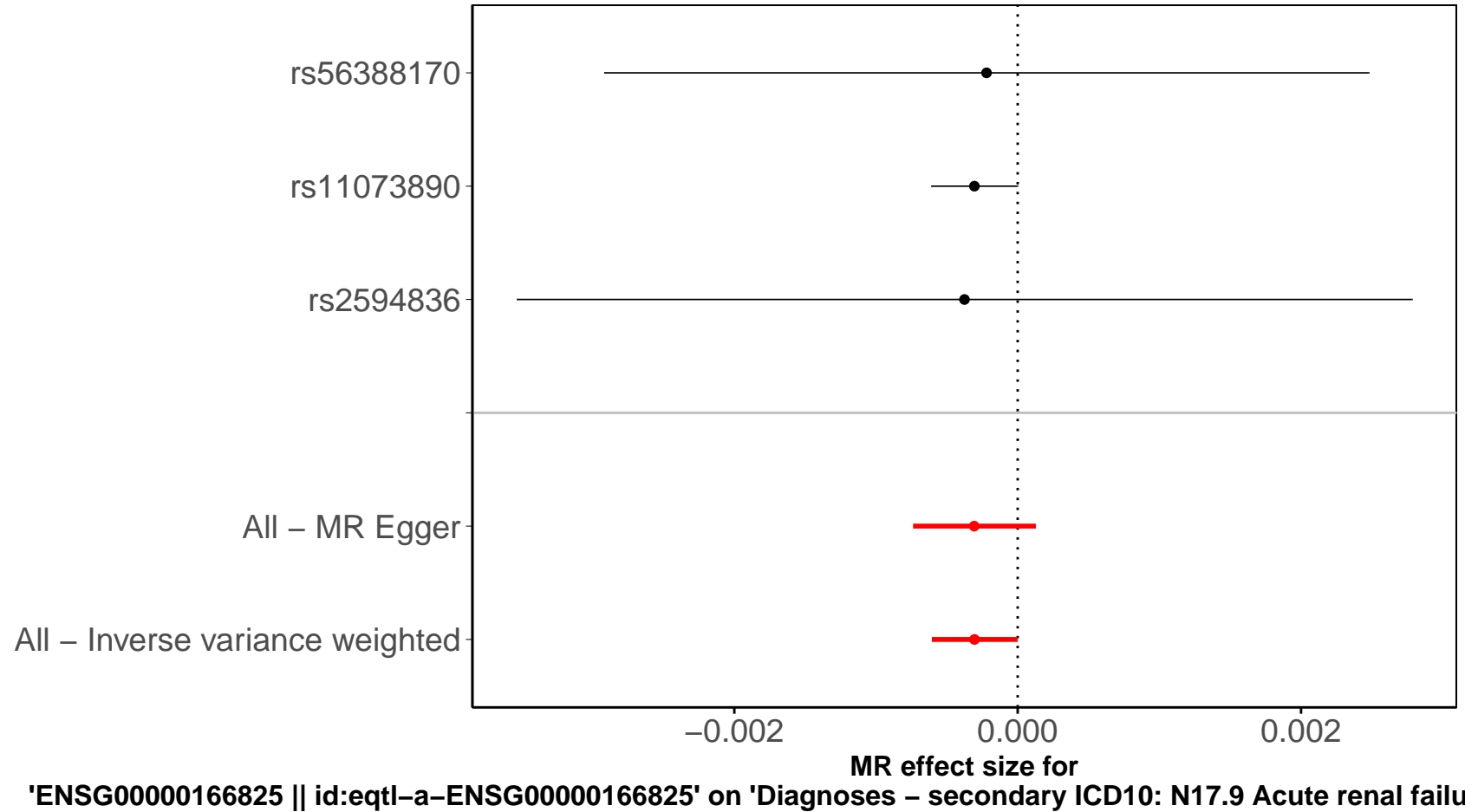

Supplement: Supplementary file 1 [file DataSheet_1.zip › ╝▒╨╘╔÷╦Ñ╜▀┼·┴┐╖╓╬÷/03ANPEP/10.Forest_Plot.pdf]

MR Method

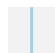

Inverse variance weighted

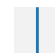

MR Egger

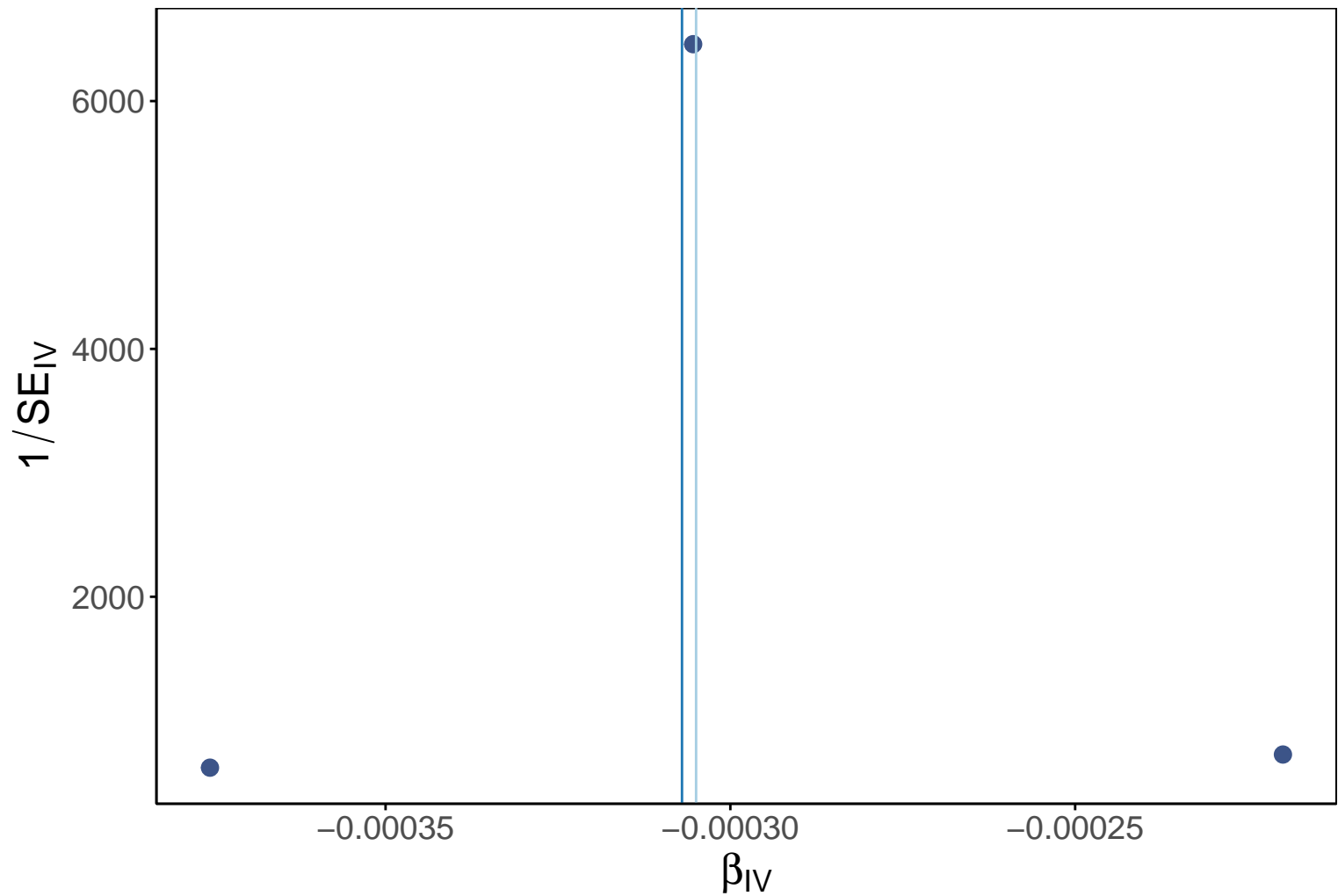

Supplement: Supplementary file 1 [file DataSheet_1.zip › ╝▒╨╘╔÷╦Ñ╜▀┼·┴┐╖╓╬÷/03ANPEP/11.Funnel.pdf]

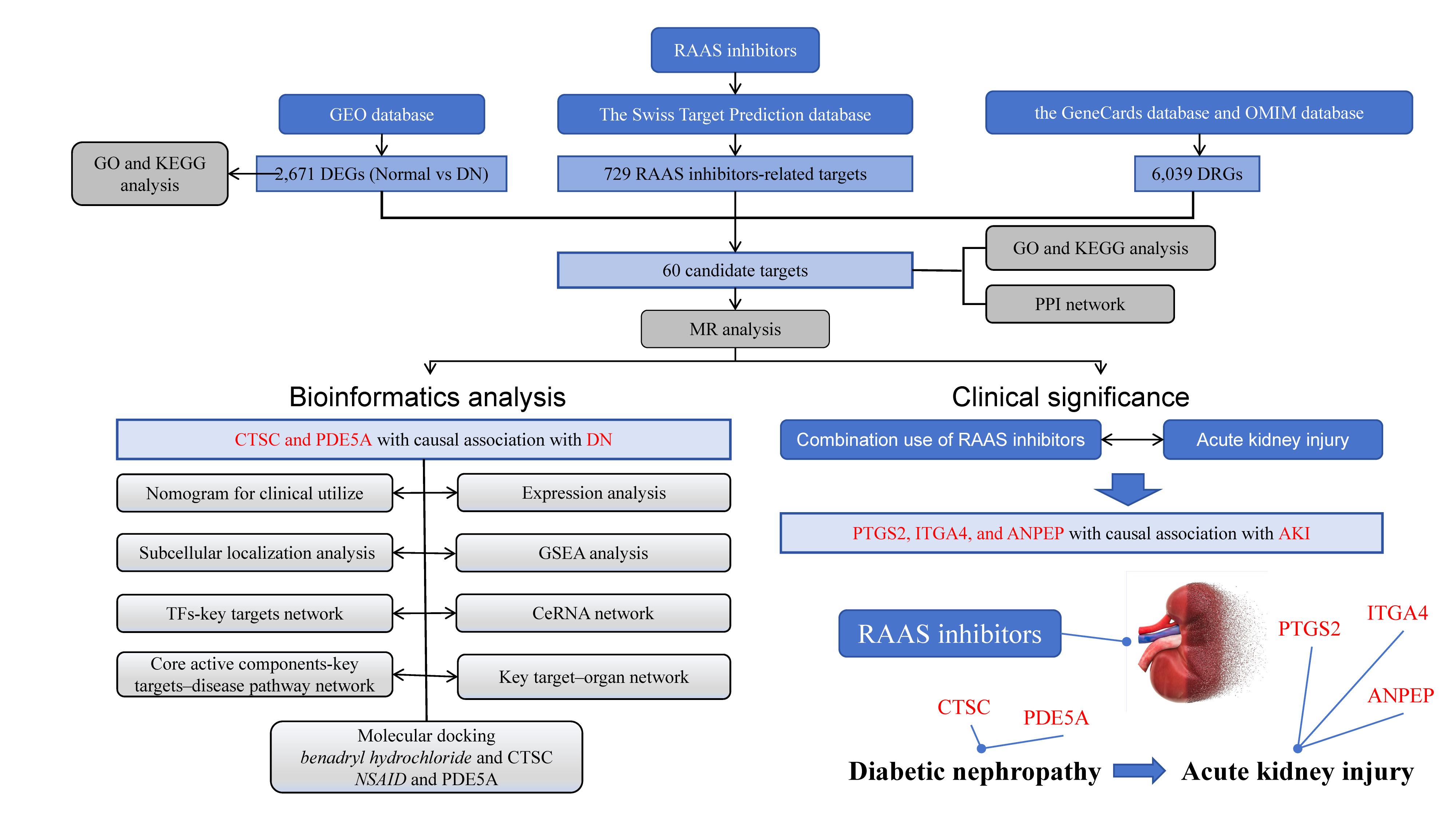

Supplement: Supplementary file 2 [file DataSheet_2.zip › 1. Figure/Figure 1/fig1.tif]

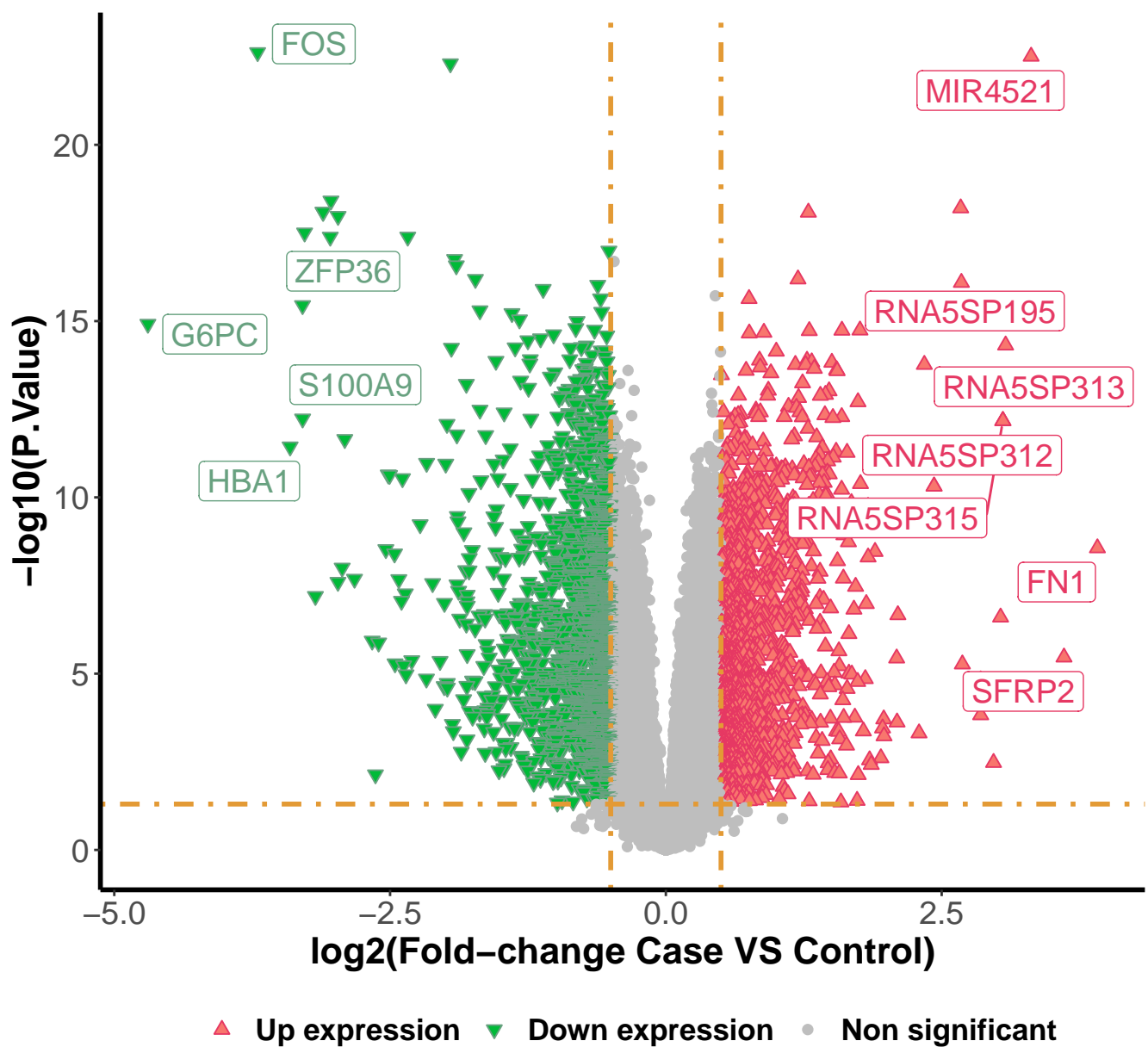

Supplement: Supplementary file 2 [file DataSheet_2.zip › 1. Figure/Figure 2/Figure 2A.pdf]

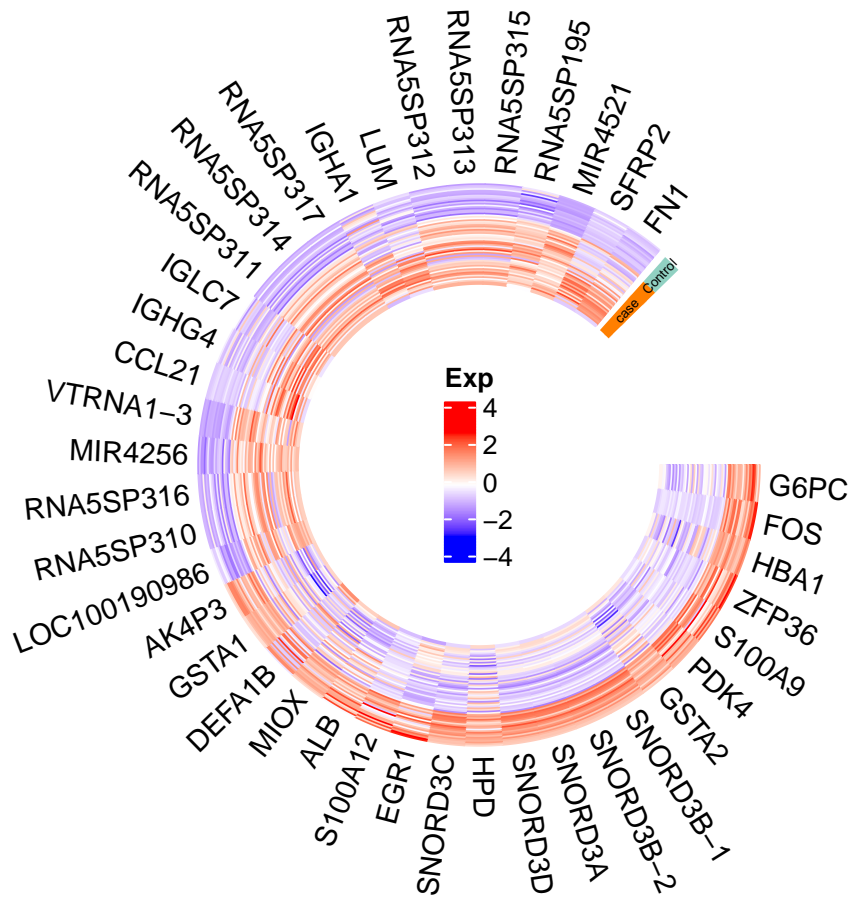

Supplement: Supplementary file 2 [file DataSheet_2.zip › 1. Figure/Figure 2/Figure 2B.pdf]

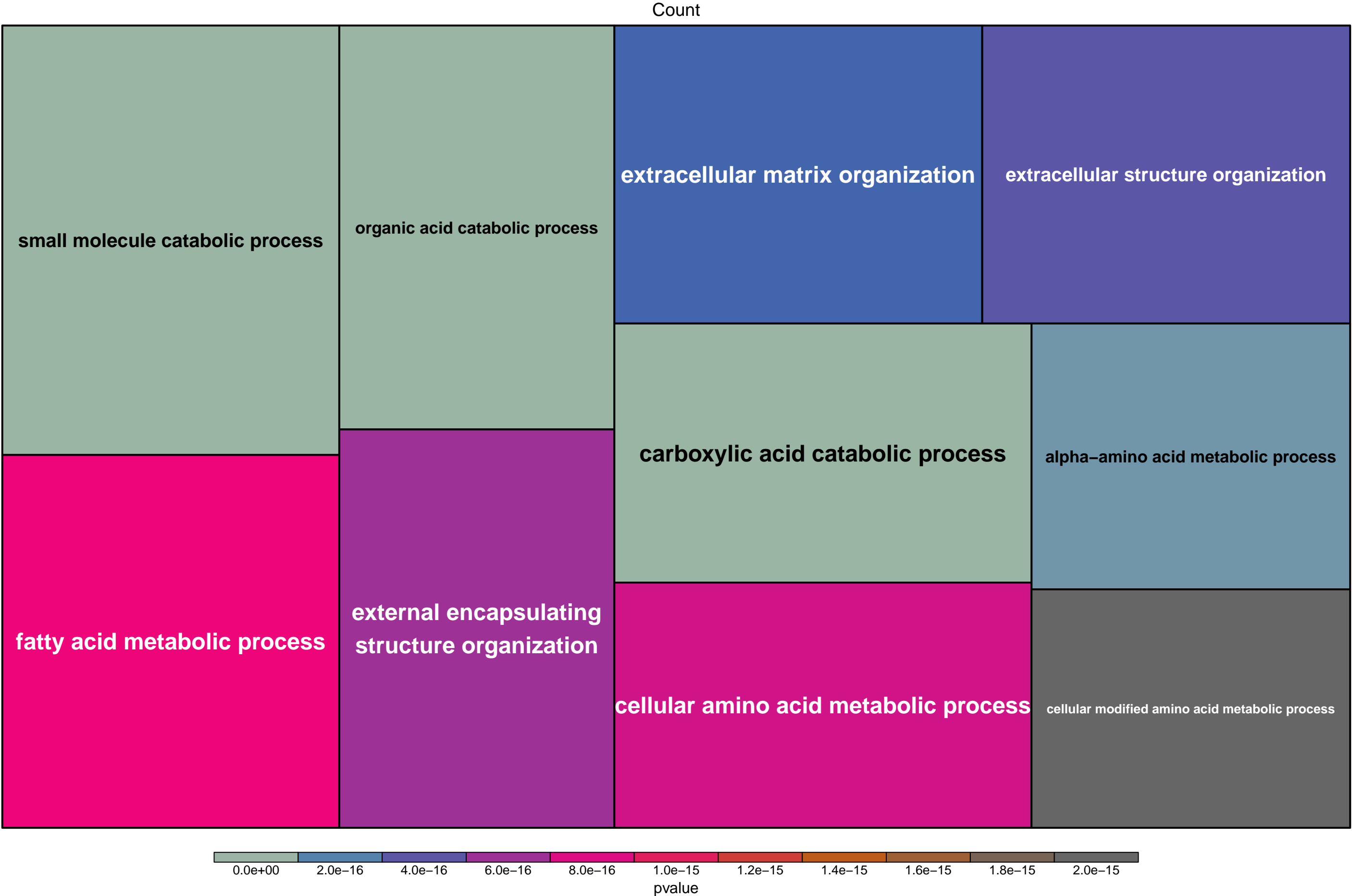

Supplement: Supplementary file 2 [file DataSheet_2.zip › 1. Figure/Figure 2/Figure 2C.pdf]

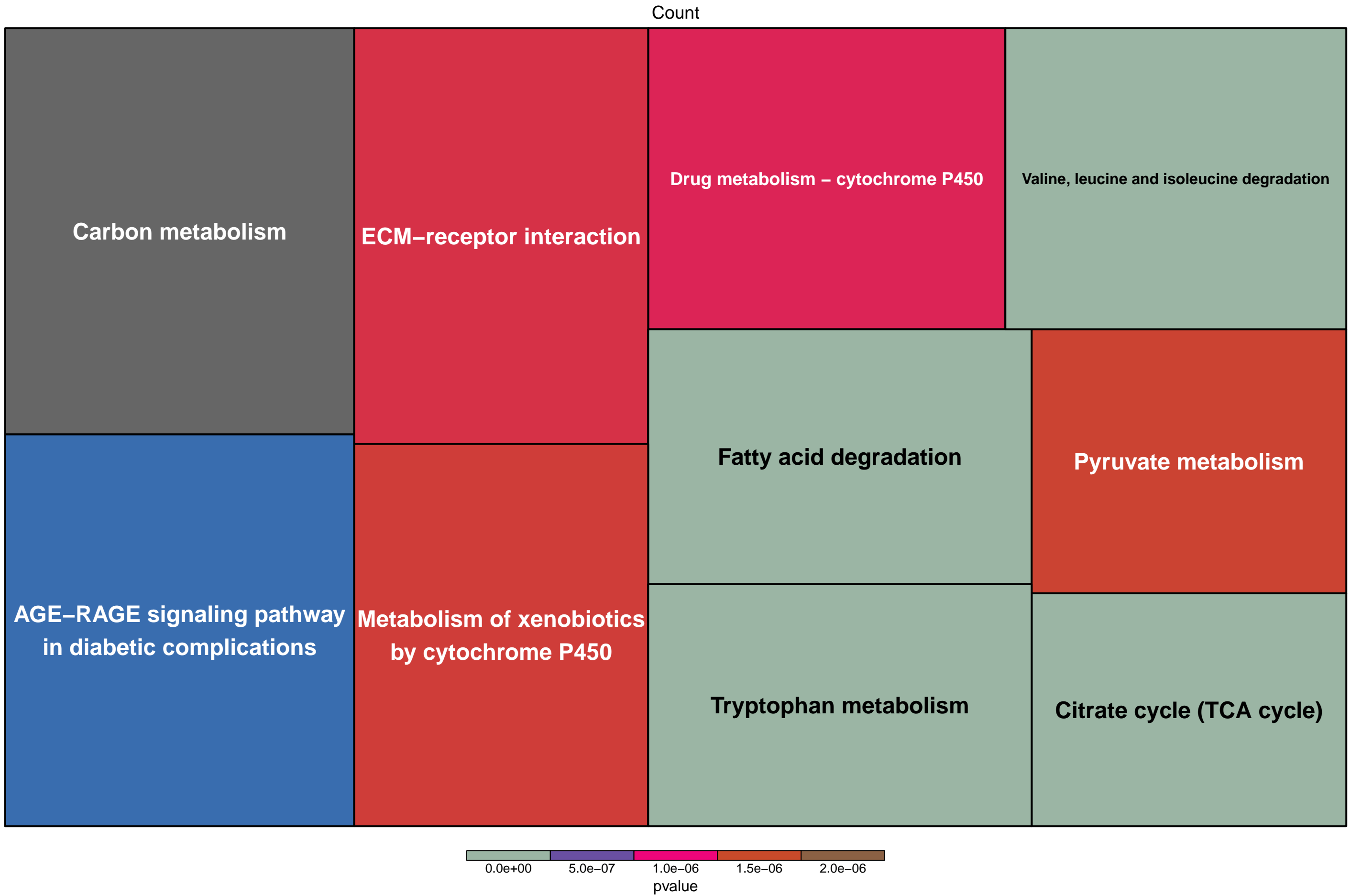

Supplement: Supplementary file 2 [file DataSheet_2.zip › 1. Figure/Figure 2/Figure 2D.pdf]

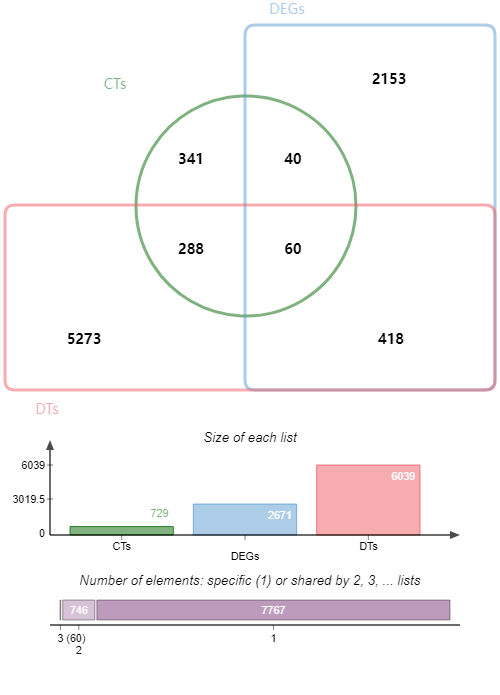

Supplement: Supplementary file 2 [file DataSheet_2.zip › 1. Figure/Figure 3/Figure 3A.png]

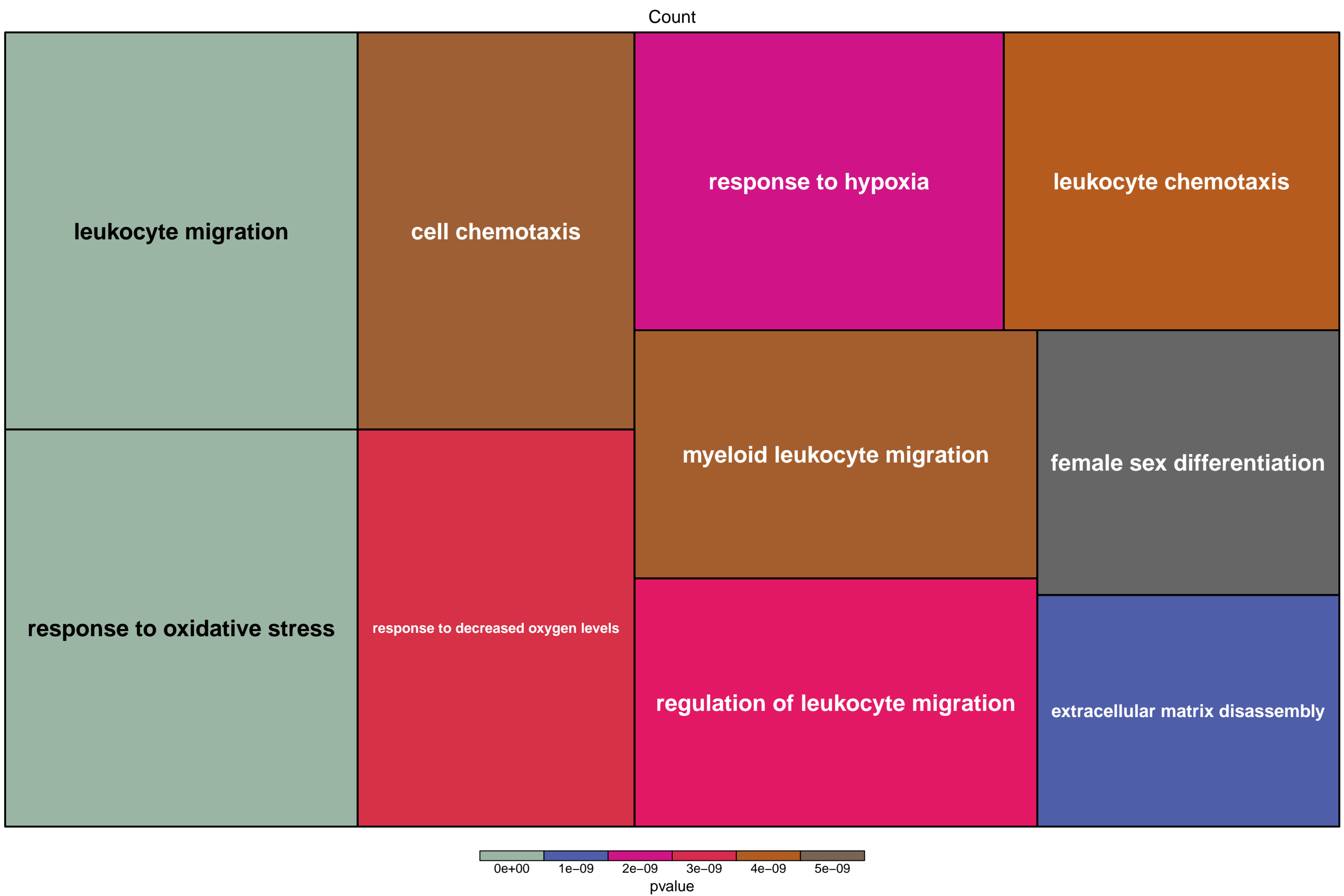

Supplement: Supplementary file 2 [file DataSheet_2.zip › 1. Figure/Figure 3/Figure 3B.pdf]

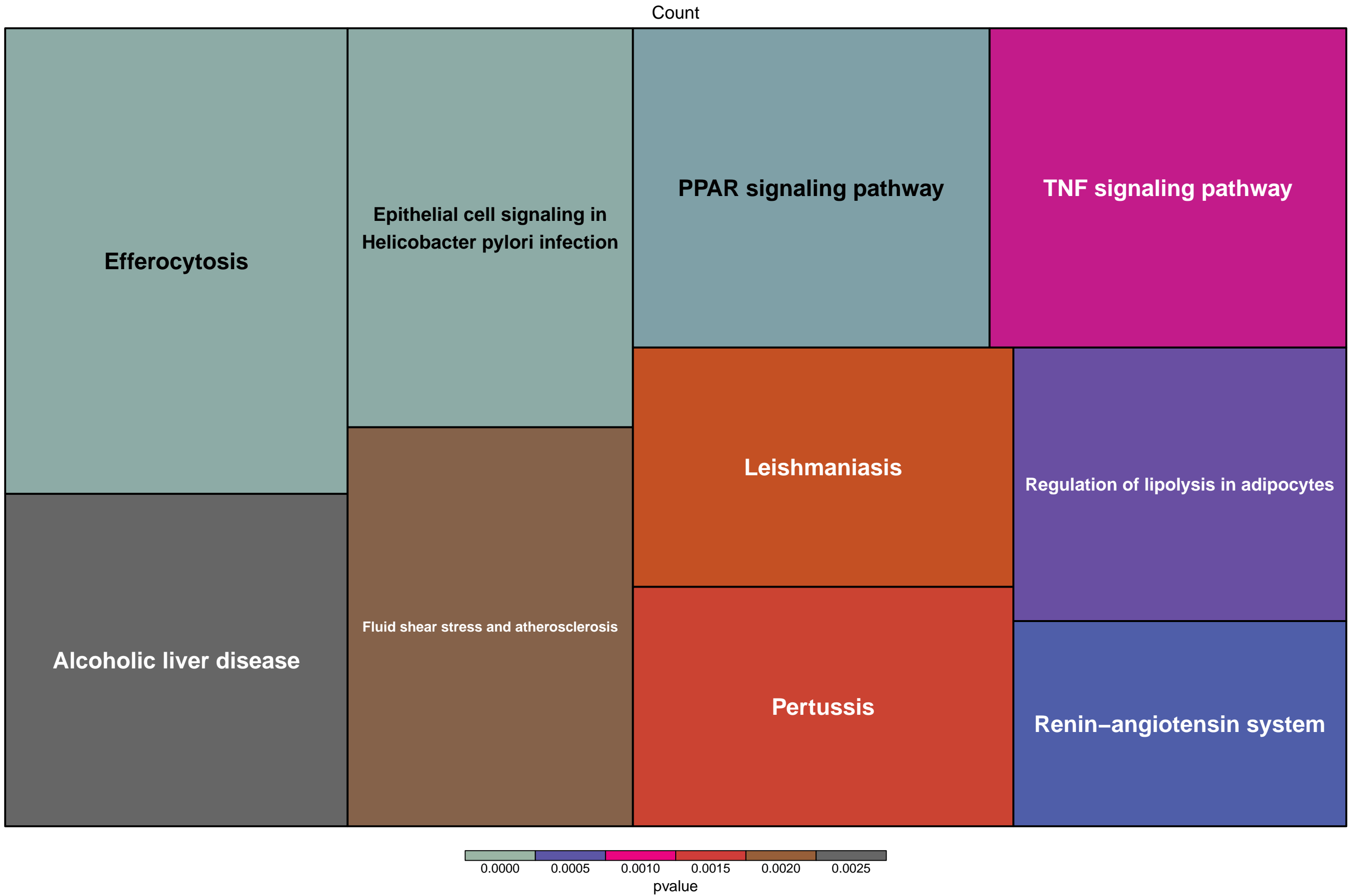

Supplement: Supplementary file 2 [file DataSheet_2.zip › 1. Figure/Figure 3/Figure 3C.pdf]

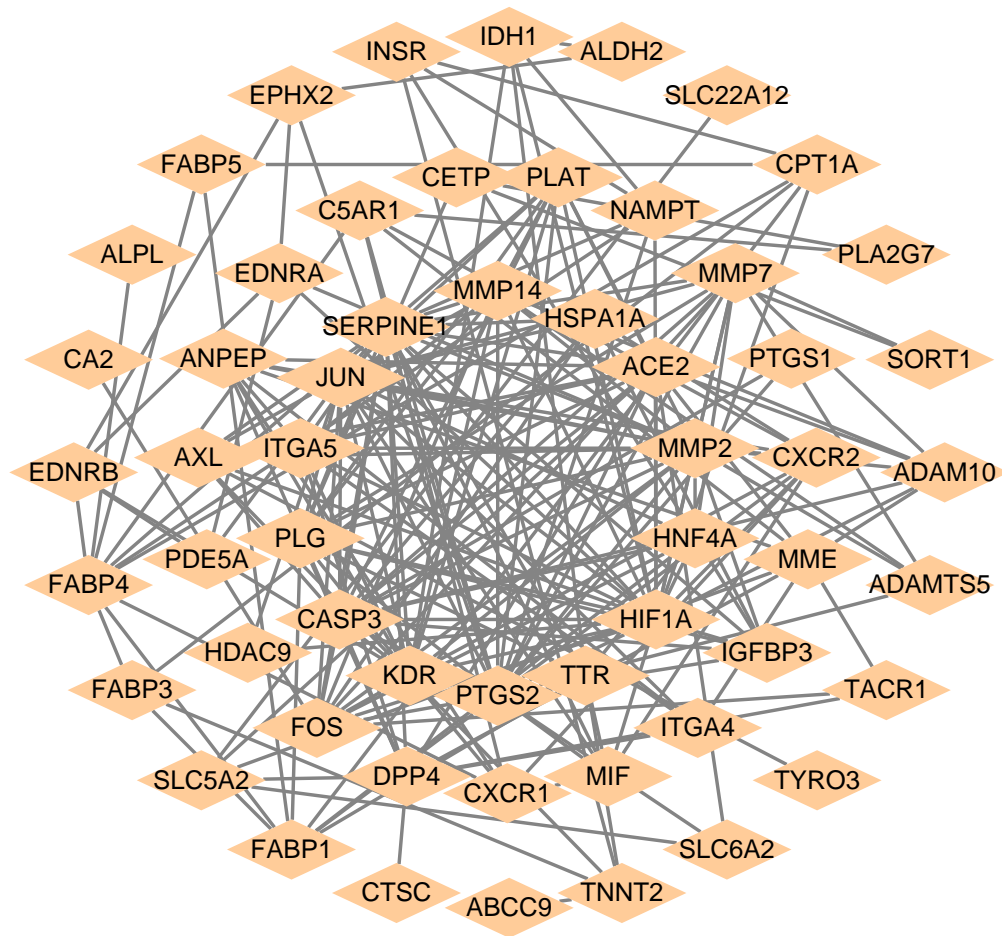

Supplement: Supplementary file 2 [file DataSheet_2.zip › 1. Figure/Figure 3/Figure 3D.pdf]

Case Control

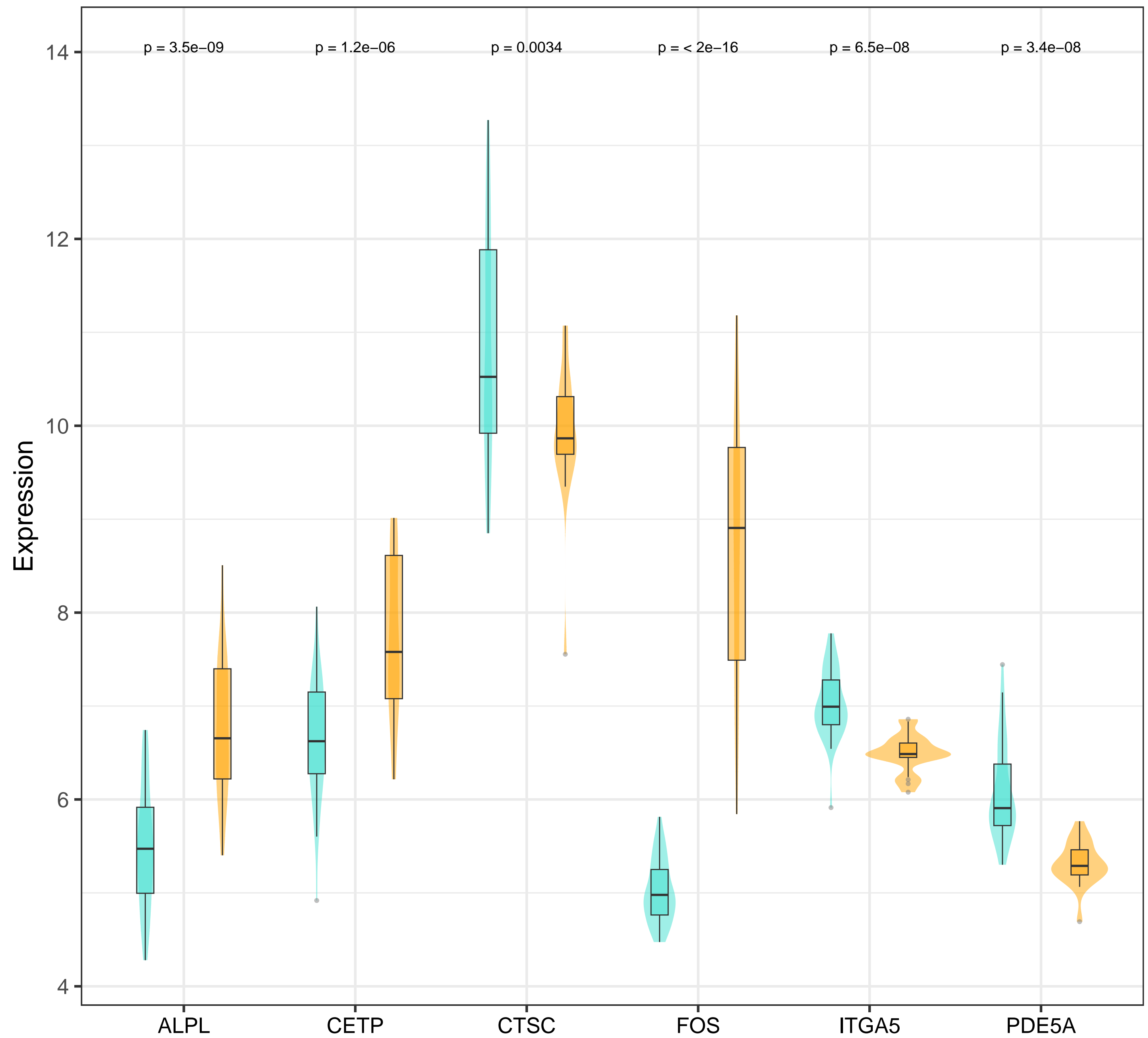

Supplement: Supplementary file 2 [file DataSheet_2.zip › 1. Figure/Figure 4/Figure 4A/╤╡┴╖╝»╨í╠ß╟┘═╝.pdf]

Case Control

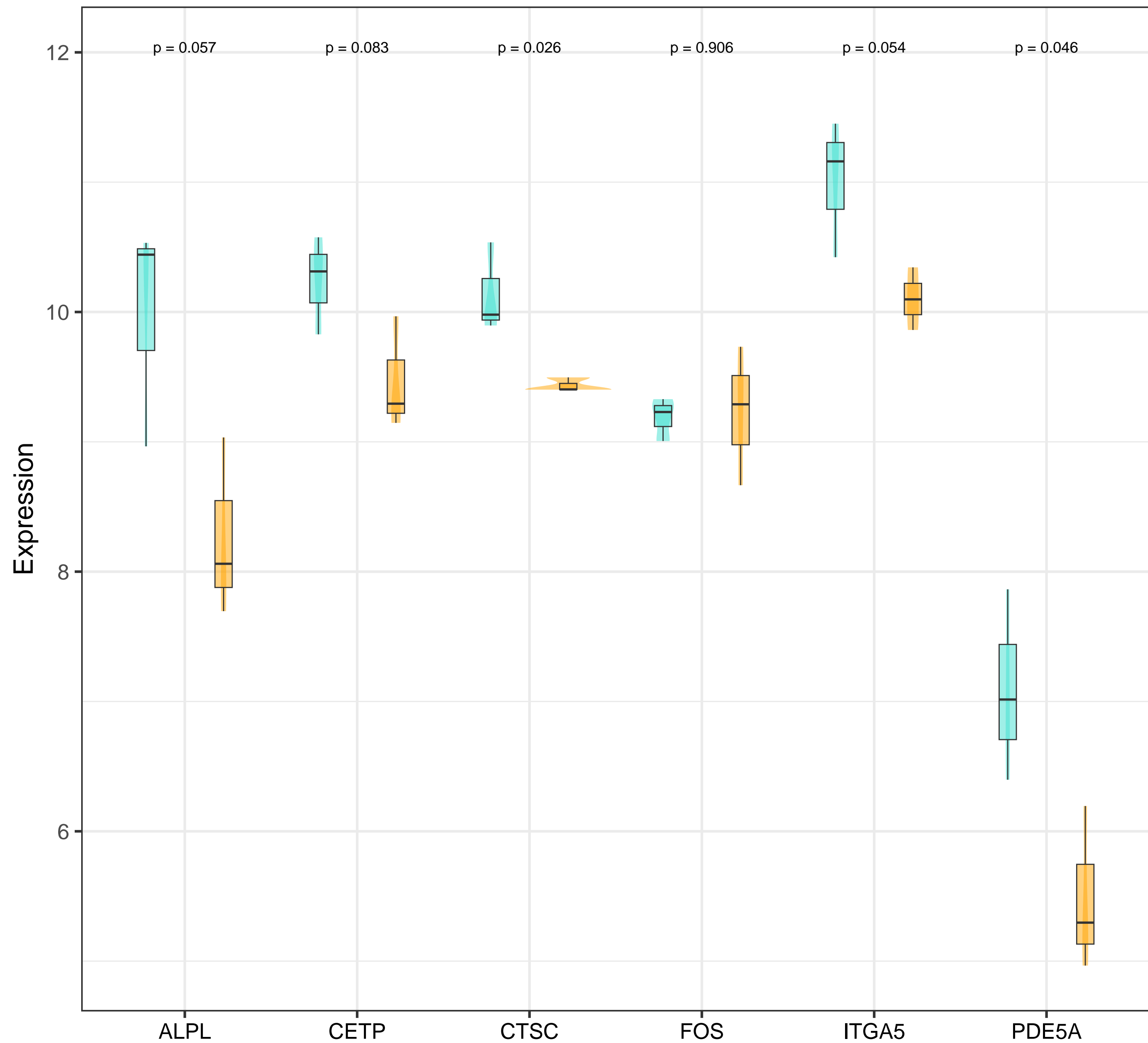

Supplement: Supplementary file 2 [file DataSheet_2.zip › 1. Figure/Figure 4/Figure 4A/╤Θ╓ñ╝»╨í╠ß╟┘═╝.pdf]

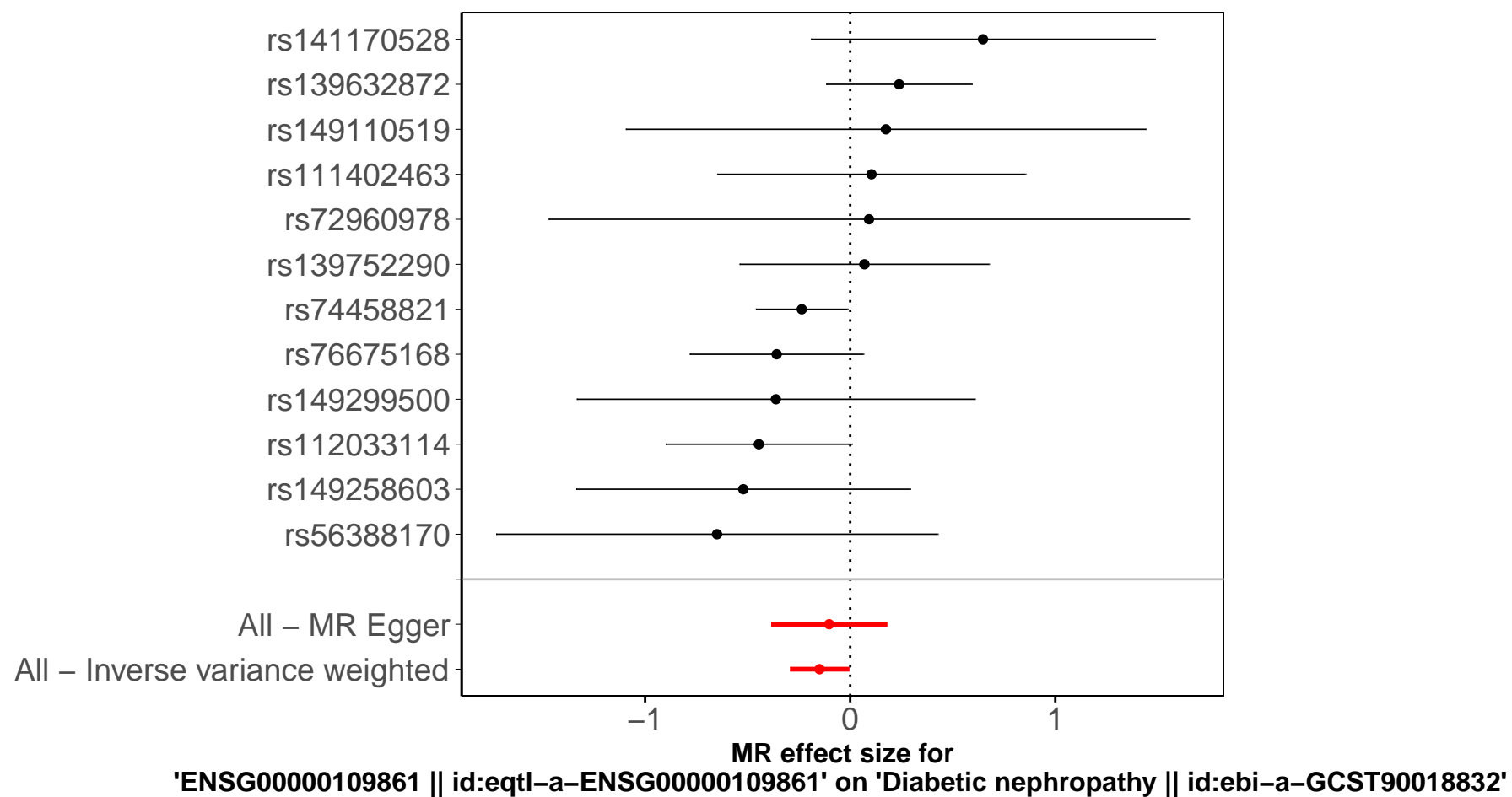

Supplement: Supplementary file 2 [file DataSheet_2.zip › 1. Figure/Figure 4/Figure 4D.pdf]

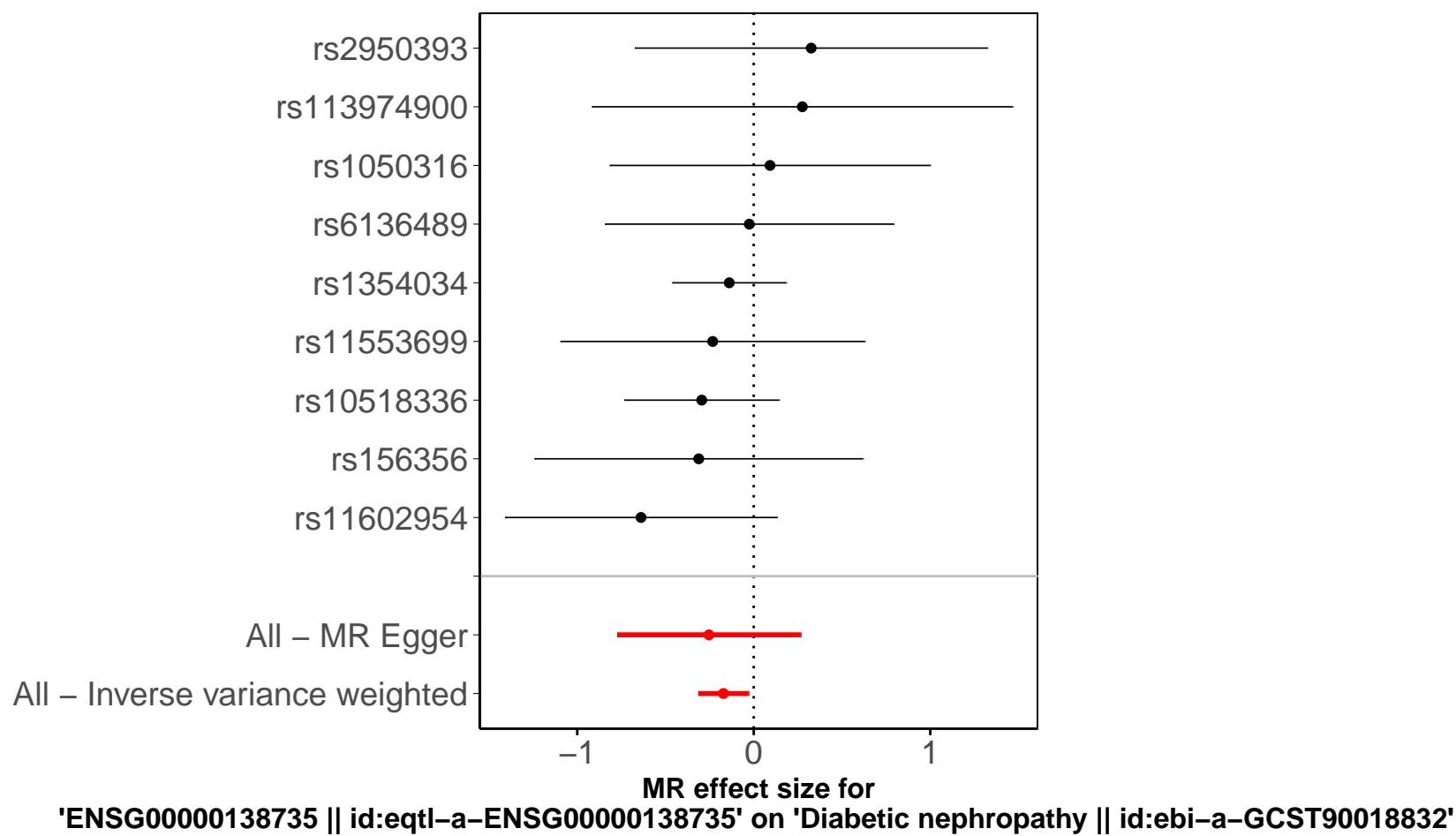

Supplement: Supplementary file 2 [file DataSheet_2.zip › 1. Figure/Figure 4/Figure 4E.pdf]

MR Method    Inverse variance weighted (fixed effects)    MR Egger

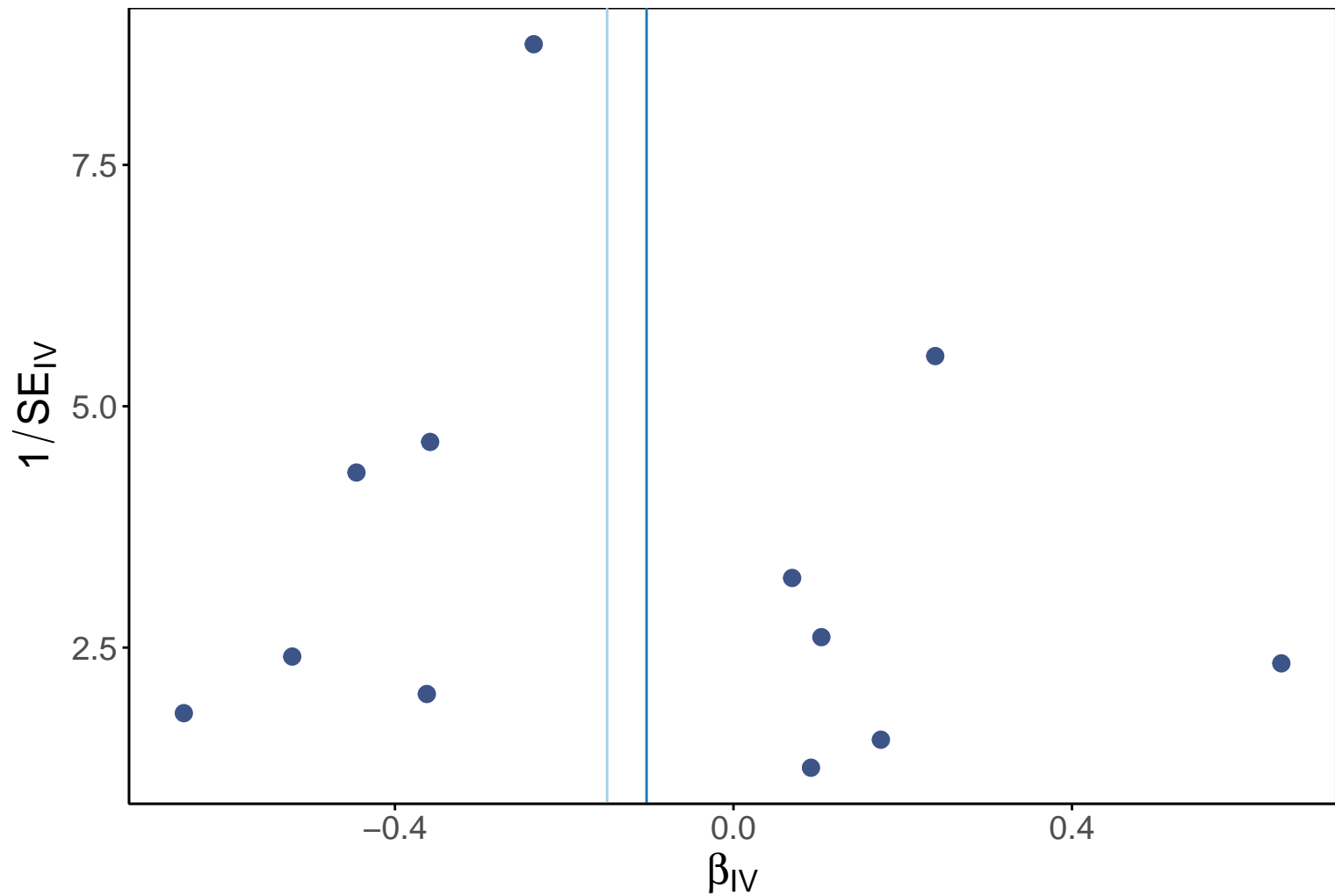

Supplement: Supplementary file 2 [file DataSheet_2.zip › 1. Figure/Figure 4/Figure 4F.pdf]

MR Method    Inverse variance weighted (multiplicative random effects)    MR Egger

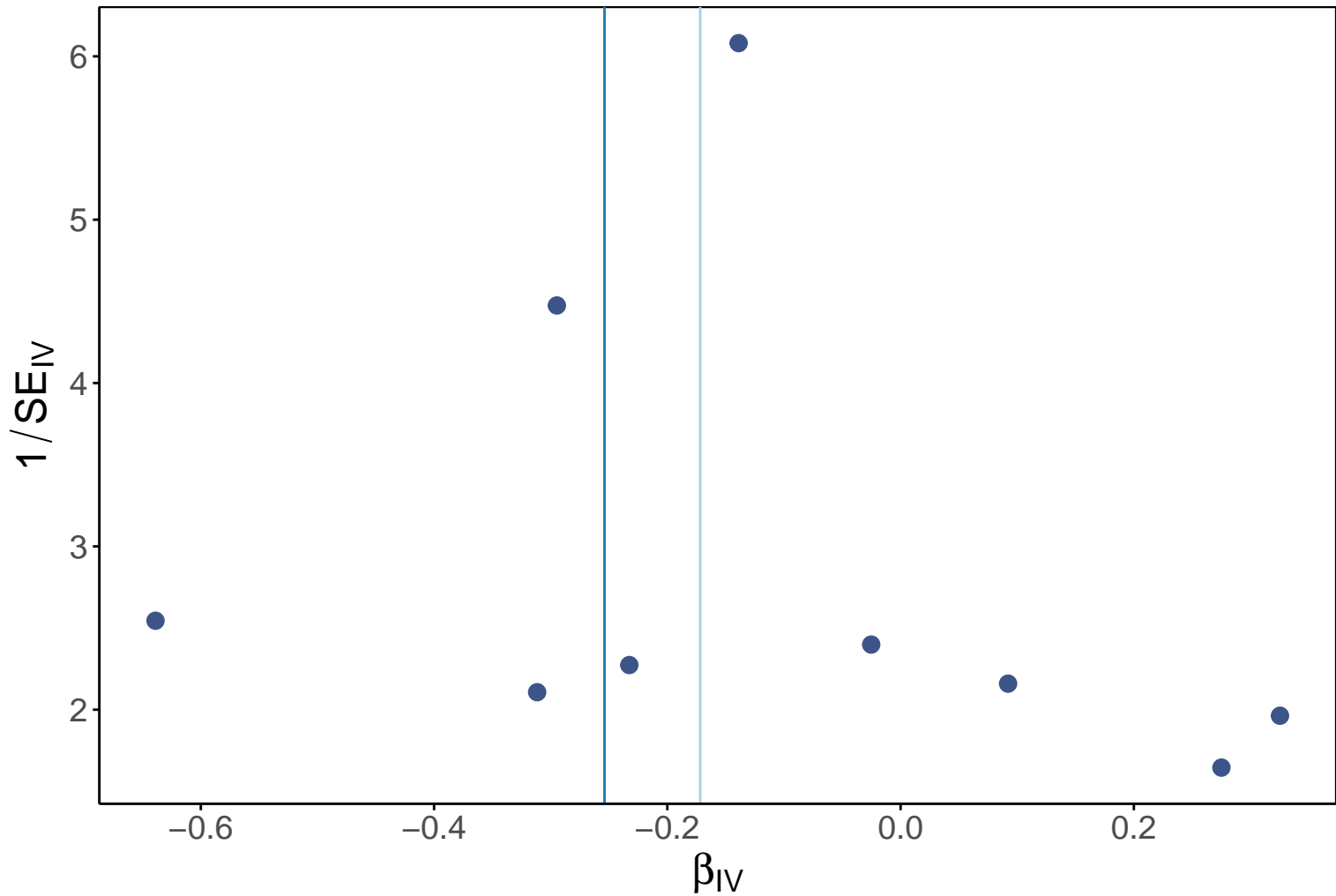

Supplement: Supplementary file 2 [file DataSheet_2.zip › 1. Figure/Figure 4/Figure 4G.pdf]

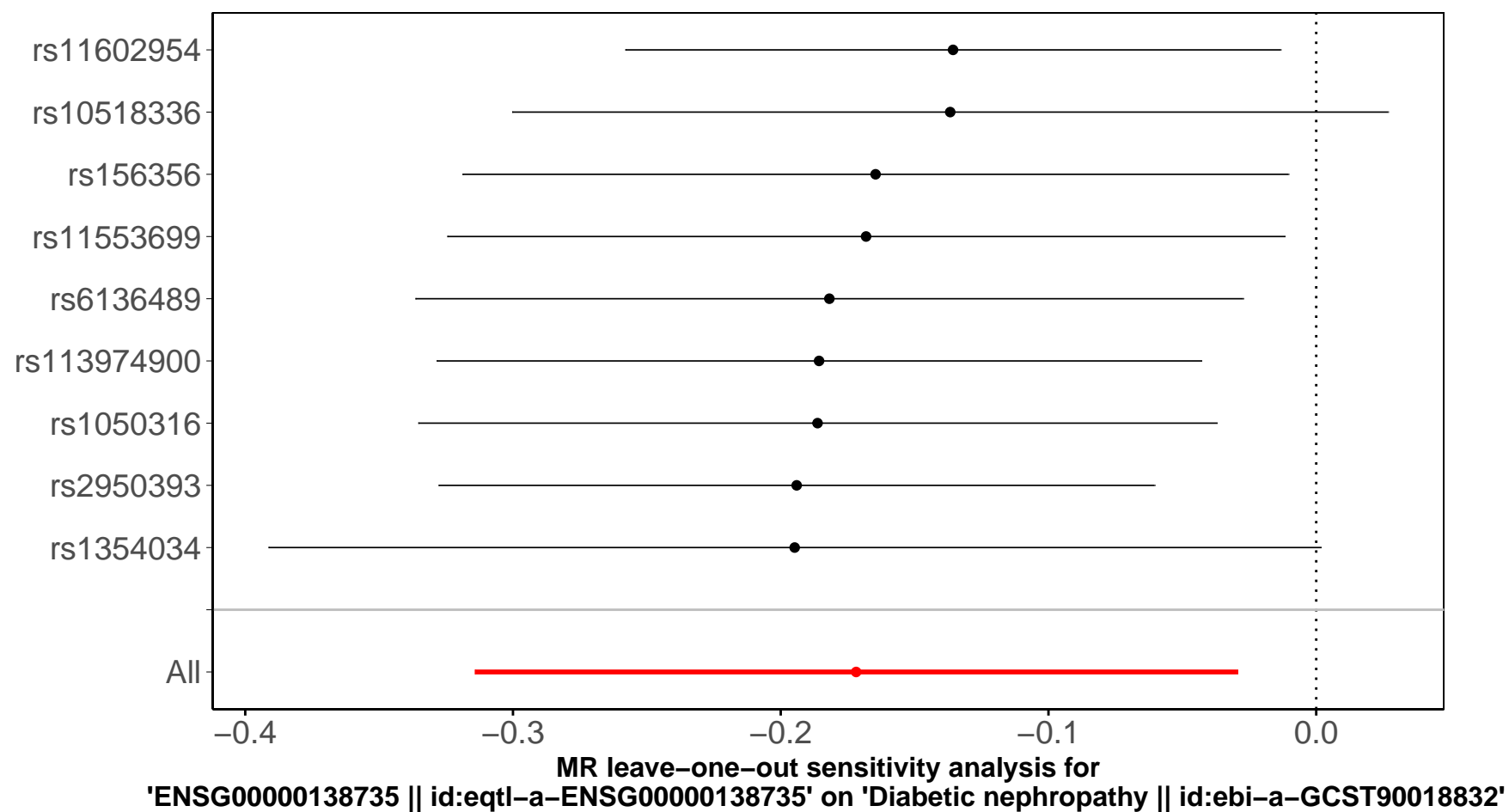

Supplement: Supplementary file 2 [file DataSheet_2.zip › 1. Figure/Figure 4/Figure 4I.pdf]

Points

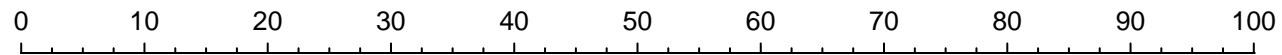

CTSC

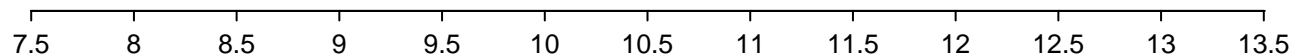

PDE5A

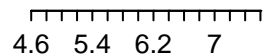

Total Points

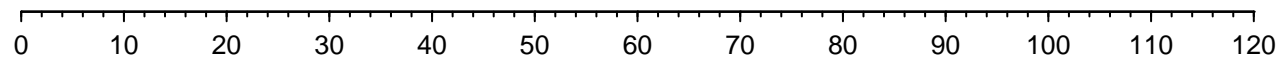

Risk of DN

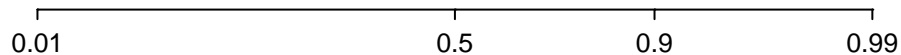

Supplement: Supplementary file 2 [file DataSheet_2.zip › 1. Figure/Figure 5/Figure 5A.pdf]

**ROC Curve**

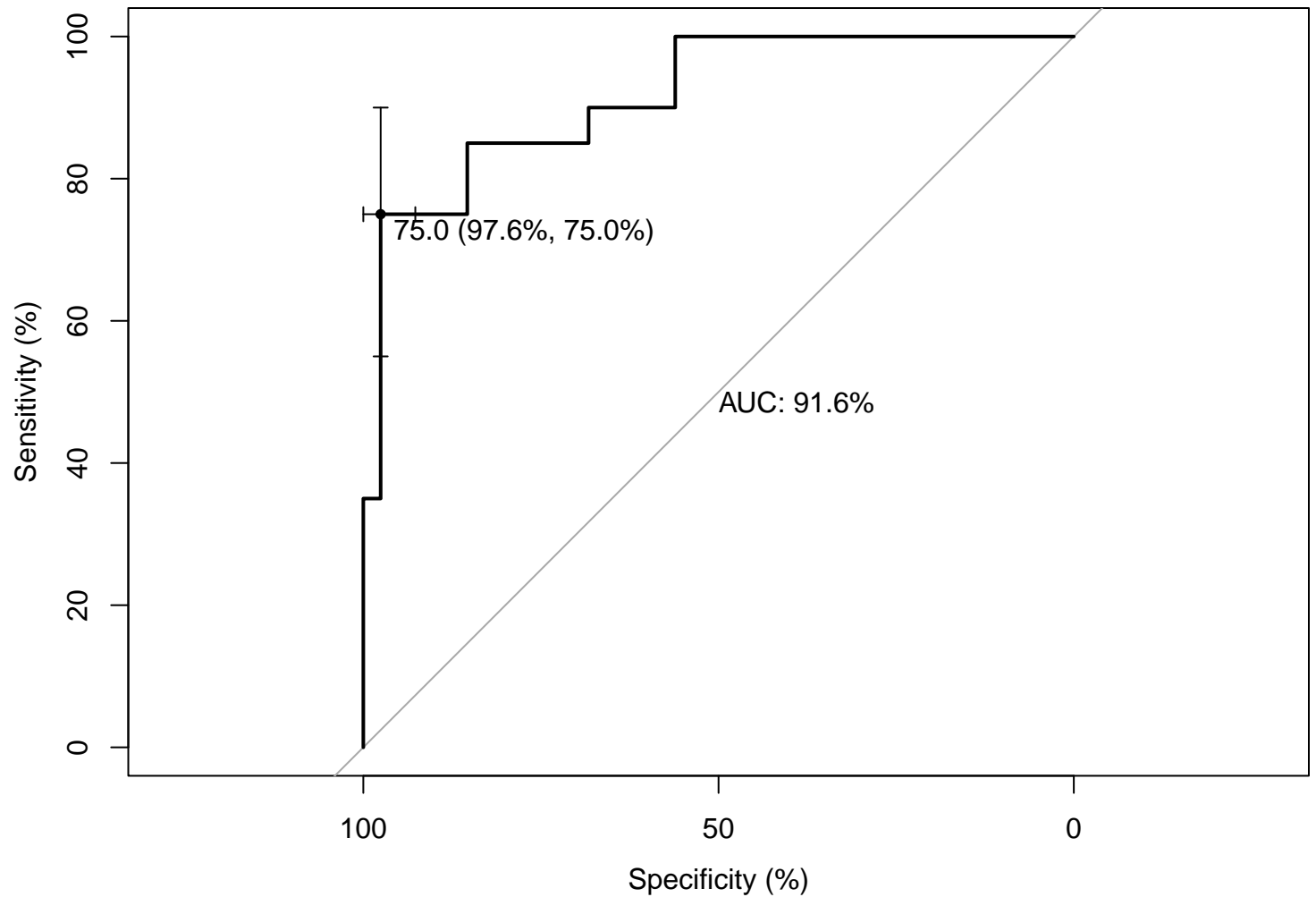

Supplement: Supplementary file 2 [file DataSheet_2.zip › 1. Figure/Figure 5/Figure 5B.pdf]

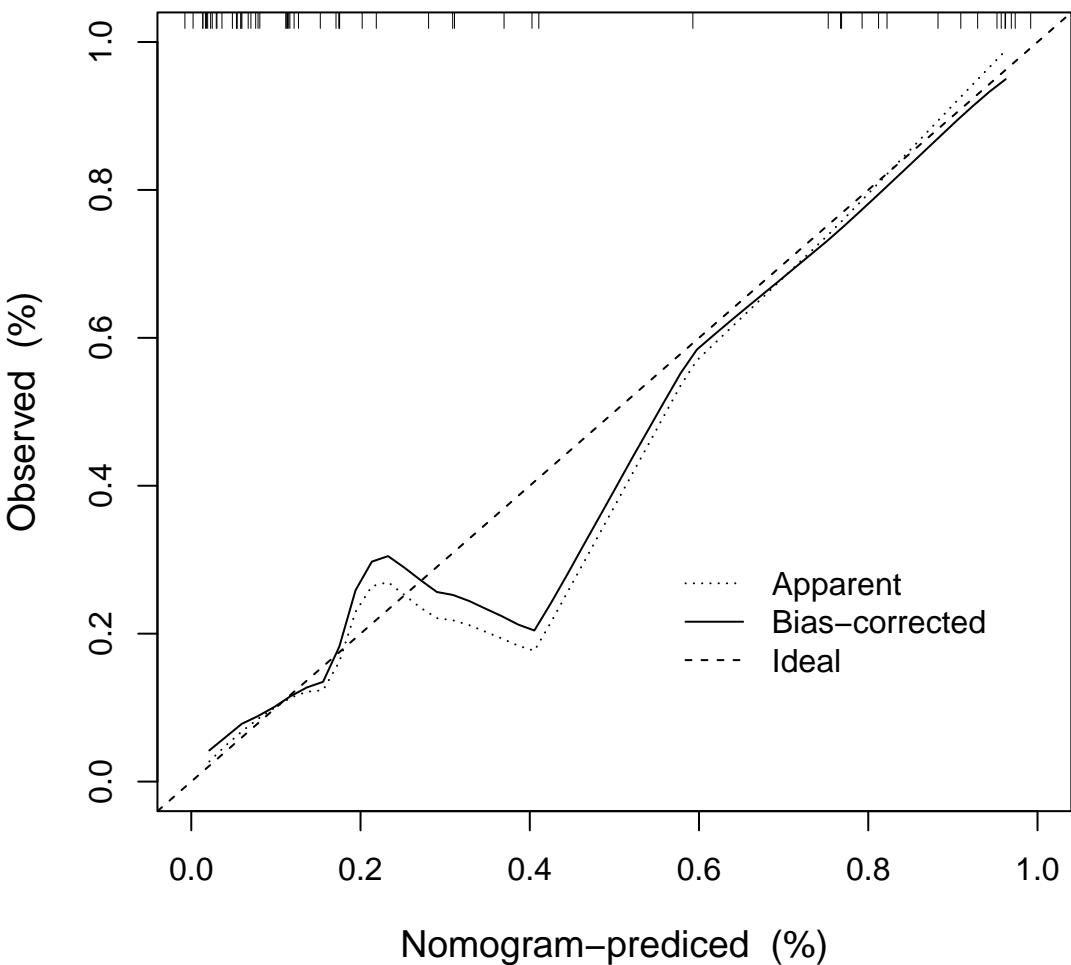

Supplement: Supplementary file 2 [file DataSheet_2.zip › 1. Figure/Figure 5/Figure 5C.pdf]

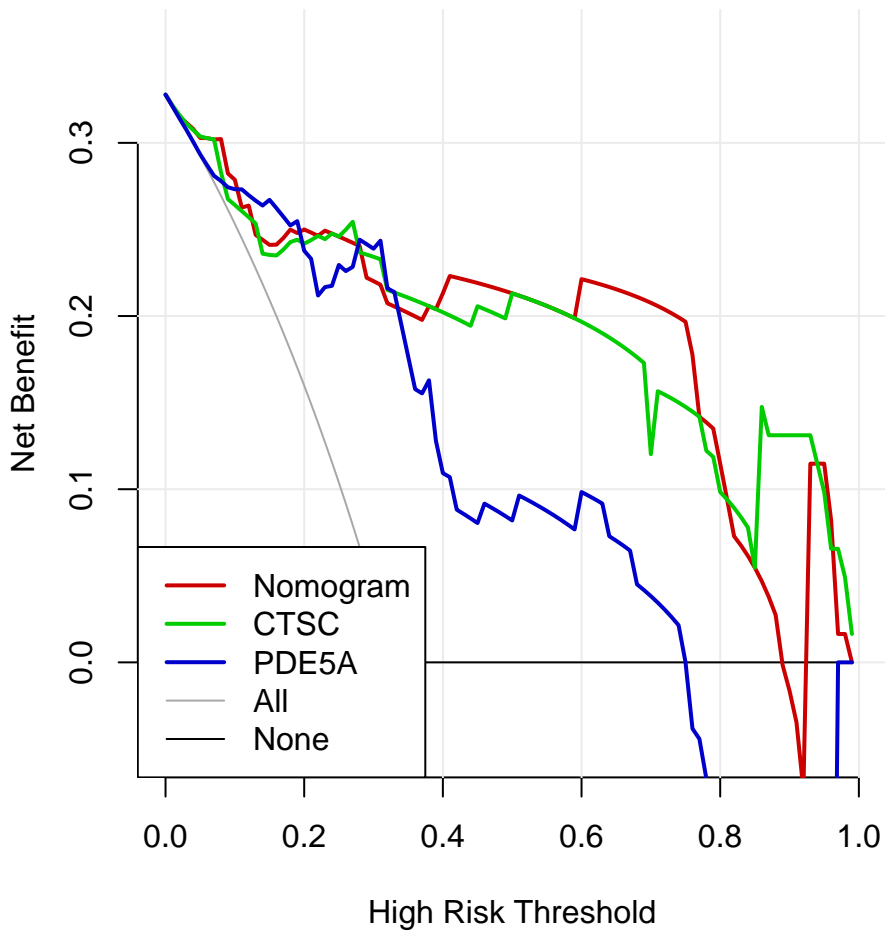

Supplement: Supplementary file 2 [file DataSheet_2.zip › 1. Figure/Figure 5/Figure 5D.pdf]

# PDE5A

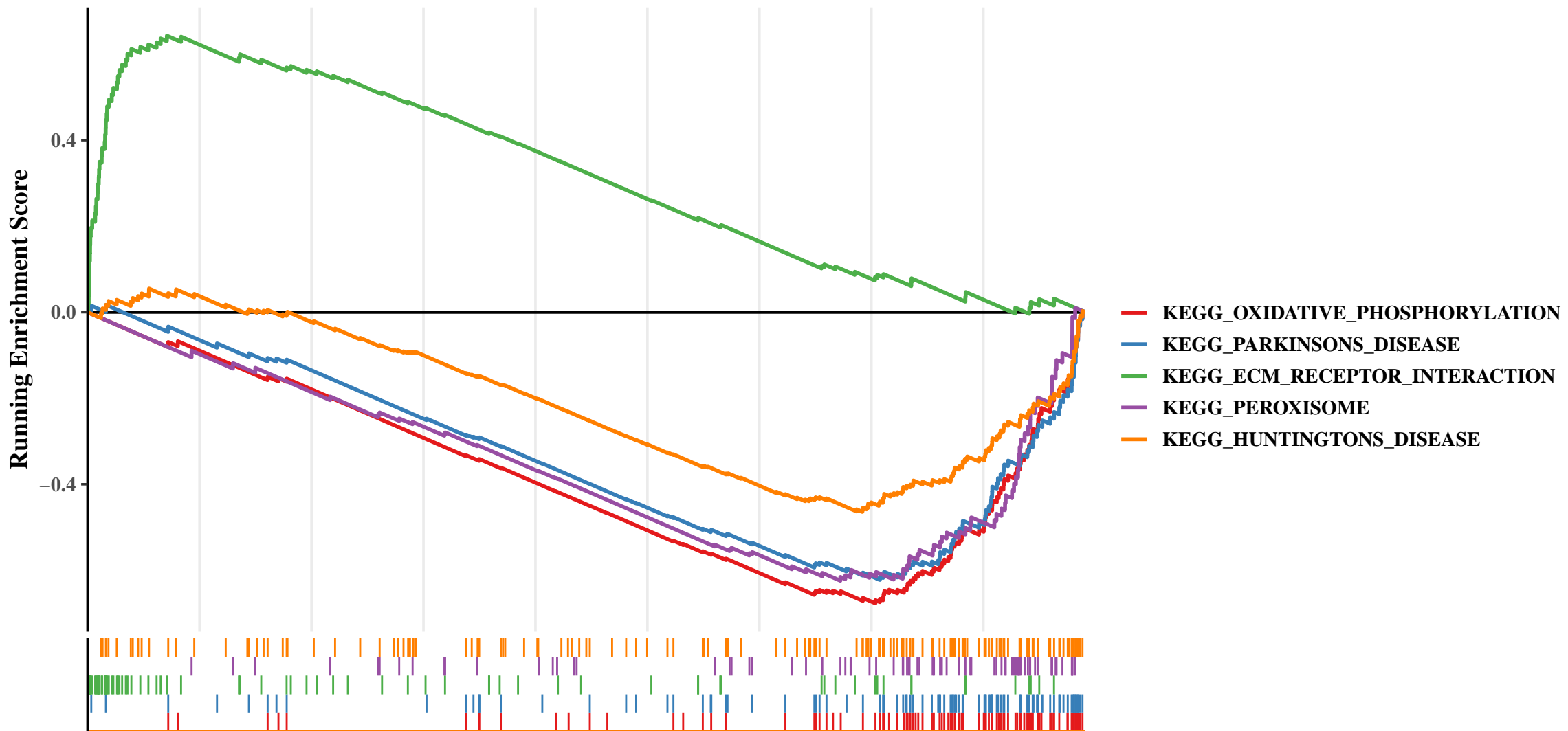

Supplement: Supplementary file 2 [file DataSheet_2.zip › 1. Figure/Figure 6/Figure 6B.pdf]

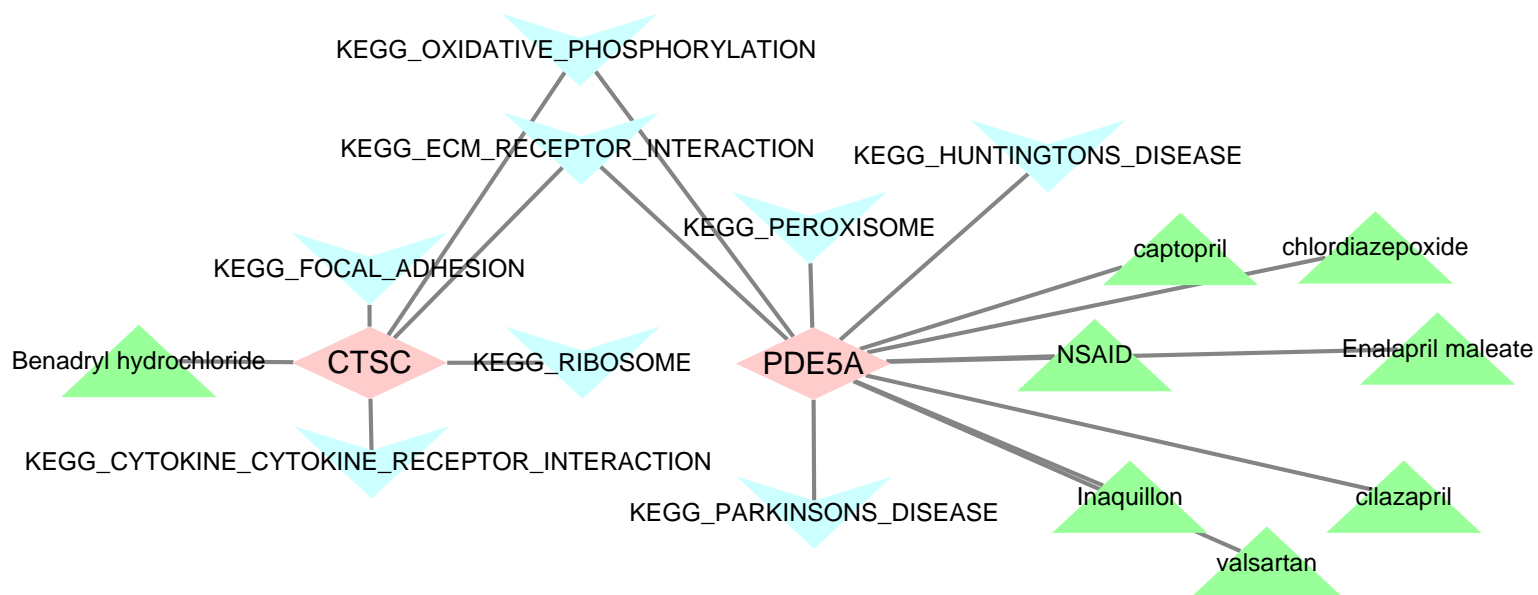

Supplement: Supplementary file 2 [file DataSheet_2.zip › 1. Figure/Figure 6/Figure 6C.pdf]

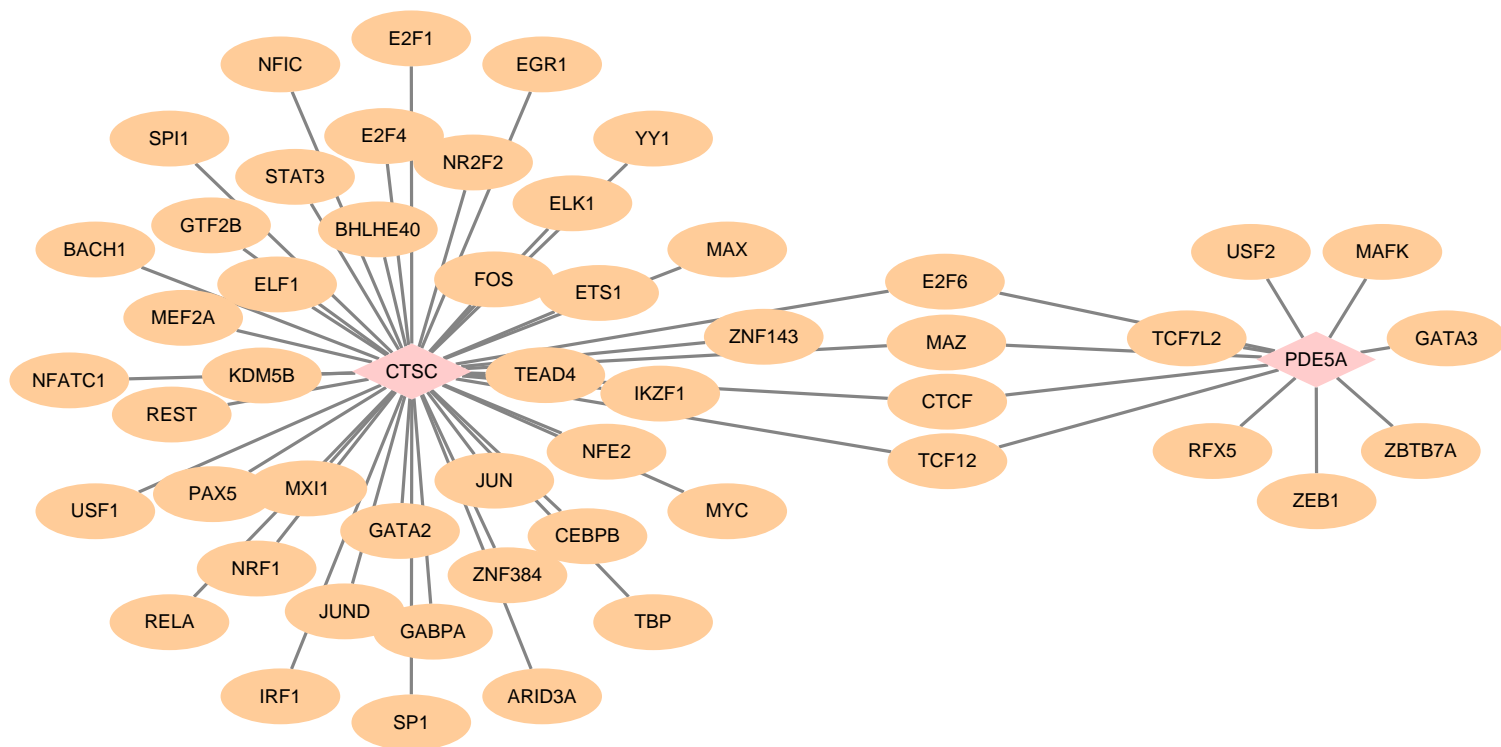

Supplement: Supplementary file 2 [file DataSheet_2.zip › 1. Figure/Figure 7/Figure 7A.pdf]

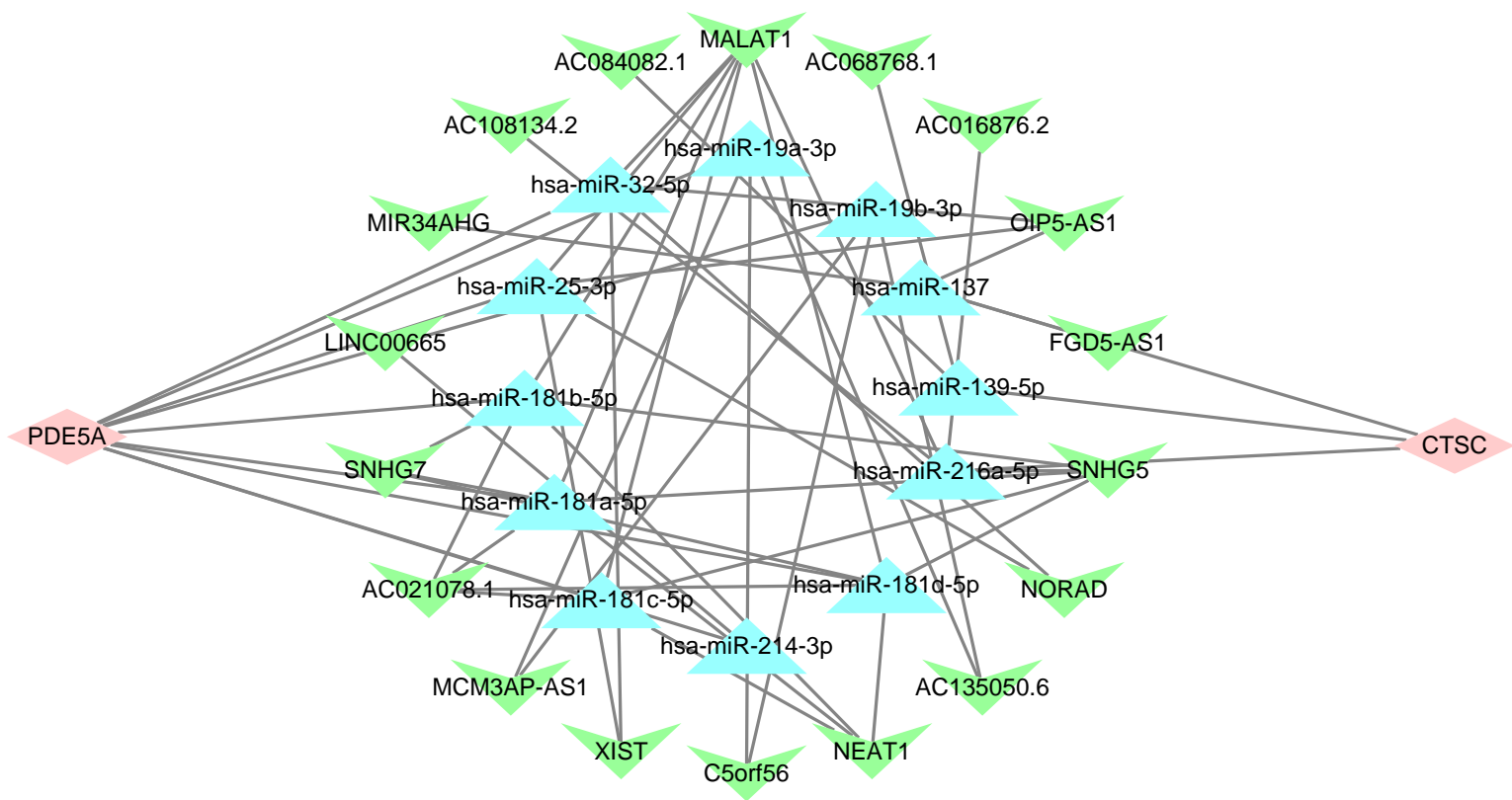

Supplement: Supplementary file 2 [file DataSheet_2.zip › 1. Figure/Figure 7/Figure 7B.pdf]

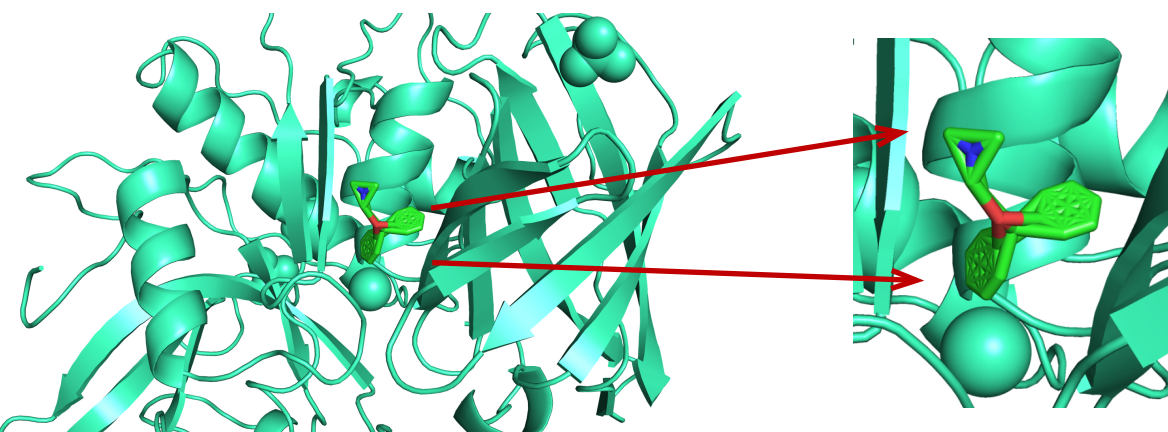

Supplement: Supplementary file 2 [file DataSheet_2.zip › 1. Figure/Figure 8/Figure 8A.pdf]

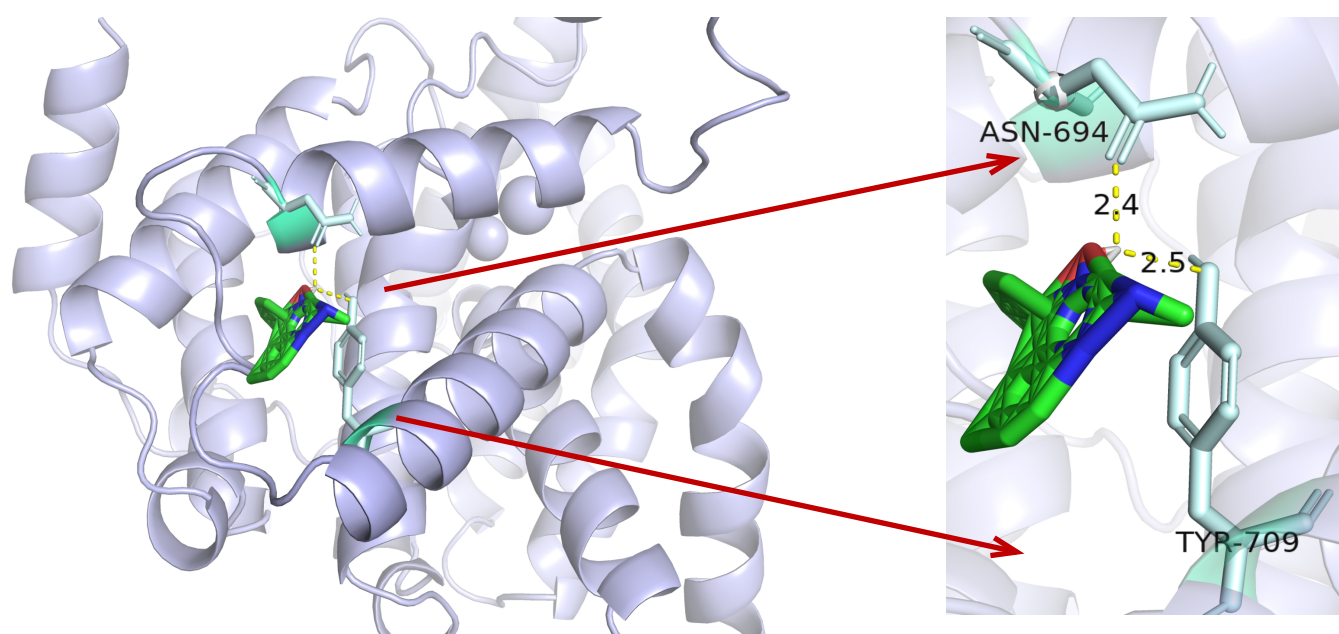

Supplement: Supplementary file 2 [file DataSheet_2.zip › 1. Figure/Figure 8/Figure 8B.pdf]

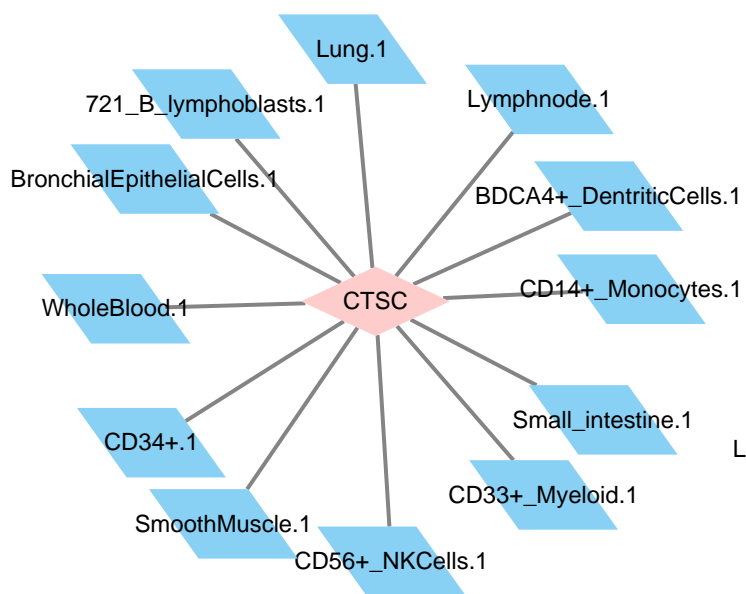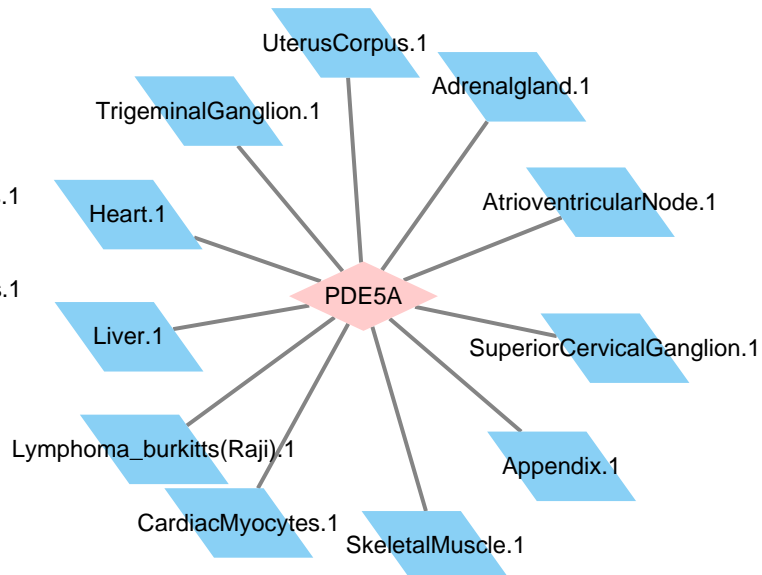

Supplement: Supplementary file 2 [file DataSheet_2.zip › 1. Figure/Figure S/Figure S1/Figure S1.pdf]

**Proportion**

100%  
75%  
50%  
25%  
0%

**CTSC**

**PDE5A**

**Locator**

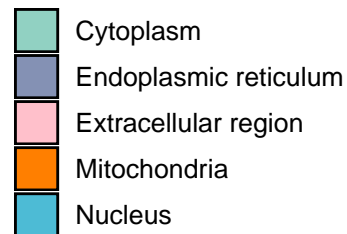

Supplement: Supplementary file 2 [file DataSheet_2.zip › 1. Figure/Figure S/Figure S2/Figure S3.pdf]
