# Supplementary material for: Network pharmacology combined with Mendelian randomization analysis to identify the key targets of renin-angiotensin-aldosterone system inhibitors in the treatment of diabetic nephropathy
Source: Front Endocrinol (Lausanne). 2024 Jan 25;15:1354950. doi: 10.3389/fendo.2024.1354950 (PMC10850565; doi:10.3389/fendo.2024.1354950)
Supplement: Supplementary file 2 [file DataSheet_2.zip › 1. Figure/Figure 4/Figure 4B.pdf]

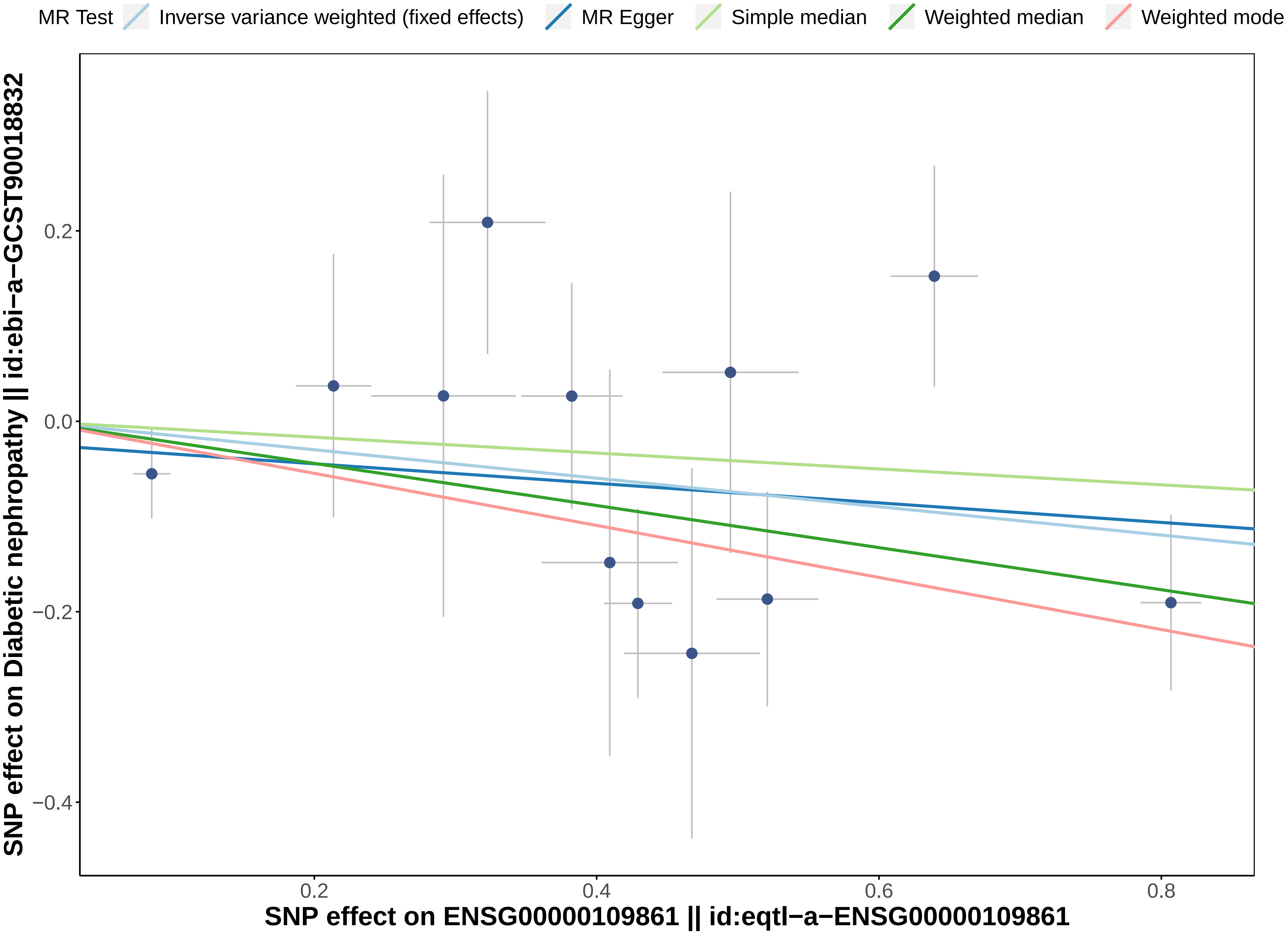

MR Test
Inverse variance weighted (fixed effects)
MR Egger
Simple median
Weighted median
Weighted mode
SNP effect on Diabetic nephropathy || id:ebi−a−GCST90018832
0.2
0.0
−0.2
−0.4
0.2
0.4
0.6
0.8
SNP effect on ENSG00000109861 || id:eqtl−a−ENSG00000109861
