# Supplementary material for: Network pharmacology combined with Mendelian randomization analysis to identify the key targets of renin-angiotensin-aldosterone system inhibitors in the treatment of diabetic nephropathy
Source: Front Endocrinol (Lausanne). 2024 Jan 25;15:1354950. doi: 10.3389/fendo.2024.1354950 (PMC10850565; doi:10.3389/fendo.2024.1354950)
Supplement: Supplementary file 2 [file DataSheet_2.zip › 1. Figure/Figure 4/Figure 4H.pdf]

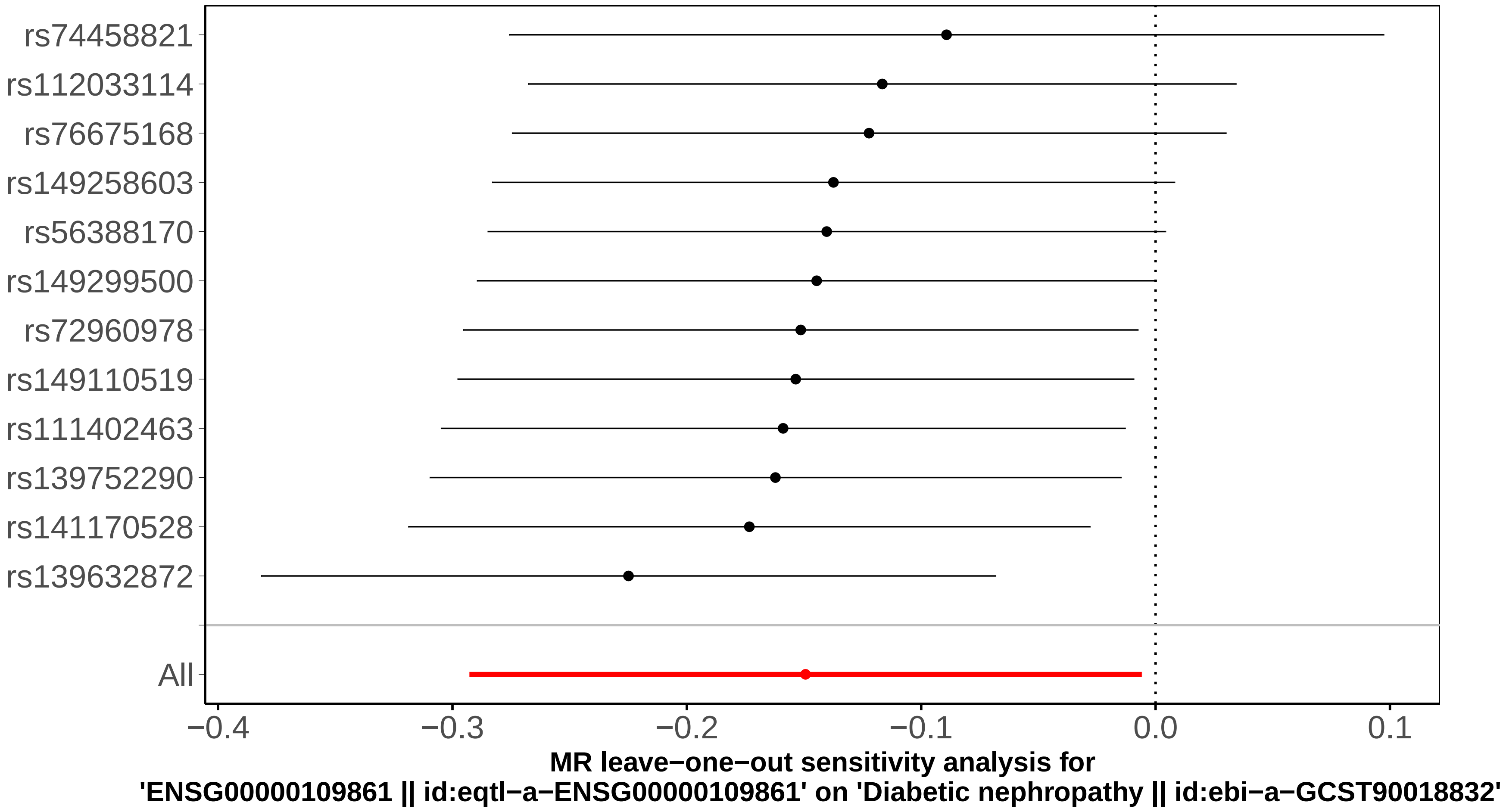
rs74458821
rs112033114
rs76675168
rs149258603
rs56388170
rs149299500
rs72960978
rs149110519
rs111402463
rs139752290
rs141170528
rs139632872
All
−0.4
−0.3
−0.2
−0.1
0.0
0.1
MR leave−one−out sensitivity analysis for
'ENSG00000109861 || id:eqtl−a−ENSG00000109861' on 'Diabetic nephropathy || id:ebi−a−GCST90018832'
