# Supplementary material for: Network pharmacology combined with Mendelian randomization analysis to identify the key targets of renin-angiotensin-aldosterone system inhibitors in the treatment of diabetic nephropathy
Source: Front Endocrinol (Lausanne). 2024 Jan 25;15:1354950. doi: 10.3389/fendo.2024.1354950 (PMC10850565; doi:10.3389/fendo.2024.1354950)
Supplement: Supplementary file 2 [file DataSheet_2.zip › 1. Figure/Figure 6/Figure 6A.pdf]

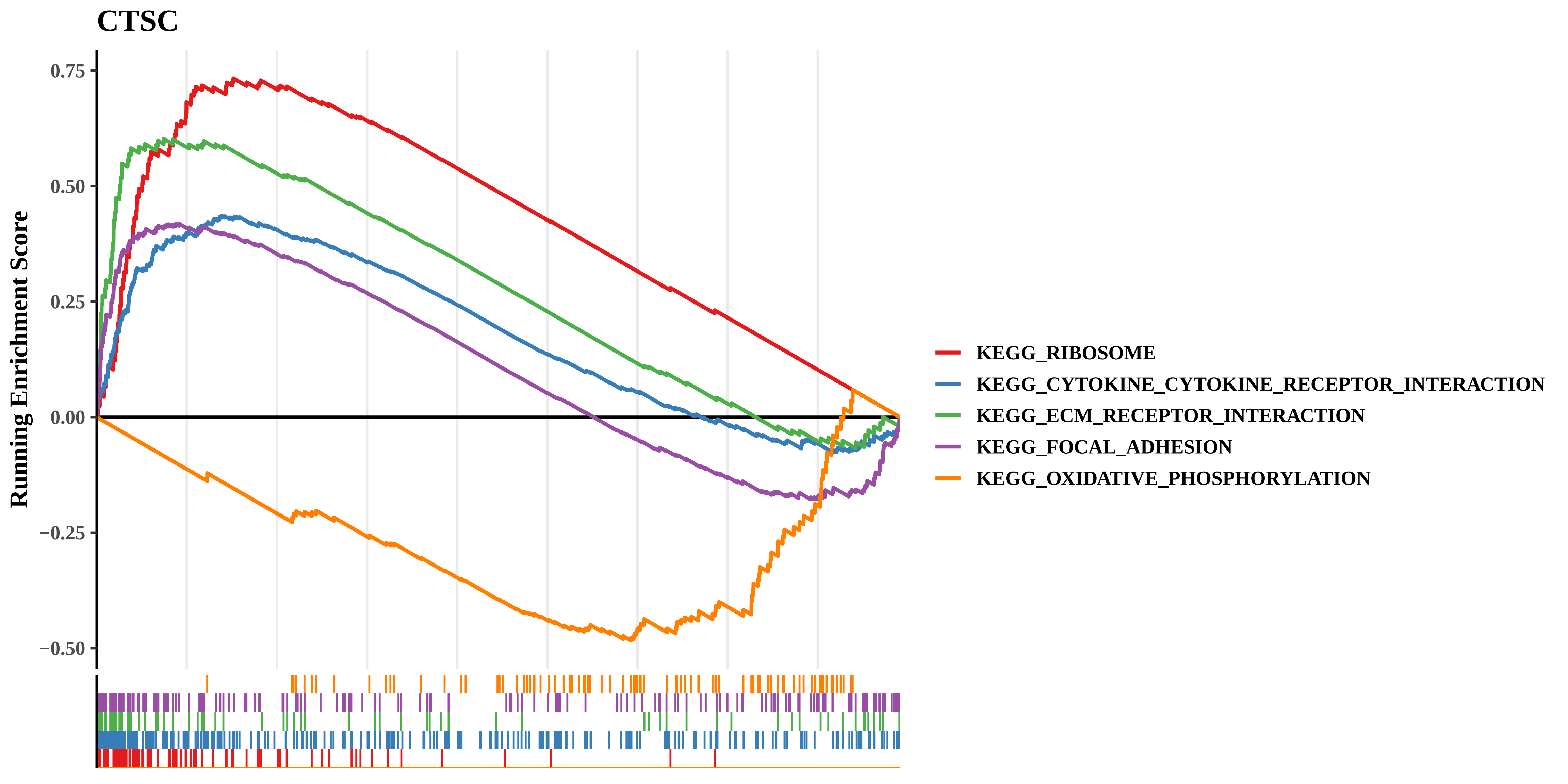
CTSC
Running Enrichment Score
0.75
0.50
0.25
0.00
−0.25
−0.50
KEGG_RIBOSOME
KEGG_CYTOKINE_CYTOKINE_RECEPTOR_INTERACTION
KEGG_ECM_RECEPTOR_INTERACTION
KEGG_FOCAL_ADHESION
KEGG_OXIDATIVE_PHOSPHORYLATION
