# Supplementary material for: Network pharmacology combined with Mendelian randomization analysis to identify the key targets of renin-angiotensin-aldosterone system inhibitors in the treatment of diabetic nephropathy
Source: Front Endocrinol (Lausanne). 2024 Jan 25;15:1354950. doi: 10.3389/fendo.2024.1354950 (PMC10850565; doi:10.3389/fendo.2024.1354950)
Supplement: Supplementary file 3 [file DataSheet_3.zip › 2. Table/2. Table/Table 1/Table 1.docx]

**表1 批量筛选结果**

|  | **id.outcome** | **method** | **nsnp** | **pval** | **pleio_pval** |
| --- | --- | --- | --- | --- | --- |
| eqtl-a-ENSG00000162551 | ebi-a-GCST90018832 | Inverse variance weighted | 5 | 0.017414661 | 0.762383096 |
| eqtl-a-ENSG00000087237 |  |  | 5 | 0.015153531 | 0.822955456 |
| eqtl-a-ENSG00000109861 |  |  | 12 | 0.041192005 | 0.693228083 |
| eqtl-a-ENSG00000170345 |  |  | 3 | 0.002728796 | 0.661371986 |
| eqtl-a-ENSG00000161638 |  |  | 5 | 4.42E-07 | 0.666548208 |
| eqtl-a-ENSG00000138735 |  |  | 9 | 0.018023831 | 0.746067693 |
