# Supplementary material for: Network pharmacology combined with Mendelian randomization analysis to identify the key targets of renin-angiotensin-aldosterone system inhibitors in the treatment of diabetic nephropathy
Source: Front Endocrinol (Lausanne). 2024 Jan 25;15:1354950. doi: 10.3389/fendo.2024.1354950 (PMC10850565; doi:10.3389/fendo.2024.1354950)
Supplement: Supplementary file 3 [file DataSheet_3.zip › 2. Table/2. Table/Table 2/Table 2.docx]

**表2 MR分析结果**

| **outcome** | **exposure** | **Method** | **Pvalue** | **OR** |
| --- | --- | --- | --- | --- |
| ebi-a-GCST90018832 | eqtl-a-ENSG00000109861（CTSC） | MR Egger | 0.492 | 0.902 |
|  |  | IVW | 0.041 | 0.861 |
|  |  | Weighted median | 0.025 | 0.802 |
|  |  | Simple mode | 0.680 | 1.090 |
|  |  | Weighted mode | 0.024 | 0.761 |
|  | eqtl-a-ENSG00000138735  （PDE5A） | MR Egger | 0.371 | 0.776 |
|  |  | IVW | 0.018 | 0.842 |
|  |  | Weighted median | 0.171 | 0.835 |
|  |  | Simple mode | 0.331 | 0.814 |
|  |  | Weighted mode | 0.231 | 0.832 |
