# Supplementary material for: Network pharmacology combined with Mendelian randomization analysis to identify the key targets of renin-angiotensin-aldosterone system inhibitors in the treatment of diabetic nephropathy
Source: Front Endocrinol (Lausanne). 2024 Jan 25;15:1354950. doi: 10.3389/fendo.2024.1354950 (PMC10850565; doi:10.3389/fendo.2024.1354950)
Supplement: Supplementary file 3 [file DataSheet_3.zip › 2. Table/2. Table/Table 3/Table 3.docx]

**表3 异质性检验**

| **outcome** | **exposure** | **method** | **Q** | **Q_df** | **Q_pval** |
| --- | --- | --- | --- | --- | --- |
| ebi-a-GCST90018832 | eqtl-a-ENSG00000109861（CTSC） | MR Egger | 14.049 | 10 | 0.171 |
|  |  | Inverse variance weighted | 14.280 | 11 | 0.218 |
|  | eqtl-a-ENSG00000138735  （PDE5A） | MR Egger | 3.689 | 7 | 0.815 |
|  |  | Inverse variance weighted | 3.803 | 8 | 0.874 |
