# Supplementary material for: Network pharmacology combined with Mendelian randomization analysis to identify the key targets of renin-angiotensin-aldosterone system inhibitors in the treatment of diabetic nephropathy
Source: Front Endocrinol (Lausanne). 2024 Jan 25;15:1354950. doi: 10.3389/fendo.2024.1354950 (PMC10850565; doi:10.3389/fendo.2024.1354950)
Supplement: Supplementary file 3 [file DataSheet_3.zip › 2. Table/2. Table/Table 4/Table 4.docx]

**表4 水平多效性检验**

|  | **exposure** | **egger_intercept** | **se** | **pval** |
| --- | --- | --- | --- | --- |
| ebi-a-GCST90018832 | eqtl-a-ENSG00000109861（CTSC） | -0.024 | 0.059 | 0.693 |
|  | eqtl-a-ENSG00000138735  （PDE5A） | 0.014 | 0.041 | 0.746 |
