# Supplementary material for: Network pharmacology combined with Mendelian randomization analysis to identify the key targets of renin-angiotensin-aldosterone system inhibitors in the treatment of diabetic nephropathy
Source: Front Endocrinol (Lausanne). 2024 Jan 25;15:1354950. doi: 10.3389/fendo.2024.1354950 (PMC10850565; doi:10.3389/fendo.2024.1354950)
Supplement: Supplementary file 3 [file DataSheet_3.zip › 2. Table/2. Table/Table 5/Table 5.docx]

| **outcome** | **exposure** | **Method** | **Pvalue** | **OR** |
| --- | --- | --- | --- | --- |
| ukb-b-4963 | eqtl-a-ENSG00000166825 (ANPEP) | MR Egger | 0.000 | 0.000 |
|  |  | IVW | 0.000 | 3.82E-07 |
|  |  | Weighted median | 0.000 | -4.78E-06 |
|  |  | Simple mode | 0.000 | 0.001 |
|  |  | Weighted mode | 0.000 | 4.44E-06 |
|  | eqtl-a-ENSG00000115232 (IGTA4) | MR Egger | 0.637 | 0.9998 |
|  |  | IVW | 0.000 | 0.9997 |
|  |  | Weighted median | 0.128 | 0.9997 |
|  |  | Simple mode | 0.350 | 0.9995 |
|  |  | Weighted mode | 0.320 | 0.9997 |
|  | eqtl-a-ENSG00000073756(PTGS2) | MR Egger | 0.320 | 1.001 |
|  |  | IVW | 0.004 | 1.001 |
|  |  | Weighted median | 0.007 | 1.001 |
|  |  | Simple mode | 0.169 | 1.001 |
|  |  | Weighted mode | 0.081 | 1.001 |

**Table 5 Mendelian randomization (MR) analysis for causal relationship of three key targets (PTGS2, ITGA4, and ANPEP) and AKI.**
