# Supplementary material for: Network pharmacology combined with Mendelian randomization analysis to identify the key targets of renin-angiotensin-aldosterone system inhibitors in the treatment of diabetic nephropathy
Source: Front Endocrinol (Lausanne). 2024 Jan 25;15:1354950. doi: 10.3389/fendo.2024.1354950 (PMC10850565; doi:10.3389/fendo.2024.1354950)
Supplement: Supplementary file 4 [file DataSheet_4.docx]

**Supplementary information**


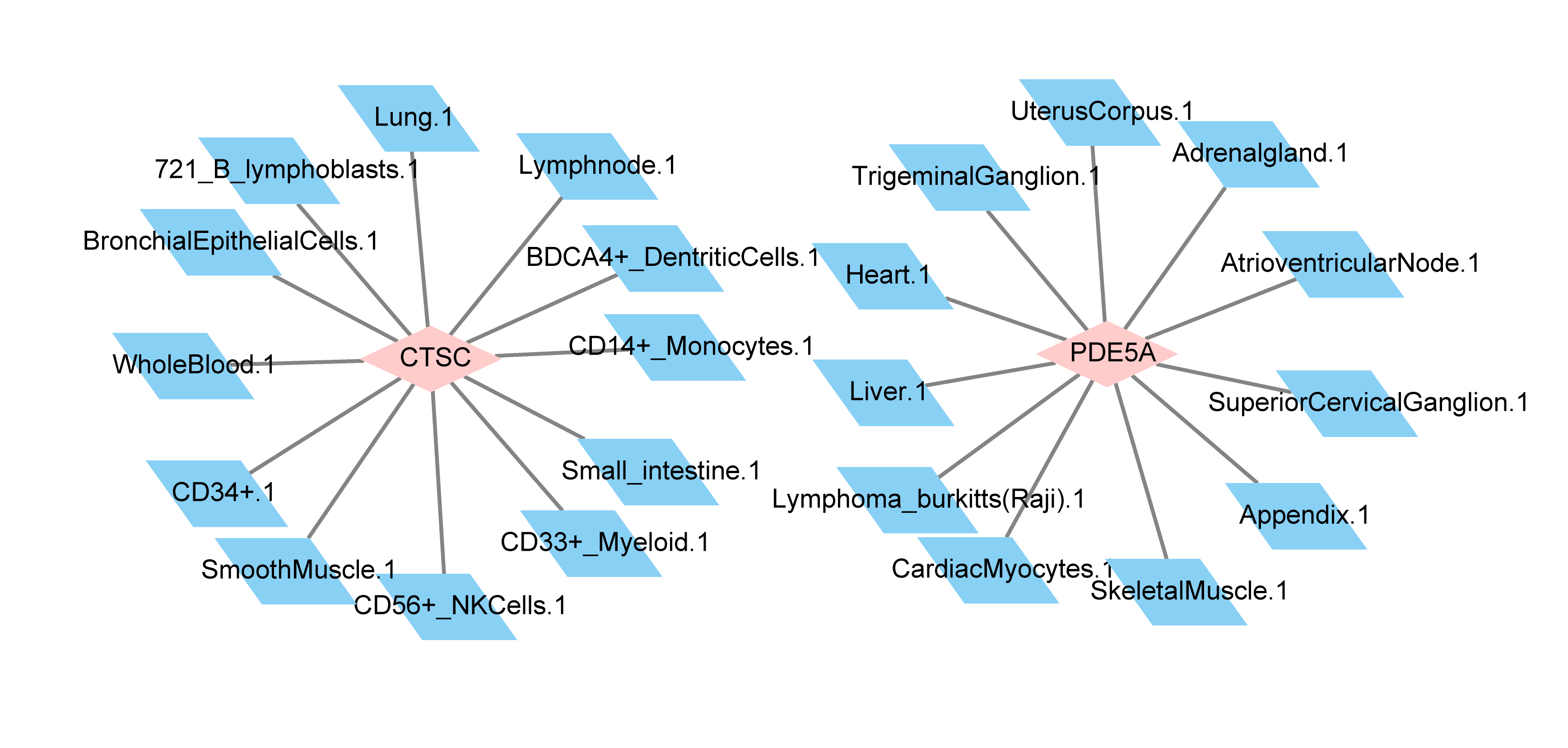


**Figure S1.** The key target–organ network for the mRNA levels of CTSC and PDE5A. Pink is the target gene, and blue is the organ or tissue.

**
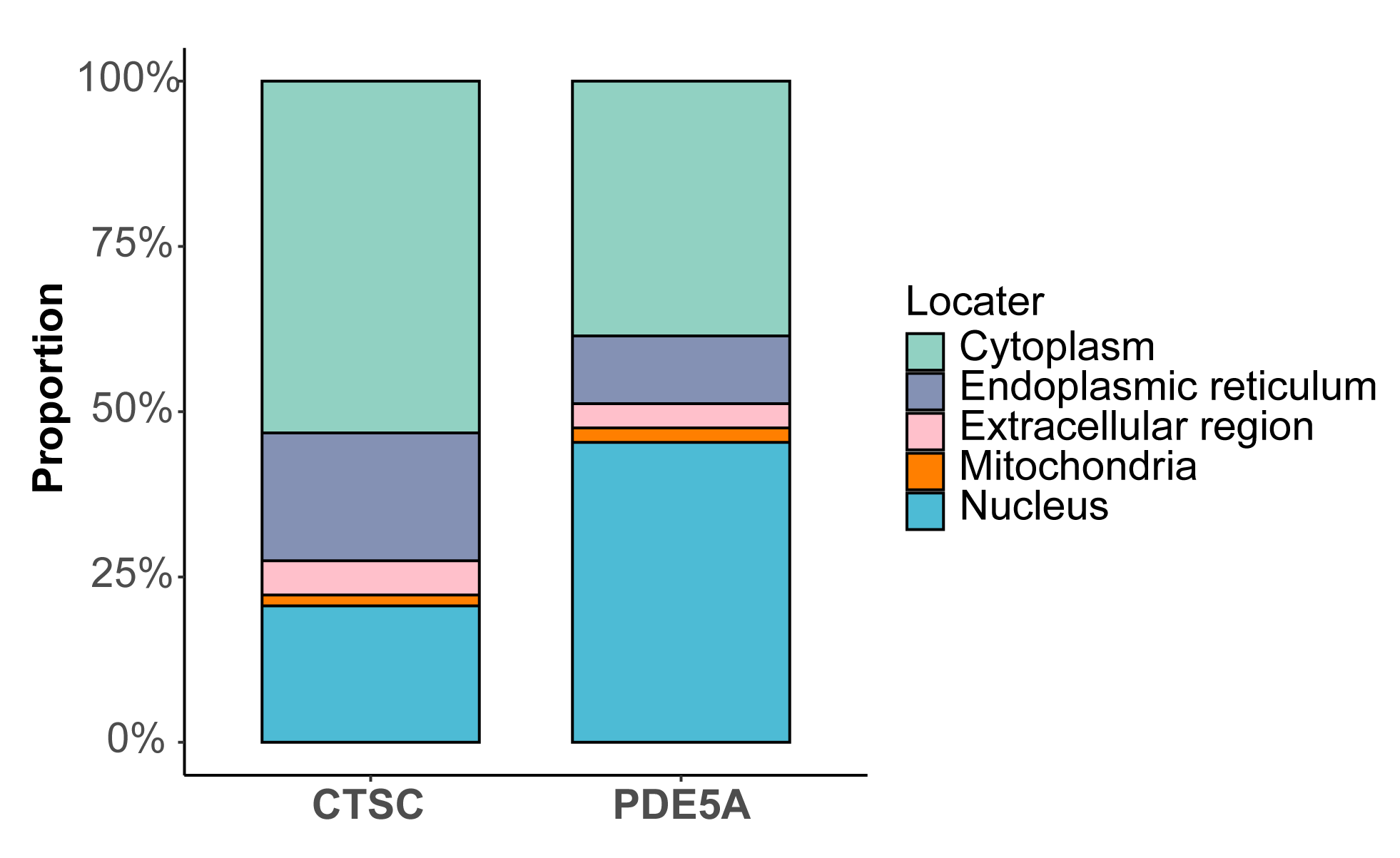
**

**Figure S2 Proportion bar chart for the subcellular localization of key targets.**

**Table S1 F statistic value**

**
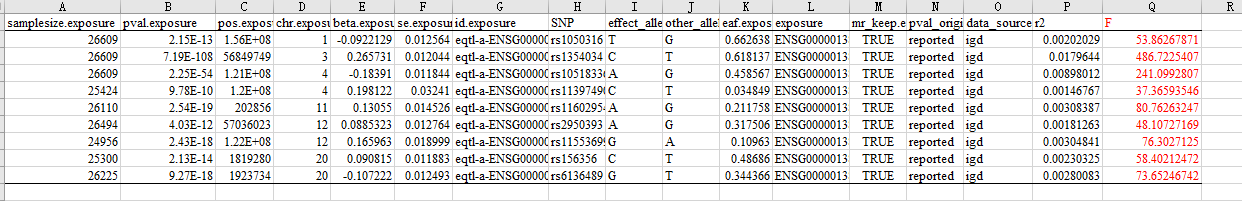
**

**Table S2. Lists of the 60 candidate genes.**

| **Gene** |
| --- |
| ABCC9 |
| ACE2 |
| ADAM10 |
| ADAMTS5 |
| ALDH2 |
| ALPL |
| ANPEP |
| AXL |
| C5AR1 |
| CA2 |
| CASP3 |
| CES2 |
| CETP |
| CPT1A |
| CTSC |
| CXCR1 |
| CXCR2 |
| DPP4 |
| EDNRA |
| EDNRB |
| EPHX2 |
| FABP1 |
| FABP3 |
| FABP4 |
| FABP5 |
| FOS |
| GSTK1 |
| HDAC9 |
| HIF1A |
| HNF4A |
| HSD11B2 |
| HSPA1A |
| IDH1 |
| IGFBP3 |
| INSR |
| ITGA4 |
| ITGA5 |
| JUN |
| KDR |
| MIF |
| MME |
| MMP14 |
| MMP2 |
| MMP7 |
| NAMPT |
| PDE5A |
| PLA2G7 |
| PLAT |
| PLG |
| PTGS1 |
| PTGS2 |
| SERPINE1 |
| SLC22A12 |
| SLC5A2 |
| SLC6A2 |
| SORT1 |
| TACR1 |
| TNNT2 |
| TTR |
| TYRO3 |
